# Liquid Biopsy and Multi‐Cancer Early Detection Tests for Cancer Screening: A Scoping Review

**DOI:** 10.1111/cge.70249

**Published:** 2026-09-25

**Authors:** Tessa Robinson, Marian Saab, Lily Thao Nguyen, Pooja Kochar, Olivia Meggetto, Jill Tinmouth, Meghan J. Walker

**Affiliations:** ^1^ Division of Prevention & Screening, Population and Community‐Based Care Ontario Health Toronto Ontario Canada; ^2^ Dalla Lana School of Public Health University of Toronto Toronto Ontario Canada; ^3^ Department of Medicine Sunnybrook Health Sciences Centre Toronto Ontario Canada; ^4^ Department of Medicine University of Toronto Toronto Ontario Canada

**Keywords:** cancer screening, cancer screening tests, liquid biopsy, multi‐cancer early detection, scoping review

## Abstract

Liquid biopsy constitutes a range of tests that use biological fluids to detect cancer. Research is now targeting their use for cancer screening. This is the first review of the evidence on liquid biopsy tests across a range of analytical parameters, biomarkers, and cancer types for cancer screening. A scoping review was conducted using JBI methodology for scoping reviews. MEDLINE and targeted websites were searched for studies published in 2014 or later on the use of liquid biopsy and multi‐cancer early detection tests for screening. A total of 104 studies were included, covering a wide range of study designs. Several types of biomarkers (e.g., cell‐free DNA, circulating tumor DNA) and analytical parameters were used. Most studies addressed test performance outcomes, while fewer addressed clinical outcomes, implementation considerations, and cost‐effectiveness. No empirical studies on cancer‐specific mortality were found. A large body of literature was identified that covered a range of cancer types, biomarkers, analytical parameters, and outcomes. Critical gaps in the literature were demonstrated, including evidence on clinical outcomes such as cancer‐specific mortality and potential harms that currently preclude the implementation of liquid biopsy tests for cancer screening. Findings of this review can be used to guide the necessary future research.

## Graphical abstract

There is a large body of evidence on liquid biopsy and multi‐cancer detection tests for cancer screening. Most studies addressed test performance outcomes, while fewer addressed clinical outcomes, implementation, and cost‐effectiveness. No studies evaluated cancer mortality. Evidence on mortality is needed before these tests should be used for screening.

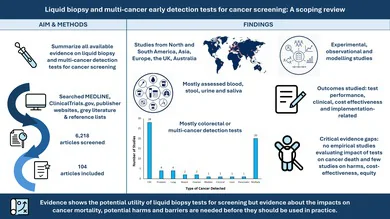

## 1 Introduction

Cancer represents a significant burden of disease around the world, attributable to approximately 20 million new cases and 9.7 million deaths in 2022 [1]. Cancer screening can reduce this burden by identifying pre‐cancers or early‐stage cancers among the asymptomatic population, when treatment may be more effective. There is consistent systematic review evidence to suggest that the potential benefits of screening for breast, cervical, colorectal (CRC), and lung cancer outweigh the potential harms for specific populations [2, 3, 4, 5, 6]. Many jurisdictions have already implemented or are planning to implement organized screening programs for these cancers [7, 8, 9, 10]. Organized screening for other types of cancer is not available in most jurisdictions due to insufficient evidence that the benefits of screening using currently available tests outweigh the potential harms. This includes a lack of evidence for reductions in cancer‐specific mortality, which is a critical criterion for establishing organized cancer screening programs [11].

There is growing international attention on the potential use of liquid biopsy for cancer screening. Liquid biopsy is an all‐encompassing term that refers to a range of non‐invasive tests that use biological fluids, such as blood, saliva, stool, and urine for cancer detection [12]. These tests allow for the detection of cancer‐specific biomarkers that characterize changes in the genomic and proteomic profile of normal tissues, including circulating tumor DNA (ctDNA), cell‐free DNA (cfDNA), circulating tumor cells (CTCs), and tumor‐derived extracellular vesicles [12, 13, 14]. They may use single biomarkers [15] or combine multiple biomarkers [16] and can be used to detect one specific type of cancer or multiple types [13] (commonly referred to as multi‐cancer early detection [MCED] tests) [17]. In the context of cancer screening, liquid biopsy tests have been suggested for the early detection of cancer among asymptomatic individuals, with the goal of improving cancer‐specific morbidity and mortality [18].

If a favorable benefit‐to‐harm ratio can be demonstrated, liquid biopsy tests have the potential to transform cancer screening. They have the potential to support early detection of cancers that do not have established screening tests [19] as well as complement existing screening programs [14]. It has also been suggested that liquid biopsy tests could address barriers in equitable access to screening programs that can result in reduced participation [20]. Many current population‐based screening programs rely on screening modalities that are invasive, require administration by trained health personnel, and may have limited access in rural and remote locations [20, 21]. The use of liquid biopsy tests for screening may help overcome geographical barriers and health human resource challenges that contribute to current health system capacity constraints [20]. Moreover, they may offer non‐invasive alternatives to standard tests (e.g., Pap/HPV tests, mammography), potentially reducing fear and discomfort and leading to improved participation and retention in existing cancer screening programs [20].

Given the rapid technological advancements and mounting scientific inquiry occurring in this field, there is a need to understand the existing body of literature to determine the current and expected trajectory of the evidence. While previous systematic reviews have assessed the utility of liquid biopsies, most have focused on single types of analytical parameters and biomarkers and often review liquid biopsy for the purposes of cancer diagnosis, disease progression, and monitoring treatment response [22, 23, 24]. A recent systematic review was also published on the benefits, harms, and accuracy of blood‐based MCED tests for cancer screening [25]. A gap remains in mapping the current evidence on liquid biopsy tests in terms of their utility in screening for cancer in asymptomatic populations across a wide range of test analytical parameters, biomarkers, and cancer types.

When evaluating whether a new test should be implemented for population‐based screening, it must first be determined whether the correct types of evidence exist to inform that evaluation. Evidence on the benefits (including cancer‐specific morbidity and mortality) and harms (such as false positive or false negative results, benign invasive procedures, overdiagnosis) of screening is essential. It is also important to consider evidence on test performance (e.g., sensitivity, specificity), cost‐effectiveness, perceptions, equity, and implementation barriers and facilitators. Scoping reviews provide a systematic mapping of diverse types of available literature to generate a fulsome understanding of the evidence landscape that exists for a given topic, including any critical research gaps [26]. A scoping review was conducted to identify and summarize the available evidence on liquid biopsy and MCED tests for cancer screening among asymptomatic adults to assess whether the body of evidence was suitable for further evaluation with respect to implementation in the population‐based screening context. It also aimed to identify gaps in the current evidence base to serve as a foundation for the development of future research on this topic.

## 2 Materials and Methods

This study used rapid scoping review methodology in accordance with scoping review guidelines set forth by the JBI [27]. A protocol outlining all aspects of the review was developed a priori and registered with Open Science Framework [28]. This review is reported according to the Preferred Reporting Items for Systematic Reviews and Meta‐analyzes extension for scoping reviews (Table S1) [29]. This study is a scoping review of published research and is therefore exempt from ethics review.

### 2.1 Research Questions

The primary research question for this scoping review was:

1. What is the available evidence on liquid biopsy and MCED tests for the purposes of cancer screening?

The secondary research questions were:

2 What types of evidence (e.g., experimental, observational) have examined liquid biopsy and MCED tests for the purposes of cancer screening?
3 What types of molecular biomarkers (e.g., cfDNA) and analytical parameters (e.g., methylation patterns) do liquid biopsy and MCED tests use for cancer screening?
4 What types of cancers do liquid biopsy and MCED tests used for cancer screening detect?
5 What outcomes (e.g., clinical outcomes, cost‐effectiveness) and key findings have been reported for the purposes of cancer screening?
6 Are there any clinical trials or studies in‐progress examining liquid biopsy and MCED tests for the purposes of cancer screening?

### 2.2 Eligibility Criteria

Studies were included in this review if they were published in English and included adults aged 18 and older who were not experiencing cancer‐related symptoms. They were required to report on liquid biopsy or MCED tests that analyzed biological fluids (e.g., blood, urine, saliva) for the purposes of cancer screening. Outcomes of interest included, but were not limited to, clinical outcomes (e.g., cancer‐specific mortality, detection rate), test characteristics (e.g., sensitivity, specificity), and health economics (e.g., cost‐effectiveness, cost‐utility). To reflect the most recent evidence, only documents published on or after 2014 were included due to the advancement in liquid biopsy technology in the last 10 years [30]. Only studies that were conducted in countries rated as high or very high on the Human Development Index [31] were included to ensure applicability of findings to contexts in which most organized cancer screening programs take place. Only primary research studies not captured by included systematic or scoping reviews were included. A full description of the inclusion and exclusion criteria for this review can be found in Table S2.

### 2.3 Data Sources and Search Strategy

For this review, searches of MEDLINE (Ovid) and the websites of relevant scientific journals, professional organizations, and governmental bodies were conducted. The MEDLINE search strategy was developed in consultation with a medical librarian (Table S3). Review team members with expertise in cancer screening (OM, MW, JT) and the Canadian Agency for Drugs and Technologies in Health's Grey Matter checklist [32] were consulted to generate a list of websites for the targeted search (Table S4). The reference lists of included documents were also reviewed to identify citations that were missed by the electronic database and targeted website searches. To identify ongoing clinical trials, the ClinicalTrials.gov database was searched using cancer as the condition/disease and liquid biopsy, multicancer early detection tests, and MCED as the intervention/treatment.

### 2.4 Selection Process

All citations identified through the electronic database, targeted website, and hand searches were uploaded into DistillerSR, a web‐based systematic review software [33] and duplicates removed. During title and abstract screening, two artificial intelligence (AI) tools in DistillerSR were utilized to streamline this stage of the screening process. First, the *AI Re‐Rank* feature was used so that references with a greater likelihood of inclusion appeared earlier in the screening process. After one reviewer (MS) screened the titles and abstracts of 20% of citations, the *AI Preview and Rank* feature was employed. This feature assigned scores to citations between zero and one that reflected the likelihood of inclusion, based on previous decisions made by the manual reviewer. For example, references with a low confidence of inclusion were assigned lower scores (closer to zero) and those with high confidence of inclusion were assigned higher scores (closer to one). A single reviewer (MS) then reviewed the titles and abstracts of the remaining citations, using the assigned scores to assist with screening decisions. A second reviewer (TR) verified 30% of citations excluded at the title and abstract stage. Citations marked for inclusion by either reviewer moved through to full‐text screening. Full‐text screening of citations was then completed by a single reviewer (MS, TR, PK) with verification of 30% of exclusions by a second reviewer (TR, PK, LTN). Disagreements that arose during full‐text screening were resolved through consensus discussions.

### 2.5 Data Extraction

Data extraction was completed using forms developed for this review in DistillerSR. All forms were developed a priori, piloted, and modified as required. One reviewer (MS, TR, PK) extracted available data from each of the included studies. A second reviewer (PK, LTN) verified the accuracy and completeness of 30% of the extracted data. Disagreements that arose during data extraction were resolved through consensus discussions. Details extracted from each document included publication details (e.g., author, year), study characteristics (e.g., objective, design), intervention characteristics (e.g., biological fluid sampled, biomarker detected), and outcomes of interest (e.g., clinical outcomes, test characteristics). A full list of items extracted from each document can be found in Table S5.

### 2.6 Data Synthesis and Presentation of Results

The characteristics of included studies are presented in tabular form. Results of this review are presented as descriptive statistics (e.g., counts) of the extracted data using tables and charts and are organized by the secondary research questions addressed. In accordance with the JBI recommendations for the conduct of scoping reviews, quality assessments of included studies were not conducted, and the certainty of the evidence was not assessed [27].

## 3 Results

### 3.1 Study Selection

The MEDLINE and targeted searches returned 6237 results. After the removal of duplicates, 6218 citations underwent title and abstract screening, with 5158 being excluded and 1060 being retrieved for full‐text review. A total of 104 documents (Table 1) were included in this review. A list of all citations excluded during full‐text review and exclusion reasons can be found in the Supporting Information. The study selection process is outlined in Figure 1.

**TABLE 1:** Characteristics of included studies.

Author, year (study name)	Country	Funded	Aim	Target cancer(s)	Biological fluid	Sample size	Population	Main findings
Systematic review without meta‐analysis (*n* = 11)^a^								
Ali et al. (2023) [34]	Australia Canada Czech Republic Germany Italy Netherlands Norway USA	Public	To review the literature on the acceptability of alternative methods for CRC screening as compared to standard care in average‐risk adults.	CRC	Blood	21 studies	Aged 45–86 at average risk for colorectal cancer.	Included 3 RCTs and 1 observational study that compared a blood‐based test to FIT In all 4 studies comparing a blood‐based test to FIT, uptake for the blood based‐test was higher than for FIT
Bittla et al. (2023) [35]	Not reported	None	To evaluate the role of ctDNA in early cancer detection and screening.	Multiple	Blood	12 studies	Articles including individuals aged ≥ 18 published in English after 2013.	No significant utility of ctDNA in detecting early‐stage tumors ≤ 1 cm diameter Sensitivity = 69%–98% Specificity = 99% CSO accuracy 83% for CancerSEEK test
Fagery et al. (2023) [36]	Australia Canada China Colombia Germany Greece Japan Netherlands Singapore Spain United Kingdom USA	Public	To summarize the economic evidence for liquid biopsy tests and studies reporting on challenges of health economic modeling for precision medicine.	Multiple	Blood	24 studies	Studies of individuals at risk of or diagnosed with any type of solid cancer that include a cost‐effectiveness analysis (e.g., CUA, CMA, CCA, CBA) of liquid biopsy tests.	Liquid biopsy technologies were shown to be cost‐effective in 15 studies (75%) 66.7% of included studies evaluated liquid biopsy for screening and early detection 80% of included studies reported cost‐savings or minimal or modest budget impact
LeeVan & Pinsky (2024) [37]	China Denmark Netherlands Portugal Switzerland Sweden USA	Not reported	To perform a systematic review on the performance of cfDNA MCED tests in predicting cancer status.	Multiple	Blood	20 studies	Original articles published between January 2017 and February 2023 that evaluated the performance of an assay that detects 1 or more cancer type(s).	Sensitivity = 46.2% Stage I; 59.5% Stage II; 73.9% Stage III; 83.8% Stage IV Specificity = 95% or higher in 16 (84%) of studies and 99% or higher in 10 (53%) studies AUC = median of 92.6% as reported in 12 studies CSO prediction accuracy for top predictor median 77% accuracy (IQR = 68%–86%)
Patel & Kilgore (2015) [38]	USA	Not reported	To systematically review cost‐effectiveness analyzes of various strategies for CRC screening.	CRC	Stool	17 studies	English‐language studies published between May 2007 and February 2014 that assessed cost‐effectiveness of CRC screening strategies in the USA.	3/17 studies assessed cost‐effectiveness of mt‐sDNA for CRC screening mt‐sDNA had the highest ICER at $34 258 USD/LYG with almost all other strategies (FIT, FOBT, colonoscopy, sigmoidoscopy) having ICERS of below $20 000 USD/LYG
Petit et al. (2019) [39]	Not reported	Public	To review the evidence surrounding the utility of cfDNA and ctDNA for the purposes of CRC screening and diagnosis.	CRC	Blood	69 studies	Articles that assessed the diagnostic accuracy of cfDNA or ctDNA for the purposes of CRC screening.	17/69 studies focused on total cfDNA, 15/69 on ctDNA, and 6 on the DNA integrity matrix 40/69 reviewed methylated cfDNA, with 19 focusing specifically on SEPT9 Methylated cfDNA appeared less specific compared to ctDNA for CRC screening ctDNA were identified as the most promising candidates for blood‐based CRC screening
Niedermaier et al. (2018) [40]	Australia Germany Spain Taiwan USA	Industry	To systematically review the diagnostic accuracy of FIT alone and in combination with a blood markers for CRC.	CRC	Blood	8 studies	Studies reporting the sensitivity and specificity and/or AUC of FIT alone or in combination with blood markers for CRC detection.	3 studies assessed SEPT9 Some blood biomarkers substantially increased sensitivity to CRC when combined with FIT, but this resulted in a loss of specificity For advanced adenomas, there was no increase in sensitivity when blood markers were combined with FIT
Song et al. (2017a) [41]	Not reported	Public	To evaluate the performance of SEPT9 in CRC screening, diagnosis, and therapy by reviewing current evidence.	CRC	Blood	19 studies	Not reported	Combining SEPT9 with FIT and/or CEA enhanced the CRC detection rate Sensitivity = 77.0% for SEPT9; 66.4% for FIT; 41.3% for CEA; 94.3% for SEPT9 + FIT; 86.4% for SEPT9 + CEA; 97.2% for SEPT9 + FIT+CEA
Stürzlinger et al. (2023) [42]	Canada Germany USA	Public	To assess the effectiveness of sDNA testing for CRC detection and to assess the potential organization, social, ethical, and legal issues.	CRC	Stool	8 studies	Studies that included healthy persons aged ≥ 45 who were not at high‐risk for CRC and assessed sDNA tests for CRC screening.	No studies were found that assessed the effects of sDNA testing on overall mortality The sDNA tests assessed in included studies had higher sensitivity for CRC detection and advanced adenomas when compared to FIT or gFOBT, but had lower specificity Test failure rates were higher for sDNA than FIT
Uhe et al. (2021) [43]	China Denmark France Italy Portugal USA	Not reported	To describe the current applications of cfDNA liquid biopsy for gastrointestinal cancer screening.	Multiple	Blood (plasma)	13 studies	Human studies investigating cfDNA liquid biopsy as a screening or diagnosis method for untreated primary gastrointestinal cancers.	Studies investigates CRC (5), pancreatic (2), HCC (3), and multiple cancers (3) Sensitivity = ranged from 71% to 100% across studies Specificity = ranged from 67.4% to 100% across studies CSO accuracy of 61% reported in one study
Zhu et al. (2023) [44]	Not reported	Not reported	To summarize the test characteristics of liquid biopsy test for lung cancer screening.	Lung	Blood Saliva Sputum Urine	13 studies	Studies that analyzed ctDNA/cfDNA in patients with non‐small cell lung cancers with or without comparison to healthy controls.	Sensitivity = ranged from ~22% to 98% across studies Specificity = ranged from 80% to 99.8% across included studies Early‐stage lung cancer screening = ranged from ~20.5% to 91% across studies
Systematic Review With Meta‐Analysis (*n* = 5)^a^								
Hariharan & Jenkins (2020) [45]	China Denmark Germany Hungary South Korea Singapore Taiwan USA	Public	To conduct an updated meta‐analysis to evaluate the range accuracy of the mSEPT9 test at a range of positivity thresholds.	CRC	Blood (plasma, serum)	19 studies	English‐language human subject studies of case–control design that include individuals with a CRC diagnosis prior to or following mSEPT9 test.	Pooled sensitivity = 69% (95% CI: 62–75) Pooled specificity = 92% (95% CI: 89–95) Pooled +LR = 9.1 (95% CI: 6.1–13.8) Pooled −LR = 0.34 (95% CI: 0.27–0.42) Pooled diagnostic OR = 27 (95% CI: 15–48) Average‐risk PPV = 2.6% Average‐risk NPV = 99.9% High‐risk PPV = 9.5% High‐risk NPV = 99.6%
S. Jia et al. (2021) [46]	China USA	Public	To describe the performance of liquid biopsy tests in early cancer detection.	Multiple	Blood (plasma, serum)	17 studies	English‐language, human studies that focus on the early detection of cancer with CTC, cfDNA, ctDNA or other related biomarkers.	Sensitivity = 76% (95% CI: 67–83) Specificity = 92% (95% CI: 86–96) +LR = 9.80 (95% CI: 5.40–17.90) −LR = 0.26 (95% CI: 0.19–0.36) AUC = 0.91 (95% CI: 0.88–0.93) Diagnostic OR = 38 (95% CI: 17–82)
Park et al. (2023) [47]	Not reported	Not reported	To systematically review and statistically examine the applicability and accuracy of a blood based MCED tests to improve cancer detection.	Multiple	Blood	19 studies	Original articles that assessed blood‐based tests for multiple cancer types in asymptomatic individuals.	Sensitivity = 62.3% (95% CI: 51.7–71.9) Sensitivity for early‐stage cancer (I/II) = 50.3% (95% CI: 36.6–63.9) Sensitivity for advanced‐stage cancer (III/IV) = 77.4% (95% CI: 69.7–083.7) Specificity = 97.5% (95% CI: 94.2–99.0) False‐positive rates = 0.025 (95% CI: 0.010–0.058) False negative rates = 0.447 (95% CI: 0.438–0.456)
Song et al. (2017b) [48]	Not reported	Public	To investigate the performance of SEPT9 for CRC screening with a focus on test sensitivity and specificity.	CRC	Blood	25 studies	Studies investigating the performance of the SEPT9 assay.	Sensitivity = 68.0% for SEPT9; 79.0% for FIT; 92.3% for FIT‐sDNA Specificity = 80.0% for SEPT9; 94.0% for FIT; 86.6% for FIT‐sDNA In one study, 97% of participants who refused colonoscopy accepted a non‐invasive screening test, with 83% preferring SEPT9 over FIT
Xu et al. (2023) [49]	Australia USA	Public	To analyze the diagnostic value of methylated BCAT1/IKZF1 in plasma for CRC screening.	CRC	Blood (plasma)	12 studies	Individuals with pathologically confirmed CRC and healthy volunteers.	Sensitivity = 64% (95% CI: 59–69) Specificity = 92% (95% CI: 91–93) +LR = 8.5 (95% CI: 7.1–10.1) −LR = 0.39 (95% CI: 0.33–0.45) Diagnostic OR = 22 (95% CI: 16–30) AUC = 0.92 (95% CI: 0.89–0.94)
Scoping reviews (*n* = 2)^a^								
Davis et al. (2022) [13]	Not reported	Public Industry	To examine the factors that are likely to affect primary care provider and patient use of liquid biopsy testing to detect a single cancer or multiple cancers.	Not applicable	Blood stool	7 studies	Articles related to provider or patient perception of liquid biopsy or MCED testing.	No articles reported on provider perspectives 5 articles reported patients are receptive to liquid biopsy screening methods or MCED
Greene et al. (2024) [50]	Not reported	Public	To evaluate the resources available for patients to learn about the benefits and limitations of MCED testing.	Multiple	Blood	8 studies	English‐language patient education materials that provide information about MCED testing.	Identified 6 websites and 2 printable materials Understandability ranged from 58% to 94% Actionability ranged from 0% to 100%
Randomized controlled trials (*n* = 4)								
Brentnall et al. (2023) [51] (NHS Galleri) ONGOING (protocol)	United Kingdom (England)	Public Industry	To report on the algorithm developed to ensure equity in recruitment for the NHS Galleri trial.	Multiple	Blood	140 000	Aged 50–77 and able to provide informed consent.	Equity in trial recruitment
Coronado et al. (2024) [52]	USA	Industry	To examine whether adherence to CRC screening in a FIT screening program could be improved with a blood‐based test option.	CRC	Blood	2002	Age 45–75 who were provided with a FIT kit, but did not complete it, and were scheduled for follow‐up at one of the study sites.	30.5% of participants in the blood test group completed any CRC screening within 3 months, compared to 13% in the usual care (FIT, colonoscopy) group Follow‐up colonoscopy completion rate of 55% in blood test group compared to 67% in usual care group
Liang et al. (2023) [53]	USA	Public Industry	To compare the uptake of reoffering colonoscopy/FIT to also offering a blood test to individuals who originally declined colonoscopy and FIT.	CRC	Blood	359	Average‐risk individuals aged 50–75 without severe co‐morbidities who were eligible for CRC screening but had previously declined colonoscopy and FIT.	76 (21.2%) were interested in CRC screening with 5.3% preferring colonoscopy, 9.2% FIT, and 12.2% the blood test 9.6% (17/178) of control group completed any form of screening compared to 17.5% (31/181) of intervention group (*p* = 0.035) 9.0% (16/178) of control group and 14.9% (27/181) of intervention group completed full screening (*p* = 0.084)
Neal et al. (2022) [54] (NHS Galleri) ONGOING (protocol)	United Kingdom (England)	Industry	To determine whether an MCED test can reduce the incidence rate of late‐stage cancer.	Multiple	Blood	140 000	Individuals aged 55–77 living in one of 8 participating regions in England.	Cancer stage incidence Sensitivity Specificity PPV NPV Cancer signal origin accuracy Safety Healthcare resource utilization Cancer‐specific mortality Over‐diagnosis
Case–control studies (*n* = 23)								
Abu‐Duhier et al. (2018) [55]	Saudi Arabia Jordan	Public	To determine the clinical significance of cell‐free circulating miRNA‐21 as a biomarker for lung cancer screening.	Lung	Blood (plasma)	160	Histologically confirmed lung cancer patients and healthy controls.	Levels of plasma miRNA‐21 higher among lung cancer patients than healthy controls AUC = 0.8913 (95% CI: 0.9431–0.9431) Specificity = 80% Sensitivity = 80%
Bahar‐Shany et al. (2023) (BEDOCA) [56]	Israel	Public	To describe the performance of a proteomic signature for the identification of high‐grade ovarian cancer using utero‐tubal lavage.	Ovarian	Uterine lavage	500	Clinically healthy BRCA mutation carriers age ≥ 18 with all gynecological organs.	AUC = > 0.97 across models NPV = 100% Only nulliparous individuals reported pain with uterine lavage Adverse events included prolonged pelvic pain (*n* = 2), vaginal discharge (*n* = 1), and vaginal bleeding (*n* = 1)
Bratulic et al. (2022) [57]	Netherlands	Public	To explore whether plasma and urine glycosaminogycans levels differ in healthy controls and 14 cancer types.	Multiple	Blood (plasma) Urine	281	Confirmed cancer diagnosis within 18 months of baseline visit and age, sex, and biochemistry matched controls.	AUC any cancer = 0.65 (95% CI: 0.58–0.72) AUC Stages 0‐II = 0.62 (95% CI: 0.54–0.69) AUC ranges = 0.71 (95% CI: 0.59–0.82) for lung cancer and 0.58 (95% CI: 0.40–0.75) for gynecological cancers
Chen et al. (2021) [58]	China	Public	To evaluate the effect of methylation levels of SEPT9 and SDC2 in leukocytes on the performance of the ColoDefense test for CRC screening.	CRC	Blood (plasma)	213	Aged ≥ 18 with no history of colorectal cancer who had available colonoscopy results.	Levels of SEPT9 and SDC2 in blood leukocytes did not differ between CRC and control groups In plasma samples, SEPT9 and SDC2 methylation levels were higher in Stage I‐III than Stage 0 colorectal cancers Sensitivity (85.9%), specificity (86.8%) and AUC (0.972, 95% CI: 0.949–0.996) of ColoDefence (SEPT9 and SDC2) were higher than either SEPT9 or SDC2 individually
Crook et al. (2022) [59] (TRUEBLOOD)	India	Industry	To describe the validation of a blood test for breast cancer detection using CTCs.	Breast	Blood	10 180	Not reported	Sensitivity = 92.1% (95% CI: 91.1–93.0) Specificity = 100% Accuracy = 99.6% (95% CI: 99.3–99.8)
Einoch Amor et al. (2023) [60]	Israel Latvia	Public Industry	To examine how profiling of liquid biopsies from urine and blood overlap in terms of information on VOCs in multiple cancer types.	Multiple	Blood urine	304	Patients being referred for surgery with a diagnosis of gastric, kidney, or lung cancer and healthy controls.	AUC = 0.91 blood; 0.83 urine; 0.93 combined Sensitivity = 90% blood; 78% urine; 92% combined Specificity = 89% blood; 79% urine; 91% combined Accuracy = 92% blood; 82% urine; 94% combined
Fahrmann et al. (2021) [15]	USA	Public	To assess the lead time trajectory of CA19‐9 using pre‐diagnostic sera.	Pancreatic	Blood (plasma)	1454	Men and women aged 55–74 in the PLCO cohort and age, race, sex, and blood draw date matched controls.	AUC overall = 0.68 (95% CI: 0.64–0.73); sensitivity 16.6% AUC 1–2 years before diagnosis = 0.77% (95% CI: 0.69–0.86); sensitivity 24.3% AUC 0–6 months before diagnosis = 0.87% (95% CI: 0.78–0.96); sensitivity 60%
Gaga et al. (2021) [16]	China Europe Israel	Public Industry	To validate the performance of Lung EpiChek blood test for lung cancer detection.	Lung	Blood	728	Pathologically proven or suspected primary lung cancer and healthy controls aged ≥ 50 who were current or former smokers.	Sensitivity = 87.2% (95% CI: 75.8–90.8) European set, 76.7% (95% CI: 59.1–88.2) Chinese set Specificity = 64.2% (95% CI: 55.6–77.2) European set, 93.3% (95% CI: 68.0–99.9) Chinese set AUC = 0.882 (95% CI: 0.846–0.918) European set, 0.899 (95% CI: 0.809–0.989) Chinese set
Heigh et al. (2014) [61]	USA	Industry Public	To evaluate the performance of mt‐sDNA test for the detection of SSPs or polyps measuring ≥ 1 cm in size.	CRC	Stool	260	Consenting, asymptomatic adults scheduled for CRC screening or polyp surveillance colonoscopy between 2011 and 2012.	Sensitivity = 55% (95% CI: 36–74) Specificity = 91% (95% CI: 87–94) Detection rate was 55% for lesions 1–1.4 cm in size and 53% for those 1.5–3 cm mt‐sDNA was significantly more sensitive for detection of SSP of ≥ 1 cm than FIT at 95% specificity
Y. Kim et al. (2019) [62]	Korea	Industry	To determine whether blood‐based diagnostic techniques are useful for breast cancer screening and clinical practice.	Breast	Blood	1129	Women with breast cancer who agreed to have tissues sampled before treatment and healthy controls who visited the study site for routine checkups.	Sensitivity = 71.6% Specificity = 85.3% PPV = 82.9% NPV = 75.0% AUC = 0.8831 Accuracy = 77% Failed to identify individuals with other types of malignancies and was therefore specific to those with breast cancer only
Li et al. (2019) [63]	China	Public	To use a SFRP2 MethyLight assay to detect methylated SFRP2 in the serum of CRC patients and evaluate the feasibility of using this assay in CRC screening.	CRC	Blood (serum)	124	Individuals with CRC undergoing radical surgery (tissue samples) and individuals with CRC or advanced adenomas who were undergoing colonoscopy at the study site.	SFRP2s methylation levels were higher in 94.1% of CRC tissues compared to paired adjacent paracancer tissues (*p* < 0.001) Sensitivity = 69.4% (95% CI: 52.6–80.1) Specificity = 87.3% (95% CI: 74.9–94.3)
Lima et al. (2023) [64]	Brazil	Public	To investigate the role of cfDNA methylation analysis of SEPT9 and BMP3 genes in the plasma for detecting and CRC lesions.	CRC	Blood	262	Individuals participating in the CRC screening program at the study site and had a positive FOBT test or were diagnosed with CRC.	The CRC group showed the highest cfDNA concentration (10.4 ng/mL) compared to those with adenomas, polyps, and SSLs For SEPT9 gene, a cutoff of 2.5% (AUC = 0.681) resulted in CRC sensitivity of 50% and specificity of 90% For BMP3 gene, a cutoff of 2.3% (AUC = 0.576) resulted in CRC sensitivity of 40% and specificity of 90% Combining SEPT9 and BMP3 resulted in an CRC AUC of 0.845, sensitivity of 80% and specificity of 81%
Luan et al. (2023) [65]	China	Public	To assess the performance of the protein assay OncoSeek for MCED testing.	Multiple	Blood	7565	Individuals with pathologically confirmed cancer prior to treatment (cases) and individuals with no history of cancer (controls).	Sensitivity = 51.7% (95% CI: 49.2–53.9) Specificity = 92.9% (95% CI: 92.3–93.5) Accuracy = 84.3% (95% CI: 83.5–85.0) Accuracy for tissue of origin in true positives = 66.8%
Marcuello et al. (2019) [66]	Spain	Public	To evaluate the diagnostic performance of a 6‐miRNA panel in serum samples of individuals participating in CRC screening.	CRC	Blood (serum)	264	FIT‐positive individuals participating in Barcelona's CRC screening program.	Sensitivity = 81% Specificity = 56% AUC = 0.74 (95% CI: 0.65–0.82) PPV = 58% NPV = 79%
Meng et al. (2021) [67]	China	Industry	To assess the performance of the blood‐based assay, HCCseek for the detection of HCC.	Liver	Blood	323	Treatment naïve individuals with newly diagnosed HCC and healthy controls.	Sensitivity = 75.0% (95% CI: 63.7–84.2) Specificity = 98.0% (95% CI: 95.3–99.3) Accuracy = 92.6% AUC = 0.954
V. Nguyen et al. (2023) [68]	Vietnam	Industry	To expand the SPOT‐MAS approach and evaluate its utility in detecting and locating cancer with a single screening test.	Multiple	Blood	2288	Individuals with localized cancer who were treatment free at the time of blood collection and healthy controls.	Sensitivity = 72.4% AUC = 0.92 (95% CI: 0.90–0.94) Accuracy = 0.70 Sensitivity was lowest for breast cancer (49.3%) and highest for liver (91.1%) Accuracy was lowest for gastric cancer (0.55%) and highest for breast (0.78)
Su et al. (2021) [69]	Taiwan	Public	To investigate the performance of an mt‐sDNA test targeting SDC2 for CRC screening.	CRC	Stool	138	Individuals with diagnosed Stage 0‐IV CRC and healthy controls.	Sensitivity = 77.4% Specificity = 88.2% AUC = 0.872 (95% CI: 0.760–0.891) PPV = 84.2% (95% CI: 72.1–92.5) NPV = 82.7% (95% CI: 72.7–90.2)
Van den Helder et al. (2022) [70]	Netherlands	Public	To determine the clinical performance of methylation analysis in urine for the detection of cervical cancer and high‐grade CIN lesions.	Cervical	Urine	269	Treatment naïve women with histologically confirmed cervical cancer or CIN lesions and healthy controls aged ≥ 18.	AUC = 0.84 (95% CI: 0.78–0.89) Agreement between urine samples and cervical scrapes was 89% (moderate agreement, κ: 0.52, 95% CI: 0.23–0.74) Methylation levels increased with increasing disease severity Detected 95% (109/113) of cervical cancers, 68% (46/64) of CIN3, and 58% (14/24) of CIN2
J. Wang et al. (2020) [71]	China	Public Industry	To evaluate the effectiveness and accuracy of mt‐sDNA testing for the detection of CRC and advanced adenomas.	CRC	Stool	1110	Individuals with definitive or suspected diagnosis of CRC, asymptomatic, with benign lesions, other types of cancer, and healthy controls.	Sensitivity = 83.8% (95% CI: 79.5–87.4) for CRC; 42.1% (95% CI: 26.7–59.1) for advanced adenomas Specificity = 98.0% (95% CI: 99.6–98.9) κ = 0.84 (95% CI: 0.81–0.88) for CRC Detection rate did not differ significantly according to age, tumor location, or TNM stage
Z. Wang et al. (2020) [72]	China	Public	To evaluate the performance of a HK2 cellular marker for screening cells in urine and detecting ETCs in a clinical setting.	Bladder	Urine	384	Individuals with pathologically confirmed urothelial cancer and controls with benign genitourinary disorders, non‐urothelial cancers, and recurrent‐free urothelial cancer.	Sensitivity = 90% (95% CI: 84–94) Specificity = 88% (95% CI: 83–92) PPV = 83% (95% CI: 76–88) NPV = 93% (95% CI: 89–96) AUC = 0.924 (95% CI: 0.893–0.955) ETC counts in urothelial cancer patients (median: 23; IQR: 6–78) were significantly higher than those in controls (median: 0; IQR: 0–0.5; *p* < 0.0001)
Zeng et al. (2023) [73]	China	Public	To explore the value of measuring methylated SDC2 in stool DNA combined with serum tumor markers (CEA) and FIT for detection of colorectal neoplasms.	CRC	Stool	533	First‐time inpatients or outpatients with CRC who had not yet received treatment (drugs or surgery) and healthy controls.	Sensitivity = 75.3% for SDC2; 68.8% for SDC2 + FIT; 70.0% for SDC2 + FIT + serum CEA Specificity = 81.4% for SDC2; 95.0% for SDC2 + FIT; 96.3% for SDC2 + FIT + serum CEA AUC = 0.784 for SDC2; 0.880 for SDC2 + FIT; 0.905 for SDC2 + FIT + serum CEA
Zhang et al. (2022) [74]	Japan	None	To evaluate the performance of a 2‐miRNA model for early‐detection of 12 cancer types.	Multiple	Blood (serum)	7536	Cancer patients and controls.	Sensitivity = ranged from 0% for breast cancer to 99% for lung cancer Specificity = 99.3% across cancer types AUC = ranged from 0.378 for breast cancer to 0.999 for gastric cancer
G. Zhao et al. (2020) [75]	China	Public	To evaluate the performance of a plasma‐based CRC screening assay, SpecColon, in detecting CRC and advanced adenomas.	CRC	Blood (plasma)	318	Individuals with CRC, advanced adenomas, or benign polyps and controls with no evidence of disease.	Sensitivity = 76.2% (95% CI: 67.5–83.3) for CRC; 66.7% (95% CI: 35.4–88.7) for advanced adenomas Specificity = 87.9% (95% CI: 78.9–93.5) for CRC and/or advanced adenomas AUC = 0.856 (95% CI: 0.806–0.905) for CRC
Prospective cohort (*n* = 20)								
Chung et al. (2024) [76]	USA	Industry	To evaluate the performance of a blood based cfDNA test in detecting asymptomatic and early‐stage CRC.	CRC	Blood	22 877	Age 45–85 at average risk of CRC and undergoing routine screening with colonoscopy.	Sensitivity all CRCs = 83.1% (95% CI: 72.2–90.3) Sensitivity Stage I‐III CRCs = 87.5% (95% CI: 75.3–94.1) Specificity = 89.6% (95% CI: 88.0–90.3) PPV = 3.2% CRC, 12.9% pre‐cancerous lesions NPV = 99.9%
Finney Rutten et al. (2017) [77]	USA	Public	To assess whether the early adoption of mt‐sDNA screening differed by demographic characteristics and prior screening behavior.	CRC	Stool	27 147	Adults aged 50–75 living in Olmsted County, Minnesota in 2014.	8.08% of eligible population screened by colonoscopy and 3.59% by mt‐sDNA (*p* < 0.001) Highest rate of mt‐sDNA among 55–59‐year‐olds, who were significantly less likely to use it that 50–54 years olds (aOR = 0.6, 95% CI: 0.5–0.8) 2.82% of males had an mt‐sDNA test and 4.26% of females (aOR = 1.6, 95% CI: 1.3–1.8) 3.34% of those without prior screening ad mt‐sDNA and 16.51% with prior screening (aOR = 0.2, 95% CI: 0.1–0.3) Compared to white people, black people were less likely to use mt‐sDNA compared to colonoscopy (aOR = 0.4, 95% CI: 0.2–0.8)
Gao et al. (2022) [78]	China	Public	To investigate the feasibility of cancer screening with serum tumor markers in the middle‐aged and elderly population.	Multiple	Blood	83 049	Individuals with cancer as confirmed by tumor markers and medical examination.	Serum tumor marker screening identified 89 cancers 35 (39.3%) lung, 5 (5.6%) ovarian, 5 (5.6%) prostate, and 2 (2.2%) other Identified 24 (27%) early‐stage cancer, 25 (28.1%) middle‐stage, and 40 (44.9%) late‐stage
Jin et al. (2022) [79]	China	Public	To estimate the ability of sDNA tests to detect CRC.	CRC	Stool	2842	Individuals over aged ≥ 50 or ≥ 40 with a positive family history of CRC who visited a cancer screening centre or outpatient clinic.	Sensitivity = 90.5% (95% CI: 77.9–96.2) for sDNA Test 1; 92.9% (95% CI: 81.0–97.5) for sDNA Test 2; 81.0% (95% CI: 66.7–90.0) for FIT Specificity = 91.4% (95% CI: 89.8–92.8) for sDNA Test 1; 93.3% (95% CI: 91.9–94.5) for sDNA Test 2; 96.8% (95% CI: 95.7–97.6) for FIT AUC = 0.923 for sDNA Test 1; 0.927 for sDNA Test 2; 0.924 for FIT
Kammesheidt et al. (2018) [80]	USA	Industry	To clinically evaluate ctDNA with a 9 gene, 96 mutation panel among individuals at increased risk of cancer.	Multiple	Blood (plasma)	1059	Individuals aged ≥ 18 without a previous cancer diagnosis but at increased risk of cancer (e.g., tobacco smoking, family history, hereditary cancer gene carrier).	Sensitivity = 66.67% Specificity = 94.87% PPV = 6.9% NPV = 99.8% The false‐positive rate of ctDNA on a 96‐mutation assay was much higher than the true‐positive rate, limiting its usefulness as a cancer screening tool
C. Kim et al. (2021) [81] ONGOING (protocol)	Korea	Industry	To validate the use of the SDC2 methylation detection in sDNA as a screening method for CRC in asymptomatic individuals.	CRC	Stool	1112	Asymptomatic individuals ≥ 60 years of age when they received a colonoscopy and individuals < 60 at high‐risk for CRC.	Sensitivity Specificity
Liu et al. (2020) [82]	China	Public	To evaluate the feasibility of a stool methylated SEPT9 test for CRC screening as compared to plasma SEPT9.	CRC	Blood (plasma) Stool	331	Not reported	Plasma methylated SEPT9 = sensitivity 85.6% specificity 90.1%; AUC 0.885 (95% CI: 0.832–0.938) Stool methylated SEPT9 = sensitivity 97.2%; specificity 48.7%; AUC 0.935 (95% CI: 0.895–0.975) Concordance between plasma and stool SEPT9 was 78.3% Methylated SEPT9 levels lower in normal individuals compared to those with CRC (*p* < 0.001)
Margolis et al. (2022) [83]	USA	Industry	To evaluate the combined performance of the EPI test for predicting GG2 or higher prostate cancer on a first biopsy.	Prostate	Urine	1212	Men aged ≥ 50 years with no prior diagnosis of prostate cancer and PSA levels of 2–10 ng/mL.	AUC = 0.70 (0.67–0.73), significantly higher than the other methods (PSA, PCPT, ERSPC; *p* = < 0.001) EPI cutoff point of 15 = avoids 23% of biopsies and 30% of true‐negative biopsies EPI cutoff point of 20 = avoids 34% of biopsies
Montani et al. (2015) [84] (COSMOS)	Italy	Public	To validate a serum miRNA‐based blood test in individuals at high‐risk for lung cancer.	Lung	Blood	1115	Current or former smokers (within 10 years before study participation) aged ≥ 50.	Accuracy = 74.9% (95% CI: 72.2–77.6) Sensitivity = 77.8% (95% CI: 64.2%–91.4%) Specificity = 74.8% (95% CI: 72.1–77.5) AUC = 0.85 (95% CI: 0.78–0.92)
T. Nguyen et al. (2023) [85] (K‐DETEK)	Vietnam	Industry	To assess the performance and feasibility of SPOT‐MAS in a multi‐centre clinical trial setting.	Multiple	Blood	2795	Age ≥ 40 with no clinical suspicion of or confirmed cancer who presented to one of 13 hospitals in Vietnam for chronic condition follow‐up.	Detection rate: 0.47% (13/2795) identified as having a ctDNA signal True positives = 0.21% (6/2795) False positives = 0.14% (4/2795) PPV = 60% Accuracy = 83.3% Male participants had significantly higher cfDNA levels than females (*p* < 0.0001)
Nimgaonkar et al. (2018) [86]	Not reported	Not reported	Not reported	Multiple	Blood	689	Not reported	Sensitivity = 87% colorectal (77% pre‐cancerous lesions), 100% prostate, 83% breast Specificity: 97.3% colorectal, 100% prostate
Patuleia et al. (2022) [87]	Netherlands	Industry	To evaluate the tolerability of NFA as compared to common breast imaging methods and blood draws.	Breast	Nipple fluid	375	Healthy volunteers aged ≥ 45, patients with untreated primary invasive breast cancer, and individuals at high‐risk of breast cancer.	Overall discomfort = median of 1 (scale of 0–10) Discomfort scores were significantly lower for NFA compared to breast feeding, MRI, and mammography Discomfort was higher than blood collection 98% (311/318) of women would opt for repeated NFA and 97% (308/317) would recommend it to others
Ried et al. (2017) [88]	Australia	Not reported	To compare CTC count to cancer status and risk.	Multiple	Blood	542	Individuals with an increased risk of cancer (e.g., family history, smoking long‐term oral contraceptive use).	CTC were detected in 100% of cancer patients (*n* = 277) and 50% of asymptomatic patients (*n* = 132) In up to 50% of males with normal PSA but detected CTC, PET scans were indicative of prostate cancer Early lung, breast, ovarian, melanoma, and renal cancers were detected in a small number of participants who underwent CTC screening
Schrag et al. (2023) [89] (PATHFINDER)	USA	Industry	To investigate the feasibility of MCED testing in outpatient settings for asymptomatic adults.	Multiple	Blood	6621	Individuals aged ≥ 50 with or without additional cancer risk factors.	Cancer signal detected = 1.4% (92/6621) Median time to diagnostic resolution = 79 days (IQR: 37–219) True positives = 38% (35/92) False positives = 62% (57/92) NNS to detect one cancer = 189 (95% CI: 141–276) Specificity = 99.1% (95% CI: 98.9–99.3) First signal origin correct = 85% (95% CI: 69.9–93.6)
Schrag et al. (2022) [90] (PATHFINDER)	USA	Not reported	To evaluate the clinical feasibility of MCED testing (conference abstract).	Multiple	Blood	6221	Aged ≥ 50 with or without additional cancer risk factors	Specificity = 99.1% PPV = 38.0% (95% CI: 28.8–48.3) NPV = 98.6% (95% CI: 98.3–98.9) CSO prediction accuracy = 97.1% (95% CI: 85.1–99.8) Diagnostic resolution in < 3 months = 73%
Shao et al. (2023) [91] (CCGA)	USA	Industry	To assess the performance of an MCED test (Galleri) in the general population for solid tumors with and without recommended screening and hematological malignancies.	Multiple	Blood (plasma)	2794	Age ≥ 20 either enrolled in the CCGA study or with a high suspicion of cancer or confirmed diagnosis.	Sensitivity solid screened = 34% (95% CI: 31–37); range of 11% for prostate to 82% for CRC Sensitivity solid unscreened = 66% (95% CI: 63–68); range of 0% for thyroid to 93% for liver Hematological malignancy = 55% (95% CI: 49–61); range of 20% myeloid neoplasm to 72% for plasma cell neoplasm Sensitivity generally increased as cancer stage increased
Skates et al. (2017) [92] (ROCA study)	USA	Public	To test a new approach for ovarian cancer screening and compare the proportion of cancers detected at an early stage to historical controls.	Ovarian	Blood	3818	Women at increased familial/genetic risk of ovarian cancer (e.g., BRCA1/2 mutation, multiple first‐degree relatives with ovarian cancer).	Sensitivity = 83% Specificity = 92% PPV = 4.6% (95% CI: 2.1–8.6) Cancer stages detected = 50% (3/6) incident cancers were screen‐detected at an early‐stage compared to 10% of historical BRCA1 cases and 33.5% of historical normal‐risk cases (*p* > 0.10)
L. Song et al. (2020) [93]	China	Public	To investigate the performance of an mSEPT9 assay alone and in combination with serum protein markers for screening for digestive cancers.	Multiple	Blood	2030	Aged ≥ 18 with complete clinicopathological information and confirmed digestive cancer or no evidence of disease.	AUC = 0.86 CRC; 0.76 gastric; 0.69 esophageal; 0.85 liver Sensitivity = 76.6% CRC; 47.7% gastric; 42.6% esophageal; 76.7% liver Specificity = 94.6% CRC; 92.3% gastric; 92.5% esophageal; 87.7% liver
Viste et al. (2020) [94]	Norway	Public	To describe general practitioner's responses to replacing PSA testing with the Stockholm3 and compare PSA and the Stockholm3 with regards to the number of men being referred to prostate biopsy.	Prostate	Blood	4784	Men without known prostate cancer who were referred for prostate cancer testing by their general practitioner.	Only 3% (3/97) of general practitioner clinics had not used the Stockhom3 test 12 months after study start After Stockholm3 test initiation there was a 28% decrease in the number men referred for further workup, with the main effect of the test being a reduced need for biopsies There was a 23%–28% decrease in direct healthcare costs with Stockholm3 as compared to PSA testing
F. Zhao et al. (2023) [95]	China	Public	To evaluate the efficacy of the cfDNA methylation‐based test, ColonSecure, in a population screening setting.	CRC	Blood	3493	Aged 20–90 at high‐risk for CRC who were able and willing to provide blood samples and undergo colonoscopy.	Sensitivity = 86.4% (95% CI: 78.5–91.7) Specificity = 90.7% (95% CI: 89.7–91.6) PPV = 19.3% (95% CI: 16.0–23.1) AUC = 0.956 (95% CI: 0.937–0.975) Detected 34/40 early‐stage CRC (I and II), superior to protein biomarkers
Retrospective cohorts (*n* = 5)								
Engel‐Nitz et al. (2023) [96]	USA	Industry	To assess the healthcare costs associated with different CRC screening modalities.	CRC	Stool	903 831	Men and women aged 50–75 at average risk of CRC with health plan enrollment.	Average yearly costs per person screened ($USD) = 752 for mt‐sDNA, 45 for FIT, 153 for FOBT, and 3029 for colonoscopy Colonoscopy had the highest costs and greatest lost productivity
Hathaway et al. (2021) [97]	USA	Industry	To evaluate mammography uptake and the prosect of adding an MCED test during routine preventive care exams.	Multiple	Blood	8400	Aged 50–64 and enrolled in health insurance with medical and pharmacy benefits for at least 2 continuous years.	Preventive care exams are an opportunity for providers to recommend blood‐based cancer screening tests 63% of all women and 73% of those with breast cancer had a preventive care exam 73% of women with breast cancer had the potential to benefit from an MCED test
Rubio‐Briones et al. (2020) [98]	Spain	Industry	To externally validate a urinary mRNA‐based model for prostate cancer screening and compare it with PSA of ≥ 3 ng/mL and two risk calculators.	Prostate	Urine	492	PSA of 3–10 ng/mL and/or a suspicious digital rectal exam scheduled for initial biopsy without prior MRI.	AUC PSA = 0.591 (95% CI: 0.515–0.668) AUC PSA density = 0.693 (95% CI: 0.626–0.760) AUC PSA 3–10 ng/mL = (95% CI: 0.644–0.764) AUC urinary mRNA = 0.749 (95% CI: 0.690–0.807) AUC ERSPC = 0.736 (95% CI: 0.668–0.803) AUC PBCG = 0.666 (95% CI: 0.594–0.737) Urinary mRNA model avoided 37.2% of excess biopsies
Shore et al. (2019) [99]	USA	Not reported	To evaluate the impact of the SelectMDx assay on prostate biopsy decision making in clinical practice.	Prostate	Urine	418	Men presenting to community urology clinics who received a SelectMDx test while under consideration for a prostate biopsy.	Biopsy rate in SelectMDx (+) group = 60% Biopsy rate in SelectMDx (−) group = 12% > 40% of SelectMDX (+) group had a biopsy within 3 months of testing, compared to < 4% of SelectMDx (−) group SelectMDx did not miss any high‐grade cancers
Voss et al. (2023) [100]	USA	Public Industry	To measure the test‐positivity rate and PPV of an mt‐sDNA test to inform screening for CRC.	CRC	Stool	2758	Individuals with a negative first‐round mt‐sDNA test who underwent one or more additional mt‐sDNA tests.	PPV = 0.25% for CRC; 24% for advanced pre‐cancerous lesions; 67% any colorectal neoplasia NNS to detect 1 CRC or advanced pre‐cancerous lesion = 29 for CRC or advanced pre‐cancerous lesions; 11 for any colorectal neoplasia Positive mt‐sDNA results = 15.3% (422/2758) with 94% undergoing follow‐up colonoscopy
Cross‐sectional (*n* = 12)								
Bessa et al. (2023) [101]	Spain	Public	To determine the performance of a ctDNA based blood assay for the detection of colorectal cancer and advanced pre‐cancerous lesions.	CRC	Blood	640	Men and women aged 50–69 with a positive FIT test who were part of a population‐based colorectal screening program.	Sensitivity = 93% for CRC and 14% for pre‐cancerous lesions Specificity = 87% for non‐advanced lesions and 94% for colonoscopy‐confirmed negative results
Chablani et al. (2017) [102]	USA	None	To provide an assessment of CRC screening preferences (Cologuard, colonoscopy, CTC, FIT) and describe the test attributes that influence these preferences.	CRC	Stool	90	Asymptomatic individuals at average‐risk for CRC aged 50–74 who had undergone screening colonoscopy within the past 5 years.	28/86 (32.6%) participants preferred Cologuard and 58/86 (67.4%) preferred colonoscopy Individuals aged > 60 were more likely to prefer Cologuard compared to those aged ≤ 60 (OR: 3.49, 95% CI: 1.12–10.83) The order of attributes that influenced the choice of Cologuard from highest to lowest were: preparation, procedure, benefits/limitations, physical discomfort, accuracy for CRC detection
Chambers et al. (2023) [103]	USA	Public Industry	To assess the receptivity of primary care providers in utilizing MCED tests in their clinical practice.	Multiple	Blood	351	Staff physicians, residents, fellows, and advanced practice providers	There was strong agreement among practitioners regarding the challenges of utilizing MCEDs (e.g., costs of follow‐up, insurance coverage) Perceived competencies in ordering MCED tests and managing results was strongly correlation to receptivity to using the tests
Gelhorn et al. (2023) [104]	USA	Industry	To quantify preferences for MCED tests using a discrete choice experiment.	Multiple	Blood	1700	Adults aged 50–80 years.	72% of participants preferred MCED tests over current standard tests Preferred screening via blood draw (74%), then imaging (22%), and last invasive procedures (5%)
Imperiale et al. (2021) [105]	USA	Industry	To quantify the specificity of an mt‐sDNA test and determine its sensitivity for CRC and advanced precancerous lesions.	CRC	Stool	816	Aged 45–49 years at average risk for colorectal cancer.	Specificity = 95.2% (95% CI: 93.4–96.6), did not differ by sex or race Sensitivity = 32.7% (95% CI: 19.9–47.5) for advanced precancerous lesions PPV = 30.2% (95% CI: 18.3–44.3) NPV = 95.7% (95% CI: 94.0–97.0) +LR = 6.77 (CI: 4.06–11.28) −LR = 0.71 (CI: 0.58–0.86) AUC = 0.72 (95% CI: 0.64–0.81) No adverse effects of mt‐sDNA testing reported by participants
Myers et al. (2023a) [106]	USA	Public Industry	To assess primary care providers' support for patient participation in research on MCED testing.	Multiple	Blood	27	Primary care providers (physicians and nurse practitioners) from 4 primary care practices in Philadelphia.	93% supported patient participation in MCED research Felt that early cancer detection was important and trials could help develop new screening tests There were concerns around having the time to explain a trial to patients, managing those with positive test results, and managing patients diagnosed with cancer
Myers et al. (2023b) [107]	USA	Public Industry	To assess the receptivity of older adults to MCED testing and identify factors likely to influence test uptake.	Multiple	Blood	159	Primary care patients aged 50–80 with no prior diagnosis of cancer.	79% (125/159) participants reported high levels of interest in having an MCED test Interest was positively associated with the test being recommended by providers, convenience, and effectiveness in finding early‐stage disease There were concerns about insurance coverage of the MCED test
Osborne et al. (2018) [108]	Australia	Public	To determine preferences for CRC screening tests with different sample collections (saliva, blood, stool) and the extent to which specific attributes (costs, performance, convenience) impact this preference.	CRC	Blood Saliva Stool	1282	Women and men aged 50–74 in rural and urban areas representing a range of socioeconomic statuses.	Blood and saliva were equally preferred over stool for the sample type Collection at home or a physician's office were preferred over a pathology centre Test performance was regarded as the most important attribute of a screening test, followed by convenience and sample type and lastly cost
Prince et al. (2017) [109]	USA	Not reported	To evaluate mt‐sDNA uptake in a cohort of screening non‐compliant individuals at average‐risk for CRC.	CRC	Stool	347	Asymptomatic individuals aged ≥ 50 at average‐risk for CRC with no personal history of CRC.	mt‐sDNA test was positive in 14.7% (51/347) of participants Diagnostic colonoscopy was performed in 49 mt‐sDNA‐positive individuals for (96.1% compliance) PPV for advanced colorectal neoplasia = 51.0% PPV for any colorectal neoplasia = 81.6%
Taber et al. (2014) [110]	USA	Not reported	To assess the performance of CRC screening options (colonoscopy, sigmoidoscopy, FOBT, SEPT9) and the perceptions of SEPT9 in a diverse sample.	CRC	Blood	100	Age 50–74 at average risk for CRC.	91% of respondents ranked SEPT9 first or second among test options Preferences differed by race, with 92.9% of unscreened Whites ranking SEPT9 first, compared to 31.3% of unscreened Blacks Common positive aspects of SEPT9 were the convenience, high accuracy, and low cost Negative aspects of SEPT9 were that it cannot detect pre‐cancerous polyps and is not covered by insurance
Yao et al. (2023) [111]	China	Public	To evaluate the efficacy of mt‐sDNA and FIT towards finding more sensitive and specific CRC screening strategies.	CRC	Stool	126	Aged 40–85 with no psychological, social, familial, or geographic limitations that would affect study compliance.	Test positivity rates = 100% (20/20) for mt‐sDNA; 100% (20/20) for FIT quantitative; 90% (18/20) for FIT qualitative AUC = 0.856 for mt‐sDNA; 0.822 for FIT quantitative; 0.642 for FIT qualitative; 0.830 for FIT qualitative + mt‐sDNA; 0.668 for FIT quantitative + mt‐sDNA
Zajac et al. (2016) [112]	Australia	Not reported	The explore the preferences for blood‐based and FIT methods in hypothetical screening scenarios.	CRC	Blood Stool	1561	Aged 45–74.	More participants preferred a blood test (79.6%) compared to stool (20.4%), *p* < 0.001 Adjusted multivariate analysis showed that only those with prior FIT experience had a preference for FIT (OR = 1.53, 95% CI: 1.09–2.16)
Modeling (*n* = 20)								
Aziz et al. (2023) [113]	USA	Public	To estimate the cost‐effectiveness of liquid biopsy for CRC screening compared to standard and no screening.	CRC	Blood Stool	Not reported	Not reported	Cost per participant (USD) = $15 562 for liquid biopsy and $11 583 for sDNA ICER = both liquid biopsy and stool DNA were dominated by other screening methods Cost compared to no screening (USD) = $3555 for liquid biopsy and $2546 for sDNA LYG = 35.58 for liquid biopsy and 35.61 for stool DNA
Barré et al. (2020) [114]	France	Public	To compare the cost‐effectiveness of colorectal screening strategies for average‐risk individuals.	CRC	Blood Stool	5 000 000	General French population aged ≥ 50 with no prior history of screening or family history of CRC, IBD, FAP or Lynch syndrome.	QALY/1000 population = 16.9 for blood‐based test and 14.1 for sDNA compared to no screening Discounted costs (€) = 498.92 for blood‐based and 335.73 for sDNA compared to no screening ICERs (€/QALY) = 154 621 for blood‐based, sDNA was dominated (not as effective and more costly)
Dai et al. (2024) [115]	USA	Industry	To develop a microsimulation model to assess the utility and performance of an MCED test in cancer screening trials.	Multiple	Blood	120 000	General population of USA aged ≥ 50 with no cancer symptoms.	PPV = 48%–61% After 3 annual screening rounds, the proportions of Stage I/II cancers increased by 9%–14% and Stage IV cancers decreased by 37%–46% Mortality reduction of 13%–16%
D'Andrea et al. (2020) [116]	USA	Not reported	To assess the clinical benefits, harms, and burden of testing across strategies for CRC screening using a microsimulation model.	CRC	Blood Stool	80 000	10 000 simulated individuals between the ages of 50–75 for each of the 8 screening strategies	SEPT9 annually averted 37 cases of CRC per 1000 individuals screened and 23 CRC deaths Total colonoscopies highest in colonoscopy screening strategy, followed by SEPT9 screening strategy LYGs = similar across SEPT9, FIT‐DNA, FIT, HS‐gFOBT, CTC, and colonoscopy
Draus et al. (2023) [117]	Sweden	None	To evaluate the cost‐effectiveness of screening for pancreatic cancer using a hypothetical blood‐based biomarker.	Pancreatic	Blood	Not reported	5 hypothetical screening groups: General population aged 50–79 Daily cigarette smokers At risk for familial or hereditary pancreatic cancer New‐onset diabetes Early symptoms suggesting pancreatic cancer	Screening the general population and smokers resulted in high rates of false positives and extra costs ICER (€/QALY) = −17 829–1091 early symptoms group; 65 989‐1 952 920 general population; 10 115–80 267 new‐onset diabetes; 3707–37 881 for familial/hereditary group; 63 679‐2 015 078 smokers
Ebner et al. (2023) [118]	USA	Industry	To determine the benefits, risks, and cost‐effectiveness of stool‐based CRC screening modalities.	CRC	Stool	1000,000	Average risk individuals free of CRC diagnosis at age 40.	LYG/1000 patients = greatest for mt‐sDNA (213) and lowest for FOBT (88) when screening was started at age 45 mt‐sDNA had the greatest reductions in CRC incidence and CRC deaths ICER (USD/QALY) = mt‐sDNA was cost‐effective compared to both FIT (59816) and FOBT (35857)
Fendrick et al. (2023) [119]	USA	Industry	To provide clarity on the potential utility of blood‐based CRC screening.	CRC	Blood Stool	Not reported	Individuals aged 45–75 at average risk of CRC and free of diagnosed CRC at age 40.	With perfect adherence, a blood‐based test produced 19 less LYG than stool‐based strategies At real‐world adherence, blood‐based strategies achieved less LYG compared to mt‐sDNA (287.2 vs. 297.1) and 14% more colonoscopies
Fisher et al. (2021) [120]	USA	Industry	To use the CRC‐AIM model to estimate the cost‐effectiveness of stool‐based screening strategies using real‐world adherence data.	CRC	Stool	1000,000	Average‐risk adults aged 65 who were previously unscreened, free of diagnosed CRC at age 40, and were eligible for screening upon entry into Medicare.	For initial screening with real‐world adherence, mt‐sDNA was cost‐effective compared to both FIT ($62 814 USD/QALY) and FOBT ($39 171 USD/QALY) and yielded better clinical outcomes For initial screening and follow‐up colonoscopy with real‐world adherence, mt‐sDNA was increasingly cost‐effective compared to both FIT ($31 725 USD/QALY) and FOBT ($28 465 USD/QALY) with further increased clinical outcomes
Govers et al. (2018) [121]	USA	Public	To characterize the cost‐effectiveness of SelectMDx in a population of men with elevated PSA.	Prostate	Urine	311 879	Men with elevated PSA or an abnormal digital rectal examination who would undergo ultrasound guided biopsy under current recommendations.	QALYs/person = 10.796 for standard care; 10.841 with SelectMDx Costs (USD/person) = $11 060 for standard care; $9366 for SelectMDx Using SelectMDx prior to biopsy resulted in 14 035 QALYs gained and a savings of $528 323 026 for each yearly cohort SelectMDx dominated standard care across a wide range of sensitivity analyzes
Hao et al. (2022) [122]	Sweden	Public	To assess the cost‐effectiveness of MRI‐based prostate cancer screening using the Stockholm3 (protein biomarkers and PSA) test or PSA alone.	Prostate	Blood (plasma)	Not reported	Men aged 55–69.	The Stockhom3 risk model decreased MRI use by 60% compared to PSA The Stockholm3 strategy was cost‐effective (70% probability of cost‐effectiveness)
Hubbell et al. (2021) [123]	USA	Industry	To estimate the ability of cfDNA‐based MCED test in detecting cancers at an earlier stage and reducing mortality.	Multiple	Blood	Not reported	Individuals aged 50–79 in the SEER program diagnosed with a malignant primary cancer from 2006 to 2015.	MCED test intercepted 485 cancers/year/100000 persons Reduced late‐stage cancer (III/IV) incidence by 78% in those intercepted Could reduce cancer‐mortality by 39%, for an absolute reduction of 104 deaths/100000 intercepted
Hudnut et al. (2023) [124]	Not reported	Industry	To quantify the residual cancer risk where a single confirmatory test does not provide diagnostic resolution after a positive MCED test result.	Multiple	Blood	Not reported	Individuals aged 50–79.	Residual risk ranged from 6% to 20% after the initial confirmatory test was negative (PPV = 40%, 90% accuracy for top predicted CSO) Residual risk ranged from 3% to 18% after second confirmatory test was negative (PPV = 40%, 50% accuracy for top predicted CSO) Risk of false‐positives was 6% and 12% after one and two confirmatory tests, respectively
E. Kim et al. (2023) [125]	United Kingdom USA	Public	To evaluate the potential of risk stratification in increasing the risk–benefit balance of MCED liquid biopsy tests (Galleri Test and DETECT‐A).	Multiple	Blood	249, 037	Unrelated male and female participants in the UK Biobank aged 40–73 of White British ancestry with available genotype data.	PPVs for the Galleri Test and DETECT‐A varied substantially by the level of underlying risk in the population strata PPVs increased as cancer risk scores increased
Ladabaum et al. (2014) [126]	Germany	Industry	To use decision analytic modeling to perform a health economic evaluation of CRC screening in Germany.	CRC	Blood (plasma)	100 000	Aged 50–75 at average risk for CRC.	SEPT9‐based screening was cost‐effective in comparison to no screening but was dominated by other cost‐saving strategies Greatest QALYs gained for FOBT/colonoscopy and lowest for no screening Lowest cost/person was for FIT (1049€) and highest for SEPT9 (2615€) Cost/QALY ranged from 900 to 8000€ compared to no screening
Ladabaum & Mannalithara (2016) [127]	USA	Industry	To explore the cost‐effectiveness of CRC screening with mt‐sDNA compared to FIT and colonoscopy.	CRC	Stool	Not reported	General population aged 50–80.	With optimal adherence, yearly FIT and colonoscopy every 10 years dominated mt‐sDNA every 3 years QALYs = greatest for FIT ICER = FIT was dominant over mt‐sDNA in almost all careening scenarios
Lewis et al. (2024) [128]	Canada	None	To determine whether including an MCED test together with existing screening protocols for breast cancer and CRC would be a cost‐effective strategy from a ministry of health perspective.	Multiple	Blood	100 000	Asymptomatic average‐risk individuals aged 50–75.	MCED screening resulted in more diagnosed cases of each cancer type and at an earlier stage MCED screening was not cost‐effective (ICER of $143 369 CAD per QALY) at a willingness to pay threshold of $100,00 per QALY
Naber et al. (2019) [129]	USA	Public Industry	To assess whether mt‐sDNA is cost‐effective for CRC screening as compared to other standard screening methods.	CRC	Stool	Not reported	Individuals aged 65–75 previously unscreened for CRC.	CRC risk reduction = 46% mt‐sDNA; 50% FIT; 73% colonoscopy CRC death risk reduction = 66% mt‐sDNA; 84% colonoscopy ICER = mt‐sDNA was dominated in all models (less cost‐effective and more costly than other strategies)
Piscitello et al. (2023) [130]	USA	None	To inform the development and evaluation of a blood‐based test for CRC screening by estimating the impact of test performance characteristics on the long‐term benefits and harms.	CRC	Blood	Not reported	Individuals aged 45–85 screened at 3‐year intervals and assumed to be previously unscreened.	A blood test that meets the minimum USA coverage criteria for performance resulted 1576 colonoscopies per 1000 individuals Incidence reduction = 26.7% compared to no screening CRC mortality reduction = 59.2% compared to no screening
Roth et al. (2019) [131]	USA	Industry	To quantify the fiscal and clinical impacts on health plans of increasing CRC screening participation from 65% to 80% in the USA.	CRC	Blood	1000,000	Individuals aged 50–64 at average‐risk of CRC according to USPSTF recommendations.	Number of CRC cases detected over 3 years = 221 with SEPT9; 216 with FIT; 193 with FIT/sDNA; 49 with no screening Cost (3‐year per‐person; USD) = $0.67 for SEPT9; $0.33 for FIT; $0.69 for FIT/sDNA For SEPT9 and FIT, screening and diagnostic costs were similar, but for sDNA, screening costs were higher than diagnostic costs
Sasieni et al. (2023) [132]	United Kingdom (England)	Industry	To develop a model to explore the potential stage‐shift and mortality reduction benefits of MCED‐based screening.	Multiple	Blood	Not reported	Individuals aged 50–80.	Estimated stage‐shifts and cancer‐specific mortality reductions were robust to a range of assumptions Estimated that among 100 000 screened individuals there would be 67 (17%) fewer cancer‐specific deaths 31% of cancers were screen‐detected with MCED compared to 6% of cancer currently being screen‐detected
Qualitative (*n* = 2)								
Marlow et al. (2023) [133] ONGOING (protocol)	United Kingdom	Industry	To assess the psychological impact of having a cancer signal detected on an MCED test.	Multiple	Blood	40	Men and women aged 50–77 with no personal history of invasive cancer who had a cancer signal detected on an MCED test.	Anxiety levels (STAI‐6) Psychological impact of positive MCED test (PCQ) Behavior change after a positive MCED test in relation to routine cancer screening attendance
Schneider et al. (2023) [134]	USA	Industry	To understand the experiences and acceptability of a blood test for CRC screening after previously foregoing FIT.	CRC	Blood	20	Individuals aged 45–75 who were mailed but did not complete a FIT test and primary care and gastroenterology care providers involved in CRC screening.	Individuals were enthusiastic about completing a blood‐test and thought it was more convenient, simple, and had high perceived accuracy Providers were supportive of a blood‐test option if it had adequate performance and if they were provided information for patient education on the pros and cons of the test as compared to other screening options

Abbreviations: €, Euros; +LR, positive likelihood ratio; aOR, adjusted odds ratio; AUC, area under the curve; BCAT1, branched chain amino acid transaminase 1; BEDOCA, biomarkers for early detection of ovarian cancer using uterine lavage; BMP3, bone morphogenetic protein 3; BRCA, breast cancer gene; CA19‐9, carbohydrate antigen 19–9; CBA, cost–benefit analysis; CCA, cost‐consequence analysis; CCGA, circulating cell‐free Genome atlas study; CEA, carcinoembryonic antigen; cfDNA, cell‐free DNA; CI, confidence interval; CIN, cervical intraepithelial neoplasia; CMA, comparative market analysis; COSMOS, continuous observation of smoking subjects; CRC, colorectal cancer; CSO, cancer signal origin; CTC, circulating tumor cells; ctDNA, circulating tumor DNA; CUA, cost‐utility analysis; EPI, exocrine pancreatic insufficiency; ERSPC, European randomized study of screening for prostate cancer; ETC, erythroblast transformation‐specific; FAP, familial adenomatous polyposis; FIT, fecal immunochemical test; FOBT, fecal occult blood test; gFOBT, guaiac fecal occult blood test; GG2, grade group 2; HCC, hepatocellular carcinoma; HK2, hexokinase 2; HS‐gFOBT, high‐sensitivity guaiac fecal occult blood test; IBD, irritable bowel syndrome; ICER, incremental cost‐effectiveness ratio; IKZF1, Ikaros family zinc finger protein 1; IQR, inter‐quartile range; K‐DETEK, early detection of five common cancers using the ctDNA analyzing test study; −LR, negative likelihood ratio; LYG, life‐years gained; MCED, multi‐cancer early detection; miRNA, micro ribonucleic acid; MRI, magnetic resonance imaging; mSEPT9, methylated SEPT9; mt‐sDNA, multi‐target stool DNA; NFA, nipple fluid aspiration; ng/mL, nanograms per milliliter; NHS, National Health Service; NNS, number needed to screen; NPV, negative predictive value; OR, odds ratio; PATHFINDER, assessment of the implementation of an investigational multi‐cancer early detection test into clinical practice study; PBCG, prostate biopsy collaborative group; PCPT, prostate cancer prevention trial; PET, positron emission tomography; PLCO, prostate, lung, colorectal, and ovarian cancer screening trial; PPV, positive predictive value; PSA, prostate‐specific antigen; QALY, quality‐adjusted life year; RCT, randomized controlled trial; ROCA, risk of ovarian cancer algorithm; SDC2, syndecan 2; sDNA, stool DNA; SEER, surveillance, epidemiology, and end results program; SEPT9, septin‐9; SFRP2, secreted frizzled related protein 2; sIG(n)aL, psychological impact of the Galleri test study; SPOT‐MAS, screening for the presence of tumor by methylation and size; SSL, sessile serrated lesion; SSP, sessile serrated polyp; TNM, tumor, lymph nodes, presence of metastasis cancer staging system; TRUEBLOOD, tissue biopsy replacement with unique evaluation of circulating bio‐markers for morphological evaluation and clinically relevant molecular typing of malignancies from BLOOD samples study; USA, United States of America; USD, US dollars; USPSTF, US preventive services task force; VOC, volatile organic compound; Κ, kappa.

^a^ For systematic reviews (with or without meta‐analyzes) and scoping reviews, sample size refers to the number of studies included in the review.

**FIGURE 1:**
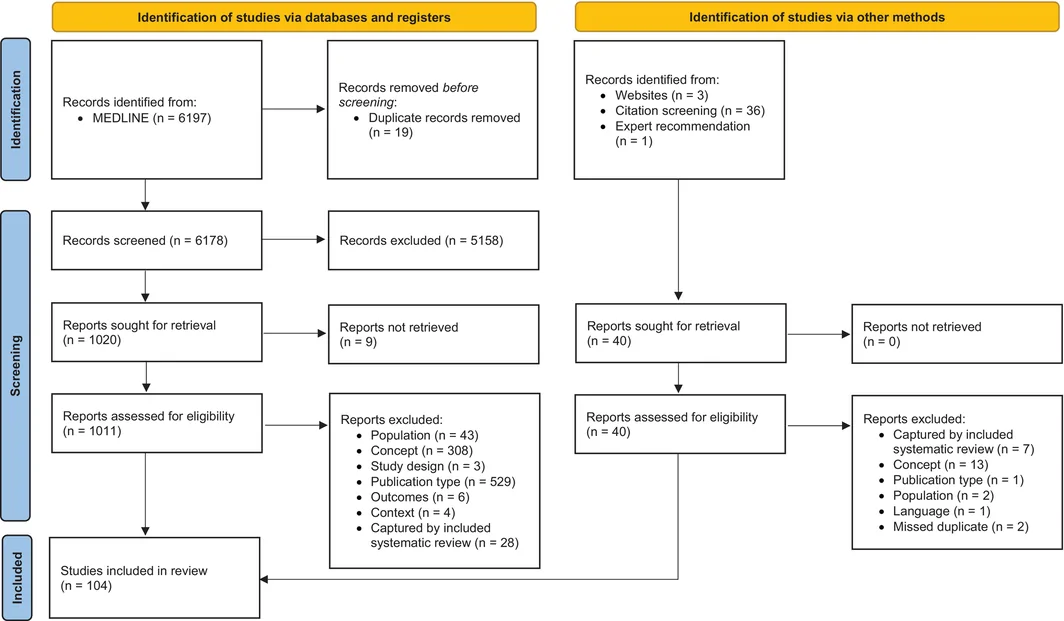
PRISMA flow diagram for study selection. [Colour figure can be viewed at wileyonlinelibrary.com]

### 3.2 Study Characteristics

The sample sizes of included primary research studies ranged from 20 (qualitative study) to 903 831 (retrospective cohort) [15, 16, 51, 52, 53, 54, 55, 56, 57, 58, 59, 60, 61, 62, 63, 64, 65, 66, 67, 68, 69, 70, 71, 72, 73, 74, 75, 76, 77, 78, 79, 80, 81, 82, 83, 84, 85, 86, 87, 88, 89, 90, 91, 92, 93, 94, 95, 96, 97, 98, 99, 100, 101, 102, 103, 104, 105, 106, 107, 108, 109, 110, 111, 112, 133, 134]. The sample sizes of included modeling studies ranged from 80 000 to 5 000 000 simulated participants [113, 132]. The number of studies in included evidence syntheses ranged from 7 to 69 [13, 34, 35, 36, 37, 38, 39, 40, 41, 42, 43, 44, 45, 46, 47, 48, 49, 50]. The included studies were conducted in North America (Canada, United States of America), South America (Brazil, Columbia), Asia (China, India, Israel, Jordan, Korea, Japan, Saudi Arabia, Singapore, Taiwan, Vietnam), Europe (Czech Republic, Denmark, France, Greece, Germany, Hungary, Italy, Latvia, the Netherlands, Norway, Portugal, Spain, Sweden, Switzerland, the United Kingdom), and Australia. Eighty‐five (82%) studies reported receiving funding [13, 15, 16, 34, 36, 39, 40, 41, 42, 45, 46, 48, 49, 50, 51, 52, 53, 54, 55, 56, 57, 58, 59, 60, 61, 62, 63, 64, 65, 66, 67, 68, 69, 70, 71, 72, 73, 75, 76, 77, 78, 79, 80, 81, 82, 83, 84, 85, 87, 89, 91, 92, 93, 94, 95, 96, 97, 98, 100, 101, 103, 104, 105, 106, 107, 108, 111, 113, 114, 115, 118, 119, 120, 121, 122, 123, 124, 125, 126, 127, 129, 131, 132, 133, 134], six (6%) received no funding [35, 74, 102, 117, 128, 130], and 13 (13%) failed to report funding status [37, 38, 43, 44, 47, 86, 88, 90, 99, 109, 110, 112, 116]. Of the 85 funded studies, 41 (48%) received funding from public sources (e.g., universities, governmental) [15, 34, 36, 39, 41, 42, 45, 46, 48, 49, 50, 55, 56, 57, 58, 63, 64, 65, 66, 69, 70, 72, 73, 75, 77, 78, 79, 82, 84, 92, 93, 94, 95, 101, 108, 111, 113, 114, 121, 122, 125], 32 (39%) from private/industry sources [40, 52, 54, 59, 62, 67, 68, 76, 80, 81, 83, 85, 87, 89, 91, 96, 97, 98, 104, 105, 115, 118, 119, 120, 123, 124, 126, 127, 131, 132, 133, 134], and 12 (13%) from a mixture of industry and public sources [13, 16, 51, 53, 60, 61, 71, 100, 103, 106, 107, 129]. The biological fluids investigated included blood (*n* = 77 studies, including plasma and serum samples) [13, 15, 16, 34, 35, 36, 37, 39, 40, 41, 43, 44, 45, 46, 47, 48, 49, 50, 51, 52, 53, 54, 55, 57, 58, 59, 60, 62, 63, 64, 65, 66, 67, 68, 74, 75, 76, 78, 80, 82, 84, 85, 86, 88, 89, 90, 91, 92, 93, 94, 95, 97, 101, 103, 104, 106, 107, 108, 110, 112, 113, 114, 115, 116, 117, 119, 122, 123, 124, 125, 126, 128, 130, 131, 132, 133, 134], stool (*n* = 28) [13, 38, 42, 61, 69, 71, 73, 77, 79, 81, 82, 96, 100, 102, 105, 108, 109, 111, 112, 113, 114, 116, 118, 119, 120, 127, 129, 131], urine (*n* = 9) [44, 57, 60, 70, 72, 83, 98, 99, 121], and saliva (*n* = 2) [44, 108]. Sputum [44], uterine lavage [56], and nipple fluid [87] were each sampled in one study (Figure S1). The characteristics, objectives, and primary results of the included studies are reported in Table 1.

### 3.3 Types of Evidence Available

The studies included in this scoping review consisted of a variety of methodologies such as case–control (*n* = 23) [15, 16, 55, 56, 57, 58, 59, 60, 61, 62, 63, 64, 65, 66, 67, 68, 69, 70, 71, 72, 73, 74, 75], prospective cohort (*n* = 20) [76, 77, 78, 79, 80, 81, 82, 83, 84, 85, 86, 87, 88, 89, 90, 91, 92, 93, 94, 95], modeling (*n* = 20) [113, 114, 115, 116, 117, 118, 119, 120, 121, 122, 123, 124, 125, 126, 127, 128, 129, 130, 131, 132], cross‐sectional (*n* = 12) [101, 102, 103, 104, 105, 106, 107, 108, 109, 110, 111, 112], retrospective cohort (*n* = 5) [96, 97, 98, 99, 100], randomized controlled trial (RCT; *n* = 4) [51, 52, 53, 54], and qualitative designs (*n* = 2) [133, 134] (Figure **2**A). There were 18 evidence syntheses which consisted of systematic reviews without meta‐analyses (*n* = 11) [34, 35, 36, 37, 38, 39, 40, 41, 42, 43, 44], systematic reviews with meta‐analyses (*n* = 5) [45, 46, 47, 48, 49], and scoping reviews (*n* = 2) [13, 50]. It is important to note that modeling studies are primarily based on existing liquid biopsy tests and report outcomes according to pre‐defined assumptions that may not fully reflect real‐world scenarios.

**FIGURE 2:**
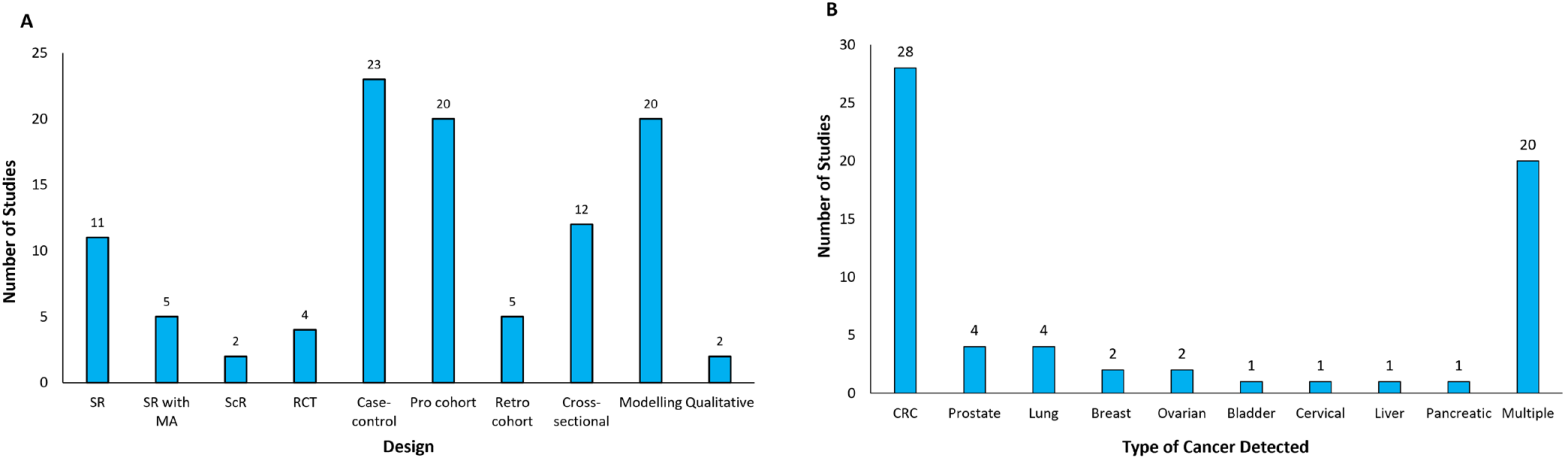
(A) Designs of studies included in the scoping review. (B) Types of cancer detected by liquid biopsy tests in studies included in the scoping review. CRC, colorectal cancer; RCT, randomized controlled trial; ScR, scoping review; SR with MA, systematic review with meta‐analysis; SR, systematic review. [Colour figure can be viewed at wileyonlinelibrary.com]

### 3.4 Biomarkers and Analytical Parameters

A total of 88 (85%) studies reported the types of molecular biomarkers, analytical parameters or both that were investigated (Table 2) [15, 16, 35, 36, 37, 38, 39, 40, 41, 42, 43, 44, 45, 46, 47, 48, 49, 50, 51, 52, 53, 54, 55, 56, 57, 58, 59, 60, 61, 62, 63, 64, 65, 66, 67, 68, 69, 70, 71, 72, 73, 74, 75, 76, 77, 78, 79, 80, 81, 82, 83, 84, 85, 86, 88, 89, 90, 91, 92, 93, 94, 95, 96, 98, 99, 100, 101, 102, 103, 105, 109, 110, 111, 114, 116, 118, 120, 121, 122, 123, 125, 126, 127, 129, 131, 132, 133, 134]. The molecular biomarkers investigated included cfDNA (*n* = 29 studies) [16, 37, 43, 44, 46, 47, 51, 52, 53, 54, 55, 58, 63, 64, 67, 76, 82, 89, 90, 91, 93, 95, 103, 115, 123, 125, 132, 133, 134], multi‐target stool DNA (mt‐sDNA) (*n* = 24) [38, 42, 61, 69, 71, 73, 77, 79, 81, 82, 96, 100, 102, 105, 109, 111, 113, 114, 116, 118, 119, 120, 127, 129], ctDNA (*n* = 17) [35, 36, 39, 41, 44, 45, 46, 48, 50, 59, 68, 75, 80, 85, 101, 110, 116], specific genes (*n* = 10; e.g., SEPT9, BCAT1/IK2FI) [40, 49, 70, 83, 99, 114, 116, 121, 126, 131], specific proteins (*n* = 8) [15, 56, 62, 65, 78, 92, 94, 122], micro ribonucleic acid (miRNA) (*n* = 4) [44, 66, 74, 84], and CTC (*n* = 3) [46, 86, 88]. An additional four studies examined less commonly used biomarkers such as glycosaminoglycans [57], messenger RNA (mRNA) [98], Hexokinase 2 [72], and volatile organic compounds [60]. The analytical parameters investigated in these studies included quantification of specific biomarkers (*n* = 30) [15, 40, 44, 50, 55, 56, 57, 59, 60, 62, 65, 66, 72, 74, 78, 83, 84, 86, 88, 92, 94, 98, 99, 101, 105, 109, 115, 121, 122, 123], methylation of specific biomarkers (*n* = 32) [16, 36, 37, 39, 40, 41, 43, 45, 48, 49, 53, 58, 61, 63, 64, 69, 70, 71, 73, 75, 77, 79, 82, 93, 100, 102, 110, 111, 114, 116, 126, 131], DNA methylation patterns (*n* = 13) [37, 42, 51, 54, 68, 81, 85, 89, 90, 91, 95, 132, 133], and mutation of specific genes (*n* = 13) [37, 43, 52, 67, 76, 79, 80, 96, 100, 101, 111, 123, 134].

**TABLE 2:** Biomarkers and analytical parameters investigated in included studies.

Analytical parameter^a^	Biomarker detected^a^	Test name	Reference(s)		
			Primary studies	Reviews	Modeling
Quantification of specific biomarker	cfDNA	TaqMan	[55]		
		Not applicable		[44]	[115, 123]
	ctDNA	Not applicable	[59, 101]	[44, 50]	
	CTC	CellMax	[86]		
		Not applicable	[88]		
	mt‐sDNA	Not applicable	[105, 109]		
	miRNA	Not applicable	[66, 74, 84]	[44]	
	Proteins	OncoSeek	[65]		
		Stockholm3	[94]		[122]
		Not applicable	[15, 56, 62, 78, 92]		
	Specific genes	ExoDX prostate	[83]		
		SelectMDX	[99]		[121]
	Other^b^	Not applicable	[57, 60, 72, 98]	[40]	
Methylation of specific biomarker	cfDNA	ColoDefense	[58]		
		Lung EpiCheck	[16]		
		MethyLight	[63]		
		Not applicable	[53, 64, 82, 93]	[37, 43]	
	ctDNA	SpecColon	[75]		
		Not applicable	[110]	[36, 39, 41, 45, 48]	[116]
	mt‐sDNA	ColoGuard	[77, 102]		[131]
		Not applicable	[61, 69, 71, 73, 79, 82, 100, 111]		[114]
	Specific genes	Not applicable	[70]	[40, 49]	[114, 116, 126, 131]
DNA methylation patterns	cfDNA	ColonSecure	[95]		
		Galleri	[51, 54, 89, 90, 91, 133]		[132]
		Not applicable		[37]	
	ctDNA	SPOT‐MAS	[68, 85]		
	mt‐sDNA	EarlyTect Colon cancer	[81]		
		Not applicable		[42]	
Mutation of specific gene(s)	cfDNA	HCCseek	[67]		
		Shield	[52, 76, 134]		
		Not applicable		[37, 43]	[123]
	ctDNA	Not applicable	[80, 101]		
	mt‐sDNA	Not applicable	[79, 96, 100, 111]		
Not reported	cfDNA	Not applicable	[103]	[47, 48]	[125]
	ctDNA	Not applicable		[35, 46]	
	mt‐SDNA	Not applicable		[38]	[113, 116, 118, 119, 120, 127, 129]
	CTC	Not applicable		[46]	
	Other^b^	Not applicable		[46]	
	Not reported	Not applicable	[87, 97, 104, 106, 107, 108, 112]	[13, 34]	[113, 115, 117, 119, 124, 128, 130]

Abbreviations: cfDNA, cell‐free DNA; ctDNA, circulating tumor DNA; DNA, deoxyribonucleic acid; miRNA, micro ribonucleic acid; mt‐sDNA, multi‐target stool DNA.

^a^ Note that more than one biomarker or analytical parameter could be reported per study.

^b^ Included circulating nucleosomes, glycosaminoglycans, Hexokinase 2, messenger RNA, volatile organic compounds or multiple types of biomarkers.

### 3.5 Cancer Types Detected

The use of liquid biopsy tests for the purposes of cancer and/or pre‐cancer detection was assessed in 64 (62%) of the included studies (Figure **2**B) [15, 16, 35, 37, 39, 40, 41, 42, 43, 44, 45, 46, 47, 48, 49, 52, 53, 54, 55, 56, 57, 58, 59, 60, 61, 62, 63, 64, 65, 66, 67, 68, 69, 70, 71, 72, 73, 74, 75, 76, 78, 79, 80, 81, 82, 83, 84, 85, 86, 88, 89, 90, 91, 92, 93, 94, 95, 98, 99, 100, 101, 105, 109, 111]. The remaining studies did not examine cancer detection or screening specifically, and instead focused on other aspects of the tests, such as cost‐effectiveness, participant preferences, and projected outcomes (e.g., modeled cancer‐specific mortality reduction). The most frequently detected cancers in these studies were CRC (*n* = 28) [39, 40, 41, 42, 45, 48, 49, 52, 53, 58, 61, 63, 64, 66, 69, 71, 73, 75, 76, 79, 81, 82, 95, 100, 101, 105, 109, 111], prostate (*n* = 4) [83, 94, 98, 99], and lung (*n* = 4) [16, 44, 55, 84]. Included studies also reported on tests that detected breast [59, 62], ovarian [56, 92], bladder [72], cervical [70], liver [67], and pancreatic cancer [15]. Twenty studies reported on MCED tests that detected multiple types of cancer [35, 37, 43, 46, 47, 54, 57, 60, 65, 68, 74, 78, 80, 85, 86, 88, 89, 90, 91, 93].

### 3.6 Outcomes Reported and Key Findings

Most studies (*n* = 61, 58%) reported on outcomes related to test characteristics such as sensitivity, specificity, and negative and positive predictive values [15, 16, 35, 37, 39, 40, 41, 42, 43, 44, 45, 46, 47, 48, 49, 55, 56, 57, 58, 59, 60, 61, 62, 63, 64, 65, 66, 67, 68, 69, 70, 71, 72, 73, 74, 75, 76, 79, 80, 82, 83, 84, 85, 86, 89, 90, 91, 92, 93, 95, 98, 100, 101, 105, 109, 111, 115, 117, 123, 124, 125]. Clinical outcomes (e.g., expected cancer‐specific mortality, cancer stage, incidence) were reported in 23 studies [78, 89, 90, 92, 94, 100, 101, 109, 113, 115, 116, 118, 119, 120, 122, 123, 126, 127, 128, 129, 130, 131, 132], cost‐effectiveness outcomes in 17 [36, 38, 94, 96, 113, 114, 117, 118, 119, 120, 121, 122, 126, 127, 128, 129, 131], and implementation outcomes (e.g., test uptake, perceptions) in 23 [13, 34, 42, 48, 50, 51, 52, 53, 56, 77, 87, 94, 97, 102, 103, 104, 106, 107, 108, 109, 110, 112, 134]. A summary of the outcomes reported in studies is presented in Table 3 and results are briefly summarized below. Main findings from included studies are presented in Table 1.

**TABLE 3:** Outcomes reported in studies on liquid biopsy for cancer screening.

Outcome	Total number of studies^a^	Primary studies^b^	Reviews^c^	Modeling studies
Clinical outcomes	23	8	0	15
Expected cancer‐specific mortality^d^	12	0	0	12
Incidence of cancer	11	1	0	10
Cancer stage	8	4	0	4
Extent of diagnostic testing	3	2	0	1
Time to resolution	2	2	0	0
NNS	2	2	0	0
Test characteristics	61	43	13	5
Sensitivity	46	33	13	0
Specificity	42	31	11	0
AUC	34	28	5	1
PPV	22	17	1	4
NPV	14	13	1	0
False positive	7	3	1	3
Accuracy	5	5	0	0
+ LR	4	1	3	0
‐LR	4	1	3	0
Diagnostic OR	3	0	3	0
CSO prediction accuracy	5	2	3	0
False negative	2	1	1	0
Cost‐effectiveness	17	2	2	13
ICER	12	0	2	10
QALY	9	0	0	9
Total costs	9	2	1	6
LYG	5	0	0	5
Implementation	23	18	5	0
Test preferences	9	7	2	0
Perceptions of test	7	5	2	0
Test uptake	7	5	2	0
Receptivity to test	5	5	0	0
Implementation considerations	2	1	1	0
Health equity considerations	1	1	0	0
Other	24^e^	18	1	5

Abbreviations: +LR, positive likelihood ratio; AUC, area under the curve; CSO, cancer signal origin; ICER, incremental cost‐effectiveness ratio; −LR, negative likelihood ratio; LYG, life‐years gained; NNS, number needed to screen; NPV, negative predictive value; OR, odds ratio; PPV, positive predictive value; QALYs, quality‐adjusted life‐years.

^a^ Studies included in this review (*n* = 104) could have reported more than one outcome.

^b^ Primary studies include randomized controlled trials, prospective cohorts, retrospective cohort studies, case–control studies, and cross‐sectional studies. Some primary studies reporting on the outcomes of interest were excluded from this scoping review due to their inclusion by one or more of the included systematic or scoping reviews. Reported counts for primary studies reflect only the primary studies included in this scoping review and may therefore underestimate the total number of available primary studies reporting on that outcome.

^c^ Reviews includes systematic reviews (with and without meta‐analyzes) and scoping reviews.

^d^ Cancer‐specific mortality is reported here as the expected cancer‐specific mortality because the evidence for this outcome came from modeling studies only and is therefore based on expected rather than observed events.

^e^ Other outcomes included outcomes such as cancer risk, levels of specific biomarkers, differences in methylation levels, and the number of blood tests administered.

Few studies examined the impact of liquid biopsy and MCED tests on clinical outcomes. Evidence from empirical primary studies on the impact of these tests on clinical outcomes (e.g., randomized controlled trials with the outcome of cancer‐specific mortality) was not identified. However, modeling studies reported that MCED tests for cancer screening could lead to expected reductions in cancer‐specific mortality (13%–39% reductions across studies) and late‐stage cancers [115, 116, 118, 120, 122, 123, 128, 132]. Expected reductions in cancer‐specific mortality for liquid biopsy tests of single cancers were reported only for CRC, with studies reporting that mt‐sDNA or blood‐based tests could lead to reductions in both CRC incidence (27% reduction) and specific mortality (59%–66% reduction) [116, 118, 120, 126]. Test characteristics were the most reported outcomes across studies. Across included primary studies, the sensitivity of single cancer tests ranged from 33%–97% for CRC [58, 61, 63, 64, 66, 69, 71, 73, 75, 76, 79, 82, 95, 101, 105], 78%–87% for lung cancer [16, 55, 84], and 72%–92% for breast cancer [59, 62]. In the studies examining single cancer tests for liver, ovarian, and bladder cancer, sensitivities were 75%, 83%, and 90%, respectively [67, 72, 92]. Sensitivities for MCED tests ranged from 0% for thyroid cancer to 100% for prostate cancer [65, 68, 74, 80, 86, 91, 93]. The specificity of single cancer tests ranged from 56%–98% for CRC [58, 61, 63, 64, 66, 69, 71, 73, 75, 76, 79, 82, 95, 101, 105], 64%–80% for lung cancer [16, 55, 84], and 85%–100% for breast cancer [59, 62]. Studies examining single cancer tests for liver, ovarian, and bladder cancer reported specificities of 98%, 92%, and 88%, respectively [67, 72, 92]. Specificities for MCED tests ranged from 79%–100% [60, 65, 74, 80, 86, 89, 90, 93].

The limited evidence identified on the cost‐effectiveness of MCED was mixed, with one review stating that these technologies were cost‐effective in 75% of studies [36] and one modeling study reporting that they were not cost‐effective at a willingness to pay threshold of $100 000 per quality‐adjusted life year [128]. Most evidence on the cost‐effectiveness of liquid biopsy tests for single cancer screening pertained to CRC. Studies showed mixed results, with one reporting that mt‐sDNA is cost‐effective for CRC screening compared to fecal immunochemical tests (FIT) and fecal occult blood tests (FOBT) [120] and others reporting that it is only cost‐effective compared to no screening [113, 126].

In terms of the acceptability of liquid biopsy and MCED tests for cancer screening, participants reported being generally receptive, with many preferring these tests over standard care (e.g., colonoscopy, FIT, mammography) [53, 104, 108, 110]. Screening via blood draw was reported to be preferred over more invasive procedures and imaging [104, 112, 134]. In several studies, however, physicians and participants frequently reported concerns about the current lack of insurance coverage for the tests [103, 107, 110].

### 3.7 In‐Progress Clinical Trials and Studies

The MEDLINE and targeted website searches identified four ongoing studies. Three of these studies are part of the National Health Service Galleri Trial [51, 54, 133], with one being an independent study on the validation of syndecan 2 for CRC screening [81]. The search of ClinicalTrials.gov identified 21 ongoing clinical trials that aim to assess liquid biopsy tests for the purpose of cancer screening in asymptomatic individuals [135, 136, 137, 138, 139, 140, 141, 142, 143, 144, 145, 146, 147, 148, 149, 150, 151, 152, 153, 154, 155] (Table S6). Five of these trials are interventional, with two RCTs [150, 152], two single group allocations [154, 155], and one parallel allocation design [140]. There are 16 observational studies, with 10 prospective cohorts [135, 136, 137, 139, 143, 144, 147, 148, 151, 153], five case–control studies [141, 142, 145, 146, 149], and one retrospective cohort [138]. The estimated sample sizes in the studies range from 90 to 150 000. The primary outcome measures are sensitivity and/or specificity in 15 (71%) of the ongoing studies.

## 4 Discussion

This scoping review identified a large body of evidence on liquid biopsy tests for the purposes of cancer screening in asymptomatic individuals. These tests have been investigated for their utility in screening for a range of cancer types, using a wide variety of different biological fluids, biomarkers, and analytical parameters. This review also identified several ongoing clinical trials on liquid biopsy for the purposes of cancer screening. As these studies are completed, they will continue to add to the evidence base.

Test characteristics (e.g., sensitivity, specificity) were the most common outcomes reported across studies, with very limited evidence on clinical outcomes (e.g., incidence, cancer‐specific mortality, harms). Fewer of the included studies reported on cost‐effectiveness and implementation (e.g., uptake, individuals' test preferences) of liquid biopsy tests. While it is important for the performance and costs of these tests to be established, evidence as to whether the potential benefits (i.e., reduction in cancer‐specific mortality) outweigh the potential harms is needed. It is important to note that none of the included studies prospectively examined cancer‐specific mortality. These findings are consistent with those from the systematic review conducted by Kahwati et al. [25] on blood‐based MCED tests for cancer screening. While some evidence on expected cancer‐specific mortality from modeling studies was identified, modeling studies are limited as they make certain assumptions, some of which would not occur in the real world (e.g., perfect adherence). A lack of prospective evidence on cancer‐specific mortality, arguably the most important outcome in cancer screening, currently precludes the use of liquid biopsy and MCED tests in organized screening programs. There is ongoing debate however, on the provisional use of liquid biopsy and MCED tests before traditional RCTs are completed, and whether cancer‐specific mortality should continue to serve as the primary endpoint in cancer screening studies [156]. There was also a paucity of evidence surrounding the logistics of implementing liquid biopsy tests for cancer screening, including optimal screening intervals. Further studies on how these tests perform in real‐world settings is therefore required but are likely to be highly resource intensive.

Although it has been argued that liquid biopsy tests may address equity gaps faced by current cancer screening programs, there is currently insufficient evidence to determine whether they can achieve this. This review identified only one study that reported outcomes related to health equity in liquid biopsy tests, highlighting a significant gap in the research. This is an important area for future investigation given the potential for alternative non‐invasive and easy‐to‐administer screening tests to improve access and uptake of cancer screening for various underserved populations.

Along with the potential benefits of these emerging technologies, the potential harms of screening tests must always be assessed and considered in balance [14]. Similar to the findings from Kahwati et al. [25] we found that the potential harms associated with the use of liquid biopsy, both to individuals and the healthcare system, have not been thoroughly evaluated. We identified a few studies which reported on false positive and false negative rates [47, 80, 85, 89, 111, 117, 123, 124] but evidence on other potential harms is lacking. Beyond false‐positive and false‐negative results, other potential harms include the identification of harmless non‐cancer related incidental findings or the identification of clinically insignificant cancers that may result in both overdiagnosis and overtreatment. Individuals may also experience radiation exposure or complications resulting from unnecessary follow‐up procedures and treatment. The use of MCED tests are likely to result in more harms than single‐cancer screening tests as they require extensive work up following a positive test to locate the potential cancer [17], frequently triggering a diagnostic cascade that includes procedures which are invasive or financially burdensome (e.g., full‐body imaging). Because these tests are so new, there is not yet consensus on the appropriate diagnostic pathways for individuals with a positive test, which is one of the principles of disease screening [157]. If cancer cannot be found in people who screen positive on an MCED test, the test is considered a false positive, and health care providers and screening participants are left with considerable uncertainty about the next steps (i.e., is more diagnostic testing required and when?) and likely, considerable anxiety [158, 159]. Finally, the impact on healthcare utilization and costs, including both health system costs and participants' out‐of‐pocket costs, resulting from using these tests for cancer screening must be considered [14].

The most common cancer type investigated in the included studies was CRC. Participation in organized CRC screening programs is sub‐optimal, remaining below recommended targets both in Canada and internationally [160, 161, 162]. Sub‐optimal participation in stool‐based CRC screening programs (e.g., fecal occult blood tests or fecal immunochemical tests) may be partly due to individuals' doubt in their ability to properly complete at‐home stool collection, disgust, and logistical issues with stool collection [163]. There are also barriers to participation for endoscopic CRC screening methods, including fear, apprehension about bowel preparation, and lack of sedation (e.g., flexible sigmoidoscopy) [164]. Blood‐based tests could potentially increase screening uptake and overcome the barriers of stool‐based and endoscopy screening methods as they require only a simple blood draw for completion. Studies also assessed the utility of liquid biopsy tests in screening for cancers that are not currently screened for at the population level (e.g., liver, ovarian, pancreatic, and bladder cancer). While some of the studies included in this review found that liquid biopsy tests can be used to identify cancers that do not have established screening tests or programs, necessary evidence on the impact on cancer‐specific mortality and other clinically important outcomes is lacking.

Over half (52%) of the funded studies included in this scoping review received industry funding. Industry funding is common in cancer research as they can better mobilize expertise, resources, and infrastructure for trials than limited public finding can [165]. The funding source should be considered when interpreting study results. Industry sponsorship can lead to funding bias in which the study results tend to favor the study sponsors [166]. Further, corporate interests can drive research agendas away from public health priorities [167]. The use of publicly available study protocols, data analysis plans, and data, as well as the conduct of statistical analyses by independent analysts may help to improve transparency and alignment of industry and public health priorities [166].

To our knowledge, this is the first review to provide a comprehensive overview of the evidence surrounding the use of all liquid biopsy and MCED tests for cancer screening. While other reviews exist, they commonly focus on a single cancer, test, or biomarker [22, 23, 25]. Additionally, previous reviews of MCED tests have focused primarily on test characteristics [35, 37]. We did not restrict included studies according to cancer type, biomarkers used, or the outcomes examined. As a result, this scoping review provides a fulsome map of the evidence that can be used to identify gaps and areas of importance for future evidence syntheses and primary research.

### 4.1 Strengths and Limitations

Our review has several strengths. This review used a search strategy that was developed with an experienced medical librarian. It was also conducted according to a protocol made available a priori via Open Science Framework. Furthermore, the study was reported according to the PRISMA Checklist version for scoping reviews, ensuring standardized and transparent reporting [29]. A reliable and automated software platform (DistillerSR) was used to manage the review process, potentially minimizing human error through the use of standardized processes for citation screening and data extraction [168].

There are some limitations to our review. First, this scoping review used streamlined methods and accelerated processes to identify and summarize evidence in an efficient timeframe [169]. A single database (i.e., MEDLINE) was searched for this review, which could have resulted in a failure to identify some relevant studies. However, previous research has found that searching one database can be sufficient and including multiple databases may have minimal effect on the review outcomes [170]. Additionally, a number of supplemental sources (e.g., gray literature, reference lists) were searched, mitigating the potential risk of publication bias and increasing the comprehensiveness of the search [171]. Second, the literature searches were limited to studies available in English, resulting in an unknown number of non‐English studies being omitted from search results. However, prior evidence has suggested that the exclusion of non‐English language documents from evidence syntheses has minimal impact on overall review conclusions [172]. Lastly, citation screening and data extraction for this review were conducted by a single reviewer. However, second reviewer verification was completed on 30% of citation screening decisions and data extraction, which can improve the completeness and accuracy of the data compared to a single reviewer [169].

### 4.2 Future Directions

This study has identified gaps in the current literature that can serve as the basis for future work. Future research should focus on the impacts of liquid biopsy and MCED tests on cancer‐specific mortality, especially in comparison to existing screening tests. Evidence for these tests in reducing cancer‐specific mortality is required before they can be integrated into organized screening programs. Cancer‐specific mortality as the primary endpoint for cancer screening trials is well supported and suggestions have been made for how this outcome could be integrated into trials of liquid biopsy and MCED, as evidenced by phase four and five clinical trials currently being conducted in the Early Detection Research Network [156]. Future research should also focus on how these tests can be integrated into existing screening programs and developing guidelines for use, including optimal testing intervals and follow‐up of abnormal screening results. Should these tests be integrated into routine clinical practice, robust processes for monitoring, evaluating, and reporting on the impact of these tests should be followed. Furthermore, implementation research and health equity analyses will be critical as liquid biopsy and MCED technologies continue to advance and gain wider spread use.

### 4.3 Conclusion

This scoping review represents the first comprehensive overview of the evidence on the use of liquid biopsy and MCED tests for the purposes of cancer screening in asymptomatic adults. This review shows that these tests have potential utility in detecting a range of cancer types, using a variety of biomarkers. The review also demonstrated, however, that there are critical gaps in knowledge about the outcomes of these tests when used for cancer screening that must be filled before these technologies can be considered for implementation in population‐level or risk‐based cancer screening programs. Specifically, the scoping review revealed that additional investigations into the impacts on cancer‐specific mortality, harms, barriers, and system‐level impacts need to be conducted.

## Author Contributions

**Tessa Robinson:** data curation, formal analysis, investigation, visualization, writing – original draft, writing – review and editing. **Marian Saab:** data curation, formal analysis, investigation, methodology, visualization, writing – original draft, writing – review and editing. **Lily Thao Nguyen:** formal analysis, investigation, writing – review and editing. **Pooja Kochar:** formal analysis, investigation, writing – review and editing. **Olivia Meggetto:** conceptualization, data curation, formal analysis, investigation, methodology, project administration, supervision, validation, visualization, writing – review and editing. **Jill Tinmouth:** conceptualization, methodology, supervision, writing – review and editing. **Meghan J. Walker:** conceptualization, methodology, project administration, resources, supervision, writing – review and editing.

## Funding

This study was conducted with the support of Ontario Health (Cancer Care Ontario) through in‐kind contributions. The opinions, results, view, and conclusions reported in this publication are those of the authors and do not necessarily reflect those of Ontario Health (Cancer Care Ontario). No endorsement by Ontario Health (Cancer Care Ontario) is intended or should be inferred.

## Conflicts of Interest

The authors declare no conflicts of interest.

## Supporting information


**Table S1:** Preferred reporting items for systematic reviews and meta‐analyzes extension for scoping eeviews (PRISMA‐ScR) checklist.
**Table S2:** Full inclusion and exclusion criteria.
**Table S3:** MEDLINE search strategy.
**Table S4:** List of gray literature sources.
**Table S5:** Data extraction items.
**Figure S1:** Types of biological fluid sampled in included studies.
**Table S6:** Ongoing clinical trials examining the use of liquid biopsy and MCED tests for the purposes of cancer screening in asymptomatic populations.

## Data Availability

The data underlying this article are available in the article and in its Supporting Information. These data were extracted directly from the included studies in this review.
