# Supplementary material for: Liquid Biopsy and Multi‐Cancer Early Detection Tests for Cancer Screening: A Scoping Review
Source: Clin Genet. 2026 Sep 25;110(6):647–85. doi: 10.1111/cge.70249 (PMC13728974; doi:10.1111/cge.70249)
Supplement: Supplementary file 1 [file CGE-110-647-s001.docx]

**The use of liquid biopsy and multi-cancer early detection tests for cancer screening: A rapid scoping review.**

**Online Supplementary Materials**

Supplementary Table S1: Preferred Reporting Items for Systematic Reviews and Meta-Analyses Extension for Scoping Reviews (PRISMA-ScR) Checklist

Supplementary Table S2: Full inclusion and exclusion criteria

Supplementary Table S3: MEDLINE search strategy

Supplementary Table S4: List of grey literature sources

Supplementary Table S5: Data extraction items

Supplementary Methods: Full text exclusions and reasoning

Supplementary Figure S1: Types of biological fluid sampled in included studies

Supplementary Table S6: Ongoing clinical trials examining the use of liquid biopsy and MCED tests for the purposes of cancer screening in asymptomatic populations

**Supplementary Table S1: Preferred Reporting Items for Systematic Reviews and Meta-Analyses Extension for Scoping Reviews (PRISMA-ScR) Checklist**

| **Section** | **Item** | **Checklist Item** | **Location Reported** |
| --- | --- | --- | --- |
| **Title** | | | |
| Title | 1 | Identify the report as a scoping review. | Title page  (pg. 1) |
| **Abstract** | | | |
| Structured summary | 2 | Provide a structured summary that includes (as applicable): background, objectives, eligibility criteria, sources of evidence, charting methods, results, and conclusions that relate to the review questions and objectives. | Abstract  (pg. 2) |
| **Introduction** | | | |
| Rationale | 3 | Describe the rational for the review in the context of what is already known. Explain why the review questions/objectives lend themselves to a scoping review approach. | Introduction  (pg. 3-4) |
| Objectives | 4 | Provide an explicit statement of the question and objectives being addressed with reference to their key elements (e.g., population or participants, concepts, and context) or other relevant key elements used to conceptualize the review question and/or objectives. | Research questions  (pg. 4) |
| **Methods** | | | |
| Protocol and registration | 5 | Indicate whether a review protocol exists; state if and where it can be accessed (e.g., a Web address); and if available, provide registration information, including the registration number. | Methods  (pg. 4) |
| Eligibility criteria | 6 | Specify characteristics of the sources of evidence used as eligibility criteria (e.g., years considered, language, and publication status), and provide a rationale. | Eligibility criteria  (pg. 4)  eTable 2 |
| Information sources^1^ | 7 | Describe all information sources of evidence used as eligibility criteria (e.g., databases with dates of coverage and contact with authors to identify additional sources), as well as the date the most recent search was executed. | Data sources and search strategy  (pg.5)  eTable3, eTable4 |
| Search | 8 | Present the full electronic search strategy for at least 1 database, including any limits used, such that it could be repeated. | Supplementary material  eTable 3 |
| Selection of sources of evidence^2^ | 9 | State the process for selecting sources of evidence (i.e., screening and eligibility) included in the scoping review. | Selection Process  (pg. 5) |
| Data chart process^3^ | 10 | Describe the methods of charting data from the included sources of evidence (e.g., calibrated forms or forms that have been tested by the team before their use, and whether data charting was done independently or in duplicate) and any processes for obtaining and confirming data from investigators. | Data extraction  (pg. 5) |
| Data items | 11 | List and define all variables for which data were sought and any assumptions and simplifications made. | Supplementary material  eTable5 |
| Critical appraisal of individual sources of evidence^4^ | 12 | If done, provide a rationale for conducting a critical appraisal of included sources of evidence; describe the methods used and how this information was used in any data synthesis (if appropriate). | NA |
| Synthesis of results | 13 | Describe the methods of handling and summarizing the data that were charted. | Data synthesis and presentation of results  (pg. 5) |
| **Results** | | | |
| Selection of sources of evidence | 14 | Give numbers of sources of evidence screened, assessed for eligibility, and included in the review, with reasons for exclusions at each stage, ideally using a flow diagram. | PRISMA diagram (pg. 21)  Supplementary material  eMethods 1 |
| Characteristics of sources of evidence | 15 | For each source of evidence, present characteristics for which data were charted and provide the citations. | Results  (pg. 5-7) |
| Critical appraisal within sources of evidence | 16 | Is done, present data on critical appraisal of included sources of evidence. | NA |
| Results of individual sources of evidence | 17 | For each included source of evidence, present the relevant data that were charted that related to the review questions and objectives. | Results  (pg. 5-7)  Table 1 |
| Synthesis of results | 18 | Summarize and/or present the charting results as they relate to the review questions and objectives. | Results  (pg. 5-7) |
| **Discussion** | | | |
| Summary of evidence | 19 | Summarize the main results (including an overview of concepts, themes, and types of evidence available), link to the review questions and objectives, and consider the relevance to key groups. | Discussion  (pg. 7-9) |
| Limitations | 20 | Discuss the limitations of the scoping review process. | Limitations  (pg. 9) |
| Conclusions | 21 | Provide a general interpretation of the results with respect to the review questions and objectives, as well as potential implications and/or next steps. | Conclusions  (pg. 10) |
| **Funding** | | | |
| Funding | 22 | Describe sources of funding for the included sources of evidence as well as sources of funding for the scoping review. Describe the role of the funders of the scoping review. | Title page |

^1^ Where sources of evidence (see second footnote) are compiled from, such as bibliographic databases, social media platforms, and Web sites.

^2^ A more inclusive/heterogeneous term used to account for the different types of evidence or data sources (e.g., quantitative and/or qualitative research, expert opinion, and policy documents) that may be eligible in a scoping review as opposed to only studies. This is not to be confused with information sources (see first footnote).

^3^ The frameworks by Arksey and O’Malley and Levac and colleagues and the JBI guidance refer to the process of data extraction as data charting.

^4^ The process of systematically examining research evidence to assess its validity, results, and relevance before using it inform a decision. This term is used for items 12 and 19 instead of “risk of bias” (which is more applicable to systematic review of interventions) to include and acknowledge the various sources of evidence that may be used in a scoping review (e.g., quantitative and/or qualitative research, expert opinion, and policy document.

**Citation:** Tricco AC, Lillie E, O'Brien KK, Colquhoun H, Levac D, et al. PRISMA Extension for Scoping Reviews (PRISMAScR): Checklist and Explanation. Ann Intern Med. 2018;169:467–73.

**Supplementary Table S2: Full inclusion and exclusion criteria**

|  | **In Scope 🡪 Include** | **Out of Scope 🡪 Exclude** |
| --- | --- | --- |
| **Population** | - Adults aged 18 and older - Not experiencing cancer-related symptoms (asymptomatic) | - Individuals <18 years of age - Experiencing cancer-related symptoms (i.e., symptomatic) |
| **Concept** | - Liquid biopsy and/or MCED tests that analyze biological fluids for the purposes of cancer screening - Potential outcomes could include:   - Clinical performance^1^ (sensitivity, specificity, PPV, NPV)   - Clinical utility^1^ (cancer-specific mortality, cancer detection, cancer stage)   - Health economic outcomes (cost-effectiveness, cost-benefit, cost-utility) | - Liquid biopsy and/or MCED tests for the purpose of triaging abnormal screening results, diagnosing cancer, selecting cancer therapy, monitoring cancer treatment response, or assessing cancer recurrence - Studies exclusively reporting on analytical validity of the test |
| **Context** | Conducted in countries rates as high or very high on HDI^2^ | Studies conducted in countries not rated high or very high on the HDI |
| **Study designs** | - Systematic reviews with or without meta-analyses^3^ - Rapid reviews^3^ - Scoping reviews^3^ - Experimental studies (e.g., RCTs) - Observational studies (e.g., cohort, case-control, cross-sectional) - Mixed methods - Modelling - Health economic analyses - Qualitative - Case reports - Case series | - Other evidence synthesis designs not listed on the left |
| **Document characteristics** | - Peer-reviewed studies - Grey literature - Reports - Conference proceedings and abstracts - Protocols - Standards, guidelines, policies, position statements - Published on or after 2014 - Published or available in English | - Animal studies - Commentaries, editorials, or letters to the editor - Dissertations - Literature reviews (i.e., unspecified narrative reviews) - Published before 2014 - Non-English publications |

HDI = Human Development Index; MCED = multi-cancer early detection; NPV = negative predictive value; PPV = positive predictive value; RCT = randomized controlled trial

^1^ For MCED tests, clinical utility outcomes could be reported by cancer site or for all cancer sites combined.

^2^ Human Development Index developed by the United Nations Development Programme.

^3^ In the event of overlap between primary research studies identified in the literature search with those examined in included evidence synthesis products (e.g., systematic reviews), only unique primary research studies that were not covered by an included evidence synthesis product were eligible for inclusion in this rapid scoping review. In the event a primary research study was reported in multiple papers, all papers providing data to address the research questions were included in this rapid scoping review.

**Supplementary Table S3: MEDLINE search strategy**

| **#** | **Search** |
| --- | --- |
| 1 | Liquid biopsy/ |
| 2 | (MCD or MCED or (("multi cancer*" or multicancer*) adj3 (detect* or screen* or test* or assay*))).ti,ab,kf. |
| 3 | ((liquid* or fluid or serum or plasma or blood or urine or multi-omics or cerebrospinal fluid or CSF) adj3 (screen* or test* or assay* or biops*)).ti,ab,kf. |
| 4 | or/1-3 |
| 5 | exp Neoplasms/ or Biomarkers, Tumor/ |
| 6 | (cancer* or tumo?r* or carcinoma* or malignan* or precancer* or "pre cancer*" or preinvasive* or pre invasive* or neoplas* or premalignan* or "pre malignan*" or malignan*).ti,ab,kf. |
| 7 | or/5-6 |
| 8 | Mass Screening/ |
| 9 | (screen* or (earl* adj detect*)).ti,ab,kf. |
| 10 | 8 or 9 |
| 11 | Early Detection of Cancer/ |
| 12 | 7 and 10 |
| 13 | 11 or 12 |
| 14 | 4 and 13 |
| 15 | exp Animals/ not Humans/ |
| 16 | 14 not 15 |
| 17 | limit 16 to (english language and yr="2014 -Current") |

Date run: March 4, 2024

**Supplementary Table S4: List of grey literature sources**

| **Website^1^** | **Keywords** |
| --- | --- |
| American Cancer Society | **Step 1:**   - Liquid biopsy - Multi-cancer early detection - MCD - MCED   **Step 2:**   - Cancer - Neoplas*   **Step 3:**   - Early detection - Screen* |
| Association for International Cancer Research |  |
| Canadian Agency for Drugs and technologies in Health |  |
| Canadian Cancer Society |  |
| Cancer Council Australia |  |
| Cancer Research UK |  |
| ClinicalTrials.gov |  |
| European Cancer Organization |  |
| European Organisation for Research and Treatment of Cancer |  |
| International Cancer Screening Network |  |
| International Clinical Trials Registry Platform |  |
| International Health Technology Database |  |
| National Cancer Institute/National Institutes of Health |  |
| U.S. Food and Drug Administration |  |
| World Health Organization |  |

^1^ Grey literature sources were searched from February to March 2024.

**Supplementary Table S5: Data extraction items**

| **Category** | **Variables** |
| --- | --- |
| Publication details | - Last name of first author - Year of publication |
| Study characteristics | - Objectives/aims - Study dates - Design (e.g., systematic review, RCT, prospective cohort) - Geographic location - Study name (if applicable) - Sample size - Funding source - Clinical trial registration (if applicable) - Names of related/connected studies - Follow-up duration |
| Participant characteristics | - Inclusion and exclusion criteria - Age at study enrollment - Sex |
| Intervention characteristics | - Biological fluid sampled (e.g., blood, urine, stool) - Biomarker target (e.g., ctDNA, cfDNA) - Test name (e.g., Galleri, Cologuard) - Parameters detected (e.g., altered DNA, mutations, methylation patterns) - Type(s) of cancer detected - Method(s) used to confirm presence and/or absence of cancer - Follow-up protocol |
| Comparator characteristics | - Type of comparison (e.g., standard screening test) - Screening inclusion and exclusion criteria - Methods used to confirm presence and/or absence of cancer - Follow-up protocol |
| Outcome characteristics | - Reported outcome(s) - Outcome definition - Method of assessment - Analysis type (e.g., intent to treat) - Main results |

cfDNA = cell-free DNA; ctDNA = circulating tumour DNA; DNA = deoxyribonucleic acid; RCT = randomized controlled trial

**Supplementary Methods: Full text exclusions and reasoning**

**Population Out of Scope (n= 45)**

1. Abern MR, Owusu R., Inman BA. Clinical performance and utility of a DNA methylation urine test for bladder cancer. Urol Oncol. 2014;31(1):51.e21–6.
2. Alijaj N, Pavlovic B, Martel P, Rakausas A, Cesson V, Saba K, et al. Identification of urine biomarkers to improve eligibility for prostate biopsy and detect high-grade prostate cancer. Cancers. 2022;14(5):1135.
3. Bando H, Nakamura Y, Kotani D, Yoshino Y. Comparing GOZILA and COLOMATE: Ongoing umbrella/basket trials examining genetic testing in gastrointestinal malignancies. Oncology. 2021;35(7):382–89.
4. Bhakdi SC, Suriyaphol P, Thaicharoen P, Grote STK, Komoltri C, Chaiyaprasithi B, et al. Accuracy of tumour-associated circulating endothelial cells as a screening biomarker for clinically significant prostate cancer. Cancers. 2019;11(8):1064.
5. Borg M, Wen SWC, Andersen RF, Timm S, Hansen TF, Hilberg O. Methylated circulating tumor DNA in blood as a tool for diagnosing lung cancer: A systematic review and meta-analysis. Cancers. 2023;15(15):3959.
6. Buckley DN, Lewinger, JP, Gooden G, Spillman M, Neuman M, Guo XM, et al. OvaPrint – A cell-free DNA methylation liquid biopsy for the risk assessment of high-grade serous ovarian cancer. Clin Cancer Res. 2023;29(24):5196 –206.
7. Cameron JM, Sala A, Antoniou G, Brennan PM, Butler HJ, Conn JJA, et al. A spectroscopic liquid biopsy for the earlier detection of multiple cancer types. Br J Cancer. 2023;129(10):1658–66.
8. Cameron JM, Brennan PM, Antoniou G, Butler HJ, Christie L, Conn JJA, et al. Clinical validation of a spectroscopic liquid biopsy for early detection of brain cancer. Neurooncol Adv. 2022;4(1):vdac024.
9. Carter HE, Jeffrey GP, Ramm GA, Gordon LG. Cost-effectiveness of a serum biomarker test for risk-stratified liver ultrasound screening for hepatocellular carcinoma. Value Health. 2021;24(10):1454–462.
10. Chen CH, Yan SL, Yang TH, Chen SF, Yeh YH, Ou JJ, et al. The relationship between the methylated septin-9 DNA blood test and stool occult blood test for diagnosing colorectal cancer in Taiwanese people. J Clin Lab Anal. 2017;31(1):e22013.
11. Chen S, Wang Y, Wang K, Zhang L, Zhang X. Circulating tumor DNA-based early detection of precancers colorectal lesions using QClamp XNA-mediates real-time PCR. Pharmazie. 2021;76(12):606–10.
12. Chhatwal J, Samur S, Yang JD, Roberts LR, Nguyen MH, Ozbay AB, et al. Effectiveness of HCC surveillance programs using multitarget blood test: A modelling study. Hepatol Commun. 2023;7(6):e0146.
13. Cock C, Anwar S, Byrne SE, Meng R, Pedersen S, Fraser RJ, et al. Low sensitivity of fecal immunochemical tests and blood-based markers of DNA hypermethylation for detection of sessile serrated adenomas/polyps. Dig Dis Sci. 2019;64(9):2555–62.
14. Couraud S, Vaca-Paniagua F, Villar S, Oliver J, Schuster T, Blanché H, et al. noninvasive diagnosis of actionable mutations by deep sequencing of circulating free DNA in lung cancer from never-smokers: a proof-of-concept study from BioCAST/IFCT-1002. Clin Cancer Res. 2014;20(17);4613–24.
15. Dhani H, Hinestrosa JP, Inzaguirre-Carbonell J, Balcer HI, Kurzrock, Billings PR. Case report: Early detection of pancreatic pre-cancer lesion in multimodal approach with exosome liquid biopsy. Front Oncol. 2023;13:1170513.
16. Gelli E, Fallerini C, Valentino F, Giliberti A, Castiglione F, Laschi L, et al. RB1 germline variant predisposing to a rare ovarian germ cell tumor: A case report. Front Oncol. 2020;10:1467.
17. Hussain Y, Bannaga A, Fisher N, Krishnamoorthy A, Kimani P, Malik A, et al. The fatty lover, cirrhosis, and liver cancer study (TENDENCY): Protocol for a multicenter case-control study. JMIR Res Protoc. 2013;12:e44264.
18. Jovelet C, Ileana E, Le Deley MC, Motté N, Rosellini S, Romero A, et al. Circulating cell-free tumor DNA analysis of 50 genes by next-generation sequencing in the prospective MOSCATO trial. Clin Cancer Res. 2016;22(12):2960–80.
19. Kojic M, Maybury MK, Waddell N, Koufariotis LT, Addala V, Millar A, et al. Efficient detection and monitoring of pediatric brain malignancies with liquid biopsy based on patient-specific somatic mutation screening. Neuro Oncol. 2023;25(8):1507–17.
20. Lathlean TJH, Wassie MM, Winter JM, Goyal R, Young GP, Symonds EL. Accuracy of blood-based biomarkers for screening precancerous colorectal lesions: A protocol for systematic review and meta-analysos. BMJ Open. 2022;12(5):e060712.
21. Lee SY, Chae DK, Lee SH, Lim Y, An J, Chae CH, et al. Efficient mutation screening for cervical cancers from circulating tumor DNA in blood. BMC Cancer. 2020;20(1):694.
22. Liu C, Chen H, Sun T, Wang H, Chen B, Wang X. The value of circulating tumor cells with positive centromere probe 8 in the diagnosis of small pulmonary nodules. Transl Oncol. 2021;14(5):101052.
23. Lorenzi AT, Fregnani HJT, Dockter J, Fitzgerald K, Strohecker E, Easton B, et al. High-risk human papillomavirus detection in urine samples from a referral population with cervical biopsy-proven high-grade lesions. J Low Genit Tract Dis. 2018;22(1):17–20.
24. Malczewska A, Bodei L, Kidd M, Modlin IM. Blood mRNA measurement (NETest) for neuroendocrine tumor diagnosis of image-negative liver metastatic disease. J Clin Endocrinol Metab. 2019;104(3):867–72.
25. Marquette C-H, Boutros J, Benzaquen J, Ferreira M, Pastre J, Pison C, et al. Circulating tumour cells as a potential biomarker for lung cancer screening: A prospective cohort study. Lancet Respir Med. 2020;8(7):790–16.
26. Minnee P, Hessels D, Schalken JA, Van Criekinge W. Clinically significant prostate cancer diagnosed using a urinary molecular biomarker-based risk score: Two case reports. BMC Urol. 2019;19(1):124.
27. Möller A, Olsson H, Grönberg H, Eklund M, Aly M, Nordström T. The Stockholm3 blood-rest predicts clinically-significant cancer on biopsy: Independent validation in a multi-center community cohort. Prostate Cancer Prostatic Dis. 2019;22(1):137–42.
28. Nicholls JM, Lee VHF, Chan SK, Tsang KC, Choi CW, Kwong DLW, et al. Negative plasma Epstein-Barr virus DNA nasopharyngeal carcinoma in an endemic region and its influence on liquid biopsy screening programmes. Br J Cancer. 2019;121(8):690–98.
29. Nicolazzo C, Colangelo L, Corsi A, Carpino G, Gradilone A, Sonato C, et al. Liquid biopsy in rare cancers: Lessons from hemangiopericytoma. Anal Cell Pathol (Amst). 2018;2018:9718585.
30. O’Reilly E, Tuzova AV, Walsh AL, Russell NM, O’Brien O, Kelly S, et al. epiCapture: A urine DNA methylation test for early detection of aggressive prostate cancer. JCO Precis Oncol. 2019;2019:PO.18.00134.
31. Ørntoft M-BW, Nielsen HJ, Ørntoft TF, Andersen CL, Danish Study Group on Early Detection of Colorectal Cancer. Performance of the colorectal cancer screening marker Sept9 is influenced by age, diabetes and arthritis: A nested case-control study. BMC Cancer. 2015;15:819.
32. Peabody J, Rahim A, Wilcox B, McGehee C, Estigarribia E, Paculdo D, et al. Clinical utility of a blood-based protein assay on diagnostic colonoscopy referrals for elevated-risk colorectal cancer patients in primary care. Am J Clin Oncol. 2019;42(9):687–91.
33. Peabody J, Paculdo D, Swagel E, Fugaro S, Tran M. Clinical utility of a blood-based protein assay to increase screening of elevated-risk patients for colorectal cancer in the primary care setting. J Cancer Res Clin Oncol, 2017;143(11):2301–07.
34. Rodriguez Pena MDS, Springer SU, Taheri D, Li L, Tregnago AC, Eich Ml, et al. Performance of novel non-invasive urine assay UroSEEK in cohorts of equivocal urine cytology. Virchows Arch. 2020;476(3):423–29.
35. Senkomago V, Des Marais AC, Rahangdale L, Vibat CRT, Erlander MG, Smith JS. Comparison of urine specimen collection times and testing fractions for the detection of high-risk human papillomavirus and high-grade cervical precancer. J Clin Virol. 2016;74:26–31.
36. Singal AG, Haaland B, Parikh ND, Ozbay AB, Kirshner C, Chakankar S, et al. Comparison of a multitarget blood test to ultrasound and alpha-fetoprotein for hepatocellular carcinoma surveillance: Results of a network meta-analysis. Hepatol Commun. 2022;6(10):2925–36.
37. Snoek BC, van Splunter AP, Bleeker MCG, van Ruiten MC, Heideman DAM, Rurup WF, et al. Cervical cancer detection by DNA methylation analysis in urine. Sci Rep. 2019;9(1):3088.
38. Song X, Gong J, Zhang X, Feng X, Huang H, Gao M, et al. Plasma-based early screening and monitoring of EGFR mutations in NSCLC patients by a 3-color digital PCR assay. Br J Cancer. 2020;123(9):1437–44.
39. Steuber T, Tennstedt P, Macagno A, Athanasiou A, Wittig A, Huber R, et al. Thrombospondin 1 and cathepsin D improve prostate cancer diagnosis by avoiding potentially unnecessary prostate biopsies. BJU Int. 2019;123(5):826–33.
40. Sun J, Xu J, Sun C, Zheng M, Li Y, Zhu S, et al. Screening and prognostic value of methylated Septin9 and its association with clinicopathological and molecular characteristics in colorectal cancer. Front Mol Biosci. 2021;8:568818.
41. Sun J, Fei F, Zhang M, Li Y, Zhang X, Zhu S, et al. The role of mSEPT9 in screening, diagnosis, and recurrence monitoring of colorectal cancer. BMC Cancer. 2019;19(1):450.
42. Symonds EL, Pedersen SK, Baker RT, Murray DH, Gaur S, Cole SR, et al. A blood test for methylated BCAT1 and IKZF1 vs. a fecal immunochemical test for detection of colorectal neoplasia. Clin Transl Gastroenterol. 2016;7(1):e137.
43. Zhan Y, Wang S, Yuan Z, Zhao X, Ni K, Xin R, et al. The stool *syndecan2* methylation test is more robust than blood tests for methylated *septin9,* CEA, CA19-9 and CA724: A diagnostic test for the early detection of colorectal neoplasms. Transl Cancer Res. 2023;12(1):65–77.
44. Lee MJ, Hueniken K, Kuehne N, Li L, Jiang SX, Said BI, et al. Cancer patient-reported preferences and knowledge for liquid biopsies and blood biomarkers at a comprehensive cancer center. Cancer Manag Res. 2020;12:1163–73.
45. McKiernan J, Donovan MJ, Margolis E, Partin A, Carter B, Brown G, et al. Prospective adaptive utility trial to validate performance of a novel urine exosome gene expression assay to predict high-grade prostate cancer in patients with prostate-specific antigen 2-10ng/ml at initial biopsy. Eur Urol. 2018;74(6):731–38.

**Concept Out of Scope (n = 321)**

46. Abd El Kader L, Soliman AH, Salem SE, Akel SY, Ibrahim NH. The clinical significance of septin 9 and colon cancer specific antigen-2 (CCSA-2) in colorectal cancer. Asian Pac j Cancer Prev. 2023;24(3):1027–36.
47. Abdollah F, Dalela D, Haffner MC, Culig Z, Schalken J. The role of biomarkers and genetics in the diagnosis of prostate cancer. Eur Urol Focus. 2015;1(2):99–108.
48. Ačkar L, Casjens S, Andreas A, Raiko I, Brüning T, Geffken M, et al. Blood-based detection of lung cancer using cysteine-rich angiogenic inducer 61 (CYR61) as a circulating protein biomarker: A pilot study. Mol Oncol. 2021;15(11):2877–90.
49. Adamo P, Cowley CM, Neal CP, Mistry V, Page K, Dennison AR, et al. Profiling tumour heterogeneity through circulating tumour DNA in patients with pancreatic cancer. Oncotarget. 2017;8(50):87221–33.
50. Agarwal D, Covarrubias-Zambrano O, Bossmann SH, Natarajan B. Early detection of pancreatic cancers using liquid biopsies and hierarchical decision structure. IEEE J Transl Eng Health Med. 2022;10:4300208.
51. Ali H, Harting R, de Vries R, Ali M, Wurdinger T, Best MG. Blood-based biomarkers for glioma in the context of gliomagenesis: A systematic review. Front Oncol. 2021;11:665235.
52. Alizadeh-Sedigh M, Fazeli MS, Mahmoodzadeh H, Sharif SB, Teimoori-Toolabi L. Methylation of FBN_1_, SPG_20_, ITF_2_, RUNX_3_, SNCA, MLH_1_, AND SEPT_9_ genes in circulating cell-free DNA as biomarkers of colorectal cancer. Cancer Biomark. 2022;34(2):221–50.
53. Allenson K, Castillo J, San Lucas FA, Scelo G, Kim DU, Bernard V, et al. High prevalence of mutant KRAS in circulating exosome-derived DNA from early-stage pancreatic cancer patients. Ann Oncol. 2017;28(4):741–47.
54. Alunni-Fabbroni M, Weber S, Öcal O, Seidensticker M, Mayerle J, Malfertheiner P, et al. Circulating cell-free DNA combined to magnetic resonance imaging for early detection of HCC in patients with liver cirrhosis. Cancers. 2021;13(3):521.
55. Andersson J, Palsdottir T, Lantz Z, Aly M, Grönberg H, Egevad L, et al. Digital rectal examination in Stockholm3 biomarker-based prostate cancer screening. Eur Urol Open Sci. 2022;44:69–75.
56. Arasaradnam RP, McFarlane MJ, Ryan-Fisher C, Westenbrink E, Hodges P, Thomas MG, et al. Detection of colorectal cancer (CRC) by urinary volatile organic compound analysis. PLoS One. 2014;9(9):e108750.
57. Araújo AL, Santos-Silva, Kowalski LP. Diagnostic accuracy of liquid biopsy for oral potentially malignant disorders and head and neck cancer: An overview of systematic reviews. Curr Oncol Rep. 2023;25(4):279–92.
58. Asante DB, Calapre L, Ziman M, Meniawy TM, Gray ES. Liquid biopsy in ovarian cancer using circulating tumor DNA and cells: Ready for prime time? Cancer Lett. 2020;468:59–71.
59. Bae M, Kim G, Lee TR, Ahn JM, Park H, Park SR, et al. Integrative modeling of tumor genomes and epigenomes for enhanced cancer diagnosis by cell-free DNA. Nat Commun. 2023;14(1):2017.
60. Bai S, Lin S, Lin T, Wang Q, Cheng C, Lin J, et al. Clinical diagnostic biomarker "circulating tumor cells" in breast cancer - A meta-analysis. Front Oncol. 2023;13:1137519.
61. Bai Y, Xu J, Li D, Zhang X, Chen D, Xie F, et al. HepaClear, a blood-based panel combining novel methylated CpG sites and protein markers, for the detection of early-stage hepatocellular carcinoma. Clin Epigenetics. 2023;15(1):99.
62. Baksh M, Mahajan B, Dufresne MM, Shoukry MM, Nussbaum S, Abbaszadeh-Kasbi A, et al. Circulating tumor DNA for breast cancer: Review of active clinical trials. Cancer Treat Res Commun. 2022;32:100609.
63. Bartkowiak K, Heidrich I, Kwiatkowski M, Banys-Paulchowski M, Andreas A, Wurlitzer M, et al. Circulating cellular communication network factor 1 protein as a sensitive liquid biopsy marker for early detection of breast cancer. Clin Chem. 2022;68(2):344–53.
64. Beaulieu J-F, Herring E, Kanaoka S, Tremblay É. Use of integrin alpha 6 transcripts in a still mRNA assay for the detection of colorectal cancers at curable stages. Oncotarget. 2016;7(12):14684–92.
65. Bhardwaj M, Weigal K, Tikk K, Benner A, Schrotz-King P, Brenner H. Multiplex screening of 275 plasma protein biomarkers to identify a signature for early detection of colorectal cancer. Mol Oncol. 2020;14(1):8–21.
66. Bhardwaj M, Gies A, Werner S, Schrotz-King, Brenner H. Blood-based protein signatures for early detection of colorectal cancer: A systematic review. Clin Transl Gastroenterol. 2017;8(11):e128.
67. Bhongir AV, Sampath S, Bonthapally RK, Gudivada KK, Ramaswamy G. Sequential application and post-test probability for screening of bladder cancer using urinary proteomic biomarkers: A review based probabilistic analysis. Asian Pac J Cancer Prev. 2023;24(6): 2021–27.
68. Birse CE, Tomic JL, Pass HI, Rom WN, Lagier RJ. Clinical validation of a blood-based classifier for diagnostic evaluation of asymptomatic individuals with pulmonary nodules. Clin Proteomics. 2017;14:25.
69. Blyuss O, Burnell M, Ryan A, Gentry-Maharaj A, Mariño IP, Kalsi J, et al. Comparison of longitudinal CA125 algorithms as a first-line screen for ovarian cancer in the general population. Clin Cancer Res. 2018;24(19):4726–33.
70. Boylan KLM, Geschwind K, Koopmeiners JS, Geller MA, Starr TK, Skubitz APN. A multiplex platform for the identification of ovarian cancer biomarkers. Clin Proteomics. 2017;14:34.
71. Brait M, Banerjee M, Maldonado L, Ooki A, Loyo M, Guida E, et al. Promotor methylation of MCAM, Erα and Erβ in serum of early stage prostate cancer patients. Oncotarget. 2017;8(9):15431–440.
72. Brenne SS, Madsen PH, Pedersen IS, Hveem K, Skorpen F, Krarup HB, et al. Colorectal cancer detected by liquid biopsy 2 years prior to clinical diagnosis in the HUNT study. Br J Cancer. 2023;129(5):861–68.
73. Brentnall AR, Mathews C, Beare S, Ching J, Sleeth M, Sasieni P. Dynamic data-enabled stratified sampling for trial invitations with application in NHS-Galleri. Clin Trials. 2023;20(4):425–33.
74. Brikun I, Nusskern D, Decatus A, Harvey E, Li L, Freije D. A panel of DNA methylation markers for the detection of prostate cancer from FV and DRE urine DNA. Clin Epigenetics. 2018;10:91.
75. Brikun I, Nusskern D, Freije D. An expanded biomarker panel for the detection of prostate cancer from urine DNA. Exp Hematol Oncol. 2019;8:13.
76. Burg S, Smeets R, Gosau M, Failing K, Grust ALC. Case report: Early detection of lung carcinoid in an asymptomatic individual by blood-test initiated PET-CT imaging. Front Oncol. 2023;13:1177237.
77. Cao B, Liu L, Xhang R, Dong H, Shen J. Sensitivity and specificity of folate receptor α-positive circulating tumour cells in gastric cancer. Postgrad Med J. 2024;100(1180):112–19.
78. Causin RL, sa Silva LS, Evangelista AF, Leal LF, Souza KCB, Pessôa-Pereira D, et al. MicroRNA biomarkers of high-grade cervical intraepithelial neoplasia in liquid biopsy. Biomed Res Int. 2021;2021:6650966.
79. Chakravarti P, Maheshwari A, Tahlan S, Kadam P, Bagal S, Gore S, et al. Diagnostic accuracy of menstrual blood for human papillomavirus detection in cervical cancer screening: A systematic review. Ecancermedicalscience. 2022;16:1427.
80. Chalfin HJ, Glavaris SA, Gorin MA, Kates MR, Fong MH, Dong L, et al. Circulating tumor cell and circulating tumor DNA assays reveal complementary information for patients with metastatic urothelial cancer. Eur Urol Oncol. 2021;4(2):310–14.
81. Chan KCA, Woo JKS, King A, Zee BCY, Lam WKJ, Chan SL, et al. Analysis of plasma Epstein-Barr virus DNA to screen for nasopharyngeal cancer. N Engl J Med. 2017;377(6):513–22.
82. Chang P-Y, Chen C-C, Chang Y-S, Tsai W-S, You J-F, Lin G-P, et al. MicroRNA-223 and microRNA-92a in stool and plasma samples act as complementary biomarkers to increase colorectal cancer detection. Oncotarget. 2016;7(9):10663–75.
83. Chang Y, Li S, Li Z, Wang X, Chang F, Geng S, et al. Non-invasive detection of lymphoma with circulating tumor DNA features and protein tumor markers. Front Oncol. 2024;14:1341997.
84. Chen H, Werner S, Butt J, Zöring I, Knebel P, Michel A, et al. Prospective evaluation of 64 serum autoantibodies as biomarkers for early detection of colorectal cancer in a true screening setting. Oncotarget. 2016;7(13):16420–32.
85. Chen L, Pan XY, Zeng T, Zhang Y-H, Zhang YH, Huang T, et al. Immunosignature screening for multiple cancer subtypes based on expression rule. Front Bioeng Biotechnol. 2019;7:370.
86. Chen W-J, Xu W-N, Wang H-Y, Chen X-X, Li X-Q, Xie S-H, et al. Plasma Epstein-Barr virus DNA and risk of nasopharyngeal carcinoma in a prospective seropositive population. BMC Cancer. 2021;21(1):651.
87. Chen X-X, Chen W, Liu Y-L., Lin C-X, Li M, Chen W-J, et al. Development and validation of a flexible DNA extraction (PAN) method for liquid biopsy of multiple sample types. J Clin Lab Anal. 2021;35(9):e23962.
88. Chen Y, Xie Y, Bi L, Ci H, Li W, Liu D. A novel serum m7G-harboring microRNA signature for cancer detection. Front Genet. 2024;15:1270302.
89. Chen A-J, You G-R, lee C-J, Lu Y-C, Tang, S-J, Huang Y-F. Systemic investigation identifying salivary miR-196b as a promising biomarker for early detection of head-neck cancer and oral precancer lesions. Diagnostics. 2021;11(8):1411.
90. Cheng X, Liu X, Liu X, Guo Z, Sun H, Zhang M, et al. Metabolomics of non-muscle invasive bladder cancer: Biomarkers for early detection of bladder cancer. Front Oncol. 2018;8:494.
91. Cheng YC, Wu PH, Chen YJ, Yang CH, Huang JL, Chou YC, et al. Using comorbidity patten analysis to detect reliable methylated genes in colorectal cancer verified by stool DNA test. Genes. 2021;12910):1539.
92. Cheng Z, Wang Y, Wei W, Liu Y, Zhou Z. Correlation between stool DNA-based SDC2 methylation tests combined with tumor markers with pathological features in colorectal cancer. Clin Lab. 2023;69(7).
93. Cheung AH-K, Chow C, To K-F. Latest development of liquid biopsy. J Thorac Dis. 2018;10(Suppl 14):S1645–S1651.
94. Cho H-W, Shim SR, Lee JK, Hong JH. Accuracy of human papillomavirus tests on self-collected urine versus clinician-collected samples for the detection of cervical precancer: A systematic review and meta-analysis. J Gynecol Oncol. 2022;33(1):e4.
95. Choi YS, Jin H, Lee KE. Usefulness analysis of urine samples for early screening of human papilloma virus infection. J Cancer Prev. 2019;24(4):240–44.
96. Cirillo PDR, Margiotti K, Fabiani M, Barros-Filho MC, Sparacino D, Cima A, et al. Multi-analytical test based on serum miRNAs and proteins quantification for ovarian cancer early detection. PLoS One. 2021;16(8):e0255804.
97. Cohen JD, Javed AA, Thoburn C, Wong F, Tie J, Gibs P, et al. Combined circulating tumor DNA and protein biomarker-based liquid biopsy for the earlier detection of pancreatic cancers. Proc Natl Acad Sci USA. 2017;114(38):10202–07.
98. Cree IA, Uttley L, Woods HB, Kituchi H, Reiman A, Harnan S, et al. The evidence base for circulating tumour DNA blood-based biomarkers for the early detection of cancer: A systematic mapping review. BMC Cancer. 2017;17(1):697.
99. Cui X, Liu T, Xu X, Zhao Z, Tian Y, Zhao Y, et al. Label-free detection of multiple genitourinary cancers from urine by surface-enhanced Raman spectroscopy. Spectrochim Acta A Mol Biomol Spectrosc. 2020;240:118543.
100. Dabbous HK, Mohamed YAE-R, El-Folly RF, El-Talkawy MD, Seddik HE, Johar D, et al. Evaluation of fecal m2PK as a diagnostic marker in colorectal cancer. J Gastrointest Cancer. 2019;50(3):442–50.
101. Damerla RR, Lee NY, You D, Soni R, Shah R, Reyngold M, et al. Detection of early human papillomavirus-associated cancers by liquid biopsy. JCO Precis Oncol. 2019;3:PO.18.00276.
102. Das C, Mukhopadhyay M, Ghosh T, Saha AK, Sengupta M. Correlation of cytohistlogical expression and serum level of ca125 in ovarian neoplasm. J Clin Diagn Res. 2014;8(3):41–3.
103. Das D, Hirayama S, Aye L, Bryan ME, Naegele S, Zhao B, et al. Blood-based screening for HPV-associated cancers. 2024:2024.01.04.24300841.
104. das Nair R, Orr KS, Vedhara K, Kendrick D. Exploring recruitment barriers and facilitators in early cancer detection trials: The use of pre-trial focus groups. Trials. 2014;15:98.
105. De Chiara L, Barcia-Castro L, Gallardo-Gó mez M, de la Cadena MP, Martínez-Zorzano VS, Rodríguez-Berrocal F, et al. Evaluation of blood soluble CD26 as a complementary biomarker for colorectal cancer screening programs. Cancers. 2022;14(19):4563.
106. De Chiara L, de la Cadena M, Rodríguez-Berrocal J, Alvarez-Pardiñas MC, Pardiñas-Añón MC, Varela-Calviño R, et al. CD26-related serum biomarkers: sCD26 protein, DPP4 activity, and anti-CD26 isotope levels in a colorectal cancer-screening context. Dis Markers. 2020;2020:4347936.
107. Debernardi S, O’Brien H, Algahmdi AS, Malats N, Stweart GD, Plješa-Ercegovac M, et al. A combination of urinary biomarker panel and PancRISK score for earlier detection of pancreatic cancer: A case-control study. PLoS Med. 2020;17(12):e1003489.
108. Deng L, Chao H, Deng H, Yu Z, Zhao R, Huang L, et al. A novel and sensitive DNA methylation marker for the urine-based liquid biopsies to detect bladder cancer. BMC Cancer. 2022;22(1):510.
109. Dollinger MM, Behl S, Fleig WE. Early detection of colorectal cancer: A multi-center pre-clinical case cohort study for validation of a combined DNA stool test. Clin Lab. 2018;64(10):1719–30.
110. Du S, Zhao Y, Lv C, Wei M, Gao Z, Meng X. Applying serum proteins and microRNA as novel biomarkers for early-stage cervical cancer detection. Sci Rep. 2020;10(1):9033.
111. Duque G, Manterola C, Otzen T, Arias C, Palacios D, Mora M, et al. Cancer biomarkers in liquid biopsy for early detection of breast cancer: A systematic review. Clin Med Insights Oncol. 2022;16:11795549221134831.
112. Eikenboom EL, Wilting SM, Deger T, Srebniak MI, Van Veghel-Plandsoen M, Boers RG, et al. Liquid biopsies for colorectal cancer and advanced adenoma screening and surveillance: What to measure? Cancers. 2023;15(18):4607.
113. Endzelinš E, Berger A, Melne V, Bajo-Santos C, Soboļevska K, Ābols A, et al. Detection of circulating miRNAs: Comparative analysis of extracellular vesicle-incorporated miRNAs and cell-free miRNAs in whole plasma of prostate cancer patients. BMC Cancer. 2017;17(1):730.
114. Endzelinš E, Melne V, Kalnina Z, Lietuvietis V, Riekstina U, Llorente A, et al. Diagnostic, prognostic and predictive value of cell-free miRNAs in prostate cancer: A systematic review. Mol Cancer. 2016;15(1):41.
115. Enikeev D, Morozov A, Babaevskaya D, Bazarkin A, Malavaud B. A systematic review of circulating tumor cells clinical application in prostate cancer diagnosis. Cancers. 2022;14(15):3802.
116. Enroth S, Ivansson E, Lindberg JH, Lycke M, Bergman J, Reneland A, et al. Data-driven analysis of a validated risk score for ovarian cancer identifies clinically distinct patterns during follow-up and treatment. Commun Med. 2022;1:124.
117. Erben V, Poschet G, Schrotz-King P, Brenner H. Comparing metabolomics profiles in various types of liquid biopsies among screening participants with and without advanced colorectal neoplasms. Diagnostics. 2021;11(3):561.
118. Faversani A, Favero C, Dioni L, Pesatori AC, Bollati V, Montoli M, et al. An EBC/plasma miRNA signature discriminates lung adenocarcinomas from pleural mesothelioma and healthy controls. Front Oncol. 2021;11:643280.
119. Fietz S, Diekmann E, de Vos L, Zarbl R, Hunecke A, Glosch A-K, et al. Circulating cell-free SHOXz DNA methylation is a predictive, prognostic, and monitoring biomarker in adjuvant and palliative anti-PD-1-treated melanoma. Clin Chem. 2023;70(3):516–27.
120. Fontenot HB. Urine-based HPV testing as a method to screen for cervical cancer. Nurs Womens Health. 2015;19(1):59–65.
121. Fortunato O, Huber V, Segale M, Cova A, Vallacchi V, Squarcina P, et al. Development of a molecular blood-based immune signature classifier as biomarker for risks assessment in lung cancer screening. Cancer Epidemiol Biomarkers Prev. 2022;31(11):2020–29.
122. Freitas DLD, Câmara IM, Silva PP, Wanderley NRS, Alves MBC, Morais CLM, et al. Spectrochemical analysis of liquid biopsy harnessed to multivariate analysis towards breast cancer screening. Sci Rep. 2020;10(1):12818.
123. Funderburk K, Bang-Christensen SR, Miller BF, Tan H, Margolin G, Petrykowska HM, et al. Evaluating stacked methylation markers for blood-based multicancer detection. Cancers. 2023;15(19):4826.
124. Fung KYC, Tabor B, Buckley MJ, Priebe IK, Purins L, Pompeia C, et al. Blood-based protein biomarker panel for the detection of colorectal cancer. PLoS One. 2015;10(3):e0120425.
125. Gallardo-Gómez M, Rodríguez-Gironso, Planell N, Moran S, Bujanda L, Etxart A, et al. Serum methylation of GALNT9, UPF3A, WARS, and LDB2 as noninvasive biomarkers for the early detection of colorectal cancer and advanced adenomas. Clin Epigenetics. 2023;15(1):157.
126. Gallardo-Gómez M, Moran S, de la Cadena MP, Martínez-Zorzano VS, Rodríguez-Berrocal FJ, Rodríguez-Girondo M, et al. A new approach to epigenome-wide discovery of non-invasive methylation biomarkers for colorectal cancer screening in circulating cell-free DNA using pooled samples. Clin Epigenetics. 2018;10:53.
127. Gao H, Shen Y, Feng Z, Cai Y, Yang J, Zhu Y, et al. The clinical implications of circulating microRNAs as potential biomarkers in screening oral squamous cell carcinoma. Front Oncol. 2022;12:965357.
128. Gao K, Eurasian M, Zhang J, Wei Y, Zheng Q, Ye H, et al. Can genomic amplification of human telomerase gene and C-MYC in liquid-based cytological specimens be used as a method for opportunistic cervical cancer screening. Gynecol Obstet Invest. 2015;80(3):153–63.
129. Gao Y, Zhao H, An K, Liu Z, Hai L, Li R, et al. Whole-genome bisulfate sequencing analysis of circulating tumour DNA for the detection and molecular classification of cancer. Clin Transl Med. 2022;12(8):e1014.
130. Gentry-Maharaj A, Blyuss O, Ryan A, Burnell M, Karpinskyj C, Gunu R, et al. Multi-marker longitudinal algorithms incorporating HE4 and CA125 in ovarian cancer screening of postmenopausal women. Cancers. 2020;12(7):1931.
131. Gonçalves E, Gonçalves-Reis M, Pereira-Leal JB, Cardoso J. DNA methylation fingerprint of hepatocellular carcinoma from tissue and liquid biopsies. Sci Rep. 2022;12(1):11512.
132. Gonzalez T, Nie Q, Chaudhary LN, Basel D, Reddi HV. Methylation signatures as biomarkers for non-invasive early detection of breast cancer: A systematic review of the literature. Cancer Genet. 2024;282–283:1–8.
133. Gorski JW, Dietrich CS, Davis C, Erol L, Dietrich H, Per NJ, et al. Significance of pelvic fluid observed during ovarian cancer screening with transvaginal ultrasound. Disgnostics. 2022;12(1):144.
134. Grobbee EJ, Lan SY, Fuhler GM, Blakaj B, Konstantinov SR, Bruno MJ, et al. First steps towards combining faecal immunochemical testing with the gut microbiome in colorectal cancer screening. United European Gastroenterol J. 2020;8(3):293–302.
135. Guan X, Du Y, Ma R, Teng N, Ou S, Zhao H, et al. Construction of the XGBoost model for early lung cancer prediction based on metabolic indices. BMC Med Inform Decis Mak. 2023;23(1):107.
136. Guo XM, Miller H, Matsuo K, Roman LD, Salhia B. Circulating cell-free DNA methylation profiles in the early detection of ovarian cancer: A scoping review of the literature. Cancers. 2021;13(4):838.
137. Haan D, Bergamaschi A, Friedl V, Guler GD, Ning Y, Reggiardo R, et al. Epigenomic blood-based early detection of pancreatic cancer employing cell-free DNA. Clin Gasterotenterol Hepatol. 2023;21(7):1802–09.
138. Hallermayr A, Wohlfrom T, Steinke-Lange V, Benet-Pagès A, Schaft F, Heitzer E, et al. Somatic copy number alteration and fragmentation analysis in circulating tumor DNA for cancer screening and treatment monitoring in colorectal cancer patients. J Hematol Oncol. 2022;15(1):125.
139. Hancox J, Ayling K, Bedford L, Vedhara K, Robertson JFR, Young B, et al. Psychological impact of lung cancer screening using a novel antibody blood test followed by imaging: the ECLS randomized controlled trial. J Public Health. 2023;45(2):e275–e284.
140. Harlid S, Gunter MJ, Van Guelpen B. Risk-predictive and diagnostic biomarkers for colorectal cancer; a systematic review of studies using pre-diagnostic blood samples collected in prospective cohorts and screening settings. Cancers. 2021;13(17):4406.
141. Hermoso-Durán S, Domper-Arnal MJ, Roncales P, Vega S, Sanchez-Garcia O, Ojeda JL, et al. Bowel preparation for colonoscopy changes serum composition as detected by thermal liquid biopsy and fluorescence spectroscopy. Cancers. 2023;15(7):1952.
142. Hernández-López R, Hermosillo L, León-Maldonado L, Velázquez-Cruz R, Torres-Ibarra L, Lazcano-Ponce E, et al. Performance of an affordable urine self-sampling method for human papillomavirus detection in Mexican women. PLoS One. 2021;16(7):e0254946.
143. Hideura E, Suehiro Y, Nishikawa J, Shuto T, Fujimura H, Ito S, et al. Blood-free-circulating DNA testing of methylated RUNX3 is useful for diagnosing early gastric cancer. Cancers. 2020;12(4):789.
144. Hinestrosa JP, Sears RC, Dhani H, Lewis JM, Schroeder G, Balcer HI, et al. Development of a blood-based extracellular vesicle classifier for detection of early-stage pancreatic ductal adenocarcinoma. Commun Med. 2023;3(1):146.
145. Hinestrosa JP, Kurzrock R, Lewis JM, Schork NJ, Schroeder G, Kamat AM, et al. Early-stage multi-cancer detection using an extracellular vesicle protein-based blood test. Commun Med. 2022;2:29.
146. Hirschfeld M, Rücker G, Weib D, Berner K, Ritter A, Jäger M, et al. Urinary exosomal microRNAs as potential non-invasive biomarkers in breast cancer detection. Mol Diagn Ther. 2020;24(2):215–32.
147. Hitchins MP, Vogelaar IP, Brennan K, Haraldsdottir S, Zhou N, Martin B, et al. Methylated SEPTIN9 plasma test for colorectal cancer detection may be applicable to Lynch syndrome. BMJ Open Gastroenterol. 2019;6(1):e000299.
148. Ho H-L, Huang C-C, Ku W-H, Ho C-L, Lin C-H, Yu S-L, et al. Liquid biopsy for detection of EGFR T790M mutation in nonsmall cell lung cancer: An experience of proficiency testing in Taiwan. J Chin Med Assoc. 2019;82(6):473–76.
149. Horsfall LJ, Clarke CS, Nazareth I, Ambler G. The value of blood-based measures of liver function and urate in lung cancer risk prediction: A cohort study and health economic analysis. Cancer Epidemiol. 2023;84:102354.
150. Horstmann M, Banek S, Gakis G, Todenhöfer T, Aufderklamm S, Hennenlotter J, et al. Prospective evaluation of fluorescence-guided cystoscopy to detect bladder cancer in a high-risk population: results from the UroScreen-Study. Springerplus. 2014;3:24.
151. Hsu Y-C, Huang S-M, Chang L-C, Chen Y-M, Chang Y-H, Lin J-W, et al. Screening of early-staged colorectal neoplasia by clonal hematopoiesis-based liquid biopsy and machine-learning. Am J Cancer Res. 2022;12(3):1088–101.
152. Hu A, Zhang L, Wang Z, Yuan C, Lin L, Zhang J, et al. Cancer serum atlas-supported precise pan-targeted proteomics enable multicancer detection. Anal Chem. 2023;95(2):862–71.
153. Hu L, Du S, Guo W, Chen D, Li Y. Comparison of serum human epididymis protein 4 and carbohydrate antigen 125 as markers in endometrial cancer: A meta-analysis. Int J Gynecol Cancer. 2016;26(2):331–40.
154. Huang Z, Zhang X, Lu H, Wu L, Wang D, Zhang Q, et al. Serum trefoil factor 3 is a promising non-invasive biomarker for gastric cancer screening: A monocentric cohort study in China. BMC Gastroenterol. 2014;14:74.
155. Huerta M, Roselló S, Sabater L, Ferrer A, Tarazona N, Roda D, et al. Circulating tumor DNA detection by digital-droplet PCR in pancreatic ductal carcinoma: A systematic review. Cancers. 2021;13(5):994.
156. Hulstaert E, Morlion A, Levanon K, Vandesompele J, Mestdagh P. Candidate RNA biomarkers in biofluids for early diagnosis of ovarian cancer: A systematic review. Gynecol Oncol. 2021;160(2):633–42.
157. Ibáñez R, Mareque M, Granados R, Andía D, García-Rojo, Quílez JC, et al. Comparative cost analysis of cervical cancer screening programme based on molecular detection of HPV in Spain. BMC Womens Health. 2021;21(1):178.
158. Idrees M, Farah CS, Sloan P, Kujan O. Oral brush biopsy using liquid-based cytology is a reliable tool for oral cancer screening: A cost-utility analysis: Oral brush biopsy for oral cancer screening. Cancer Cytopathol. 2022;130(9):740–48.
159. Jacobs IJ, Menon U, Ryan A, Gentry-Maharaj A, Burnell M, Kalsi JK, et al. Ovarian cancer screening and mortality in the UK Collaborative Trial of Ovarian Cancer Screening (UKCTOCS): A randomised controlled trial. Lancet. 2016;387(10022):945–56.
160. Jain M, Kamalov D, Tivtikyan A, Balatsky A, Samokhodskaya L, Ohhobotov D, et al. Urine TERT promoter mutations-based tumor DNA detection in patients with bladder cancer: A pilot study. Mol Cin Oncol. 2021;15(6):253.
161. Jamshidi A, Liu MC, Klein EA, Venn O, Hubbell E, Beausang JF, et al. Evaluation of cell-free DNA approaches for multi-cancer early detection. Cancer Cell. 2022;40(12):1536–1549.e12.
162. Jensen SO, Øgaard N, Ørntoft M-VW, Rasmussen MH, Bramsen JB, Kristensen H, et al. Novel DNA methylation biomarkers show high sensitivity and specificity for blood-based detection of colorectal cancer-a clinical biomarker discovery and validation study. Clin Epigenetics. 2019;11(1):158.
163. Jiang D, Zhang X, Liu M, Wang Y, Wang T, Pei L, et al. Discovering panel of autoantibodies for early detection of lung cancer based on focused protein array. Front Immunol. 2021;12:658922.
164. Jiang H-H, Xing S-W, Tang X, Chen Y, Lin K, He L-W, et al. Novel multiplex stool-based assay for the detection of early-stage colon cancer in a Chinese population. World J Gastroenterol. 2022;28(24):2705–32.
165. Jiang L, Cheng Q, Zhang B-H, Zhang M-Z. Circulating microRNAs as biomarkers in hepatocellular carcinoma screening: A validation set from China. Medicine. 2015;94(10):e603.
166. Jiao B, Gulati R, Katki HA, Castle PE, Etzioni R. A quantitative framework to study potential benefits and harms of multi-cancer early detection testing. Cancer Epidemiol Biomarkers Prev. 2022;31(1):38–44.
167. Jo A, Green A, Medina JE, Iyer S, Ohman AW, McCarthy ET, et al. Inaugurating high-throughput profiling of extracellular vesicles for earlier ovarian cancer detection. Adv Sci. 2023;10(27):e2301930.
168. Jordaens S, Zwaenepoel K, Tjalma W, Deben C, Beyers K, Vankerchhoven V, et al. Urine biomarkers in cancer detection: A systematic review of preanalytical parameters and applied methods. Int J Cancer. 2023;152(10):2186–205.
169. Katz RL, Zaidi TM, Pujara D, Shanbhag ND, Truong D, Patil S, et al. Identification of circulating tumor cells using 4-color fluorescence in situ hybridization: Validation of a noninvasive aid for ruling out lung cancer in patients with low-dose computed tomography-detected lung nodules. Cancer Cytopathol. 2020;128(8):553–62.
170. Kayamba V, Kelly P. Introducing the sanguis-filum for detection of gastric mucosal lesions prior to endoscopy: A study protocol. Diagnostics. 2022;12(5):1160.
171. Kazarian A, Blyuss O, Metodieva G, Gentry-Maharaj A, Ryan A, Kiseleva EM, et al. Testing breast cancer serum biomarkers for early detection and prognosis in pre-diagnosis samples. Br J Cancer. 2017;116(4):501–08.
172. Khani M, Hosseini J, Mirfakhraie R, Habibi M, Azargashb E, Pouresmaeili F. The value of the plasma circulating cell-free DNA concentration and integrity index as a clinical tool for prostate cancer diagnosis: A prospective case-control cohort study in an Iranian population. Cancer Manag Res. 2019;11:4549–56.
173. Kim A, Chung KC, Keir C, Patrick DL. Patient-reported outcomes associated with cancer screening: A systematic review. BMC Cancer. 2022;22(1):223.
174. Kim AK, Hamilton JP, Lin SY, Chang T-T, Hann H-W, Hu C-T, et al. Urine DNA biomarkers for hepatocellular carcinoma screening. Br J Cancer. 2022;126(10):1432–38.
175. Kisiel JB, Dukek BA, Kanipakam RVSR, Ghoz HM, Yab TC, Beger CK, et al. Hepatocellular carcinoma detection by plasma methylated DNA: Discovery, phase I pilot, and phase II clinical validation. Hepatology. 2019;69(3):1180–92.
176. Klein EA, Partin A, Lotan Y, Baniel J, Dineen M, Hafron J, et al. Clinical validation of IsoPSA, a single parameter, structure-focused assay for improved detection of prostate cancer: A prospective, multicenter study. Urol Oncol. 2022;40(9):408.e9–408.e18.
177. Koga Y, Yamazaki N, Takizawa S, Kawauchi J, Nomura O, Yamamoto S, et al. Gene expression analysis using a highly sensitive DNA microarray for colorectal cancer screening. Anticancer Res. 2014;34(1):169–76.
178. Kotoh Y, Suehiro Y, Saeki I, Hoshida T, Maeda M, Iwamoto T, et al. Novel liquid biopsy test based on a sensitive methylated SEPT9 assay for diagnosis hepatocellular carcinoma. Hepatol Commun. 2020;4(3):461–70.
179. Kraus S, Shapira S, Kazanov D, Naumov I, Moshkowitz M, Santo E, et al. Predictive levels of CD_24_ in peripheral blood leukocytes for the early detection of colorectal adenomas and adenocarcinomas. Dis Markers. 2015;2015:916098.
180. Lacombe J, Mangé A, Bougnoux A-C, Prassas I, Solassol J. A multiparametric serum marker panel as a complementary test to mammography for the diagnosis of node-negative early-stage breast cancer and DCIS in young women. Cancer Epidemiol Biomarkers Prev. 2014;23(9):1834–42.
181. Lam WKJ, Jiang P, Chan KCA, Cheng SH, Zhang H, Peng W, et al. Sequencing-based counting and size profiling of plasma Epstein-Barr virus DNA enhance population screening of nasopharyngeal carcinoma. Proc Natl Acad Sci USA. 2018;115(22):E5115-E5124.
182. Lathean TJH, Wassie MM, Winter JM, Goyal R, Young GP, Symonds EL. Accuracy of blood-based biomarkers for screening precancerous colorectal lesions: A protocol for systematic review and meta-analysis. BMJ Open. 2022;12(5):e060712.
183. Laugsand EA, Brenne SS, Skorpen F. DNA methylation markers detected in blood, stool, urine, and tissue in colorectal cancer: A systematic review of paired samples. Int J Colorectal Dis. 2021;36(2):239–51.
184. Le Calvez-Kelm F, Foll M, Wozniak MB, Delhomme TM, Durand G, Chopard P, Pertesi M, et al. KRAS mutations in blood circulating cell-free DNA: A pancreatic cancer case-control. Oncotarget. 2016;7(48):78827–40.
185. Lee E-S, Kim Y, Shin H-C, Hwang K-T, Min J, Kim MK, et al. Diagnostic accuracy of a three-protein signature in women with suspicious breast lesions: A multicenter prospective trial. Breast Cancer Res. 2023;25(1):20.
186. Leng Q, Lin Y, Jiang F, Lee C-J, Zhan M, Fang HB, et al. A plasma miRNA signature for lung cancer early detection. Oncotarget. 2017;8(67):111902–11.
187. Leon Arellano M, García-Arranz M, Guadalajara H, Olivera-Salazar R, Valdes-Sanchez T, García-Olmo D. Analysis of Septin 9 gene hypermethylation as follow-up biomarker of colorectal cancer patients after curative surgery. Diagnostics. 2022;12(4):993.
188. Leung LL, Riaz Mk, Qu X, Chan J, Meehan K. Profiling of extracellular vesicles in oral cancer, from transcriptomics to proteomics. Semin Cancer Biol. 2021;74:3–23.
189. Li B, Pu K, Ge L, Wu X. Diagnostic significance assessment of the circulating cell-free DNA in ovarian cancer: An updated meta-analysis. Gene. 2019;714:143993.
190. Li P, Agbisit C, Noel M, Mukherji R. Evolving applications of liquid biopsies in gastrointestinal cancers. Clin Adv Hematol Oncol. 2024;22(1):43–54.
191. Li Q, Liu Y, Meng Z, Ge Q, Zhao L, Chu H, et al. Combination of serum test and questionnaire in early gastric cancer screening. Iran J Public Health. 2022;51(8):1817–26.
192. Li Y, Jiang G, Wu W, Yang H, Jin Y, Wu M, et al. Multi-omics integrated circulating cell-free DNA genomic signatures enhanced the diagnostic performance of early-stage lung cancer and postoperative minimal residual disease. EBioMedicine. 2023;91:104553.
193. Li Z, Bui DT, Shao Y, KItova EN, White S, Vesprini D, et al. Native mass spectrometry quantitation of alpha2-3-linked N-acetylneuraminic acid content of prostate-specific antigen: An accurate liquid biopsy for clinically significant prostate cancer. Anal Chem. 2023;95(29):10903–912.
194. Lin Y, Leng Q, Zhan M, Jiang F. A plasma long noncoding RNA signature for early detection of lung cancer. Transl Oncol. 2018;11(5):1225–31.
195. Liu C, Kannisto E, Yu G, Yang Y, Reid ME, Patnaik SK, et al. Non-invasive detection of exosomal microRNAs via tethered cationic lipoplex nanoparticles (tCLN) biochip for lung cancer early detection. Front Genet. 2020;11:258.
196. Liu J, Zhao H, Huang Y, Xu S, Zhou Y, Zhang W, et al. Genome-wide cell-free DNA methylation analyses improve accuracy of non-invasive diagnostic imaging for early-stage breast cancer. Mol Cancer. 2021;20(1):36.
197. Liu W, Ji T, Zhang C, Zhou Q, Bao Z. Cell-free DNA hypermethylated genes may have a limited role in cancer screening but a potential role in risk assessment of head and neck cancer. Oral Oncol. 2022;134:10629.
198. Löding S, Andersson U, Kaaks R, Schulze MB, Pala V, Urbarova I, et al. Altered plasma metabolite levels can be detected years before a glioma diagnosis. JCI Insight. 2023;8(19):e171225.
199. Logozzi M, Mizzoni D, Di Raimo R, Giuliani A, Maggi M, Sciarra A, et al. Plasmatic exosome number and size distinguish prostate cancer patients from healthy individuals: A prospective clinical study. Front Oncol. 2021;11:727317.
200. Lopes C, Chaves J, Ortigão R, Dinia-Ribeiro M, Pereira C. Gastric cancer detection by non-blood-based liquid biopsies: A systematic review looking into the last decade of research. United European Gastroenterol J. 2023;11(1):114–30.
201. Lou PJ, Lam WKJ, Hsu WL, Pfeiffer RM, Yu KJ, Chan CML, et al. Performance and operational feasibility of Epstein-Barr virus-based screening for detection of nasopharyngeal carcinoma: Direct comparison of two alternative approaches. J Clin Oncol. 2023;41(26):4257–66.
202. Lucien F, Lac V, Billadeau DD, Borgida A, Gallinger S, Leong HS. Glypican-1 and glycoprotein 2 bearing extracellular vesicles do not discern pancreatic cancer from benign pancreatic diseases. Oncotarget. 2019;10(10):1045–55.
203. Luo X, Wu Y, Ji M, Zhang S. Combined plasma microRNA and fecal occult blood tests in early detection of colorectal cancer. Clin Lab. 2019;65(5).
204. Maccini MA, Westfall NJ, Van Bokhoven A, Lucia MS, Poage W, Maroni PD, et al. The effect of digital rectal exam on the 4Kscore for aggressive prostate cancer. Prostate. 2018;78(7):506–11.
205. Martel A, Mograbi B, Romeo B, Gastaud L, Lalvee S, Zahaf K, et al. Assessment of different circulating tumor cell platforms for uveal melanoma: Potential impact for future routine clinical practice. Int J Mol Sci. 2023;24(13):11075.
206. Martignetti JA, Pandya D, Nagarsheth N, Chen Y, Camacho O, Tomita S, et al. Detection of endometrial precancer by a targeted gynecologic cancer liquid biopsy. Cold Spring Harb Mol Case Stud. 2018;4(6):a003269.
207. Martin FL, Morais CLM. Dickinson AW, Saba T, Bongers T, Singh MN, et al. Point-of-care disease screening in primary care using saliva: A biospectroscopy approach for lung cancer and prostate cancer. J Pers Med. 2023;13(11):1533.
208. Mendez K, Romaguera J, Ortiz AP, López M, Steinau M, Unger ER. Urine-based human papillomavirus DNA testing as a screening tool for cervical cancer in high-risk women. Int J Gynaecol Obstet. 2014;124(2):151–5.
209. Meng H, Yao W, Nan M-M, Zhou G-G, Song W-Y, Li Y-Z, et al. The diagnostic value of circulating abnormal cells in early lung cancer. Am J Cancer Res. 2023;13(4):1594–1601.
210. Menon U, Gentry-Maharaj A, Burnell M, Ryan A, Kalsi JK, Singh N, Dawnay A, et al. Mortality impact, risks, and benefits of general population screening for ovarian cancer: the UKCTOCS randomised controlled trial. Health Technol Assess. 2023:1–81.
211. Metzenmacher M, Hegedüs B, Forster J, Schramm A, Horn PA, Klein CA, et al. Combined multimodal ctDNA analysis and radiological imaging for tumor surveillance in non-small cell lung cancer. Transl Oncol. 2022;15(1):101279.
212. Miao J, Liu Y, Zhao G, Liu X, Ma Y, Li H, et al. Feasibility of plasma-methylated SFRP2 for early detection of gastric cancer. Cancer Control. 2020;27(2):1073274820922559.
213. Mijnes J, Tiedemann J, Eschenbruch J, Gasthaus J, Bringezu S, Bauerschlag D, et al. SNiPER: a novel hypermethylation biomarker panel for liquid biopsy based early breast cancer detection. Oncotarget. 2019;10(60):6494–508.
214. Min L, Chen J, Yu M, Liu D. Using circulating tumor DNA as a novel biomarker to screen and diagnose colorectal cancer: A meta-analysis. J Clin Med. 2023;12(2):408.
215. Mlika M, Dziri C, Zorgati MM, Khelil MB, Mezni F. Liquid biopsy as a surrogate to tissue in lung cancer for molecular profiling: A meta-analysis. Curr Respir Med Rev. 2018;14(1):48–60.
216. Molnár B, Tóth K, Barták BK, Tulassay Z. Plasma methylated septin 9: a colorectal cancer screening marker. Expert Rev Mol Diagn. 2015;15(2):171–84.
217. Moon SM, Kim J-H, Kim SK, Kim S, Kwon H-J, Bae J-S, et al. Clinical utility of combined circulating tumor cell and circulating tumor DNA assays for diagnosis of primary lung cancer. Anticancer Res. 2020;40(6):3435–44.
218. Moretti F, D;Antona P, Finardi E, Barbetta M, Diominioni L, Poli A, et al. Systematic review and critique of circulating miRNAs as biomarkers of stage I-II non-small cell lung cancer. Oncotarget. 2017;8(55):94980–96.
219. Morris S, Vachani A, Pass HI, Rom WN, Ryden K, Weiss GJ, et al. Whole blood FPR1 mRNA expression predicts both non-small cell and small cell lung cancer. Int J Cancer. 2018;142(11):2355–62.
220. Moshayoff V, Faktor O, Laghi L, Celesti G, Peretz T, Keret D, et al. Feasibility of unbiased RNA profiling of colorectal tumors: A proof of principle. PLoS One. 2016;11(7):e0159522.
221. Nené NR, Ney A, Nazarenko T, Blyuss O, Johnston HE, Whitwell HJ, et al. Serum biomarker-based early detection of pancreatic ductal adenocarcinomas with ensemble learning. Commun Med (Lond). 2023;3(1):10.
222. Ng L, Wan TM-H, Man JH-W, Chow AK-M, Iyer D, Chen G, et al. Identification of serum miR-139-3p as a non-invasive biomarker for colorectal cancer. Oncotarget. 2017;8(16):27393–400.
223. Nguyen HT, Huynh LAK, Nguyen TV, Tran DH, Tran TTT, Le NDK, et al. Multimodal analysis of ctDNA methylation and fragmentomic profiles enhances detection of nonmetastatic colorectal cancer. Future Oncol. 2022;18(35):3895–912.
224. Nilyanimit P, Chansaenroj J, Karalak A, Laowahutanont P, Junyangdikul P, Poovorawan Y. Comparison of human papillomavirus (HPV) detection in urine and cervical swab samples using the HPV GenoArray Diagnostic assay. PeerJ. 2017;5:e3910.
225. Ning W, Wu T, Wu C, Wang S, Tao Z, Wang G, et al. Accurate prediction of pan-cancer types using machine learning with minimal number of DNA methylation sites. J Mol Cell Biol. 2023;15(4):mjad023.
226. Niu Y, Xue J, Wu X, Qu M, Wang L, Liang W, et al. Clinical significance of serum haptoglobin and protein disulfide-isomerase A3 in the screening, diagnosis, and staging of colorectal cancer. Front Pharmacol. 2022;13:935500.
227. Njoku K, Campbell AE, Geary B, MacKintosh ML, Derbyshire AE, Kitson SJ, et al. Metablomic biomarkers for the detection of obesity-driven endometrial cancer. Cancers. 2021;13(4):718.
228. Nordströ m T, Jäderling F, Carlsson S, Aly M, Gronönberg H, Eklund M. Does a novel diagnostic pathway including blood-based risk prediction and MRI-targeted biopsies outperform prostate cancer screening using prostate-specific antigen and systematic prostate biopsies? Protocol of the randomised study STHLM3MRI. BMJ Open. 2019;9(6):e027816.
229. Okubo R, Hoshi S-L, Kimura T, Kondo M, Asahi K, Iseki C, et al. Cost-effectiveness of mass screening for dipstick hematuria in Japan. Clin Exp Nephrol. 2022;26(5):398–412.
230. Otsu H, Nambara S, Hu Q, Hisamatsu Y, Toshima T, Takeishi K, et al. Identification of serum microRNAs as potential diagnostic biomarkers for detecting precancerous lesions of gastric cancer. Ann Gastroenterol Surg. 2022;7(1):63–70.
231. Owens L, Gulati R, Etzioni R. Stage shift as an endpoint in cancer screening trials: Implications for evaluating multicancer early detection tests. Cancer Epidemiol Biomarks Prev. 2022;31(7):1298–304.
232. Leitão TP, Miranda M, Polido J, Morais J, Corredeira P, Alves P, et al. Circulating tumor cell detection methods in renal cell carcinoma: A systematic review. Crit Rev Oncol Hematol. 2021;161:103331.
233. Pascual J, Attard G, Bidard F-C, Curigliano G, De Mattos-Arruda L, Diehn M, et al. ESMO recommendations on the use of circulating tumour DNA assays for patients with cancer: a report from the ESMO Precision Medicine Working Group. Ann Oncol. 2022;33(8):750–68.
234. Paz-Elizur T, Leitner-Dagan Y, Meyer KB, Markus B, Giorgi FM, O’Reilly M, et al. DNA repair biomarker for lung cancer risk and its correlation with airway cells gene expression. JNCI Cancer Spectr. 2019;4(1):pkz067.
235. Pedersen JW, Gentry-Maharaj A, Nøstdal A, Fourkala E-O, Dawnay A, Burnell M, et al. Cancer-associated autoantibodies to MUC1 and MUC4 – A blinded case-control study of colorectal cancer in UK collaborative trial of ovarian cancer screening. Int J Cancer. 2014;134(9):180–88.
236. Pedersen SK, Baker RT, McEvoy A, Murray DH, Thomas M, Molloy PL, et al. A two-gene blood test for methylated DNA sensitive for colorectal cancer. PLos One. 2015;10(4):e0125041.
237. Perakis S, Auer M, Belic J, Heitzer E. Advances in circulating tumor DNA analysis. Adv Clin Chem. 2017;80:73–153.
238. Pereira C, Areia M, Dinis-Ribeiro M. Cost-utility analysis of genetic polymorphism universal screening in colorectal cancer prevention by detection of high-risk individuals. Dig Liver Dis. 2019;51(12):1731–37.
239. Perrone F, Lampis A, Bertan C, Verderio P, Ciniselli CM, Pizzamiglio S, et al. Circulating free DNA in a screening program for early colorectal cancer detection. Tumori. 2014;100(2):115–21.
240. Petersen MM, Kleif J, Jørgensen LN, Hendel JW, Seidelin JB, Madsen MR, et al. Optimizing screening for colorectal cancer: An algorithm combining fecal immunochemical test, blood-based cancer-associated proteins and demographics to reduce colonoscopy burden. Clin Colorectal Cancer. 2023;22(2):199–210.
241. Phelps R, Gallon R, Hayes C, Glover E, Gibson P, Edidi I, et al. Detection of microsatellite instability in colonoscopic biopsies and postal urine samples from Lynch syndrome cancer patients using a multiplex PCR assay. Cancers. 2022;14(15):3838.
242. Pilyugin M, Ratajska M, Stukan M, Concin N, Zeillinger R, Irminger-Finger I. BARD_1_ autoantibody blood test for early detection of ovarian cancer. Genes. 2021;12(7):969.
243. Qiao G, Zhuang W, Dong B, Li C, Xu J, Wang G, et al. Discovery and validation of methylation signatures in circulating cell-free DNA for early detection of esophageal cancer: A case-control study. BMC Med. 2021;19(1):243.
244. Rahaman S, Li X, Yu J, Wong K-C. CancerEMC: frontline non-invasive cancer screening from circulating protein biomarkers and mutations in cell-free DNA. Bioinformatics. 2021;37(19):3319–27.
245. Rahier J-F, Druez A, Faugeras L, Martinet J-P, Géhénot M, Josseaux E, et al. Circulating nucleosomes as new blood-based biomarkers for detection of colorectal cancer. Clin Epigenetics. 2017;9:53.
246. Rani S, Sehgal A, Kaur J, Pandher DK, Punia RS. Osteopontin as a tumor marker in ovarian cancer. J Midlife Health. 2022;13(3):200–05.
247. Rapado-González O, Majem B, Álvarez-Castro, Díaz-Peña R, Abalo A, Suárez-Cabrera L, et al. A novel saliva-based miRNA signature for colorectal cancer diagnosis. J Clin Med. 2019;8(12):2029.
248. Rasmussen L, Nielsen HJ, Christensen IJ. Early detection and recurrence of colorectal adenomas by combination of either cancer-associated biomarkers in plasma. Clin Exp Gastroenterol. 2020;13:273–84.
249. Ravegnini G, Moraga JMZ, Maffei F, Musti M, Zenesini C, Simeon V, et al. Simultaneous analysis of SEPT9 promoter methylation status, micronuclei frequency, and folate-related gene polymorphisms: The potential for a novel blood-based colorectal cancer biomarker. Int J Mol Sci. 2015;16(12):28486–97.
250. Ravera F, Cirmena G, Dameri M, Gallo M, Vellone VG, Fregatti P, et al. Development of a hoRizontal data intEgration classifier for NOn-invasive early diAgnosis of breasT cancEr: the RENOVATE study protocol. BMJ Open. 2021;11(12):e054256.
251. Rezhake R, Wang Y, Chen F, Hu SY, Zhang X, Cao J, et al. Clinical evaluation of p16^INK4a^ immunocytology in cervical cancer screening: A population-based cross-sectional study from rural China. Cancer Cytopathol. 2021;129(9):679–92.
252. Rodia MT, Ugolini G, Mattei G, Montroni I, Zattoni D, Ghignone F, et al. Systematic large-scale meta-analysis identifies a panel of two mRNAs as blood biomarkers for colorectal cancer detection. Oncotarget. 2016;7(21):30295–306.
253. Rodrigo A, Ojeda JL, Vega S, Sanchez-Gracia O, Lanas A, Isla D, et al. Thermal liquid biopsy (TLB): A predictive score derived from serum thermograms as a clinical tool for screening lung cancer patients. Cancers. 2019;11(7):1012.
254. Rui T, Wang K, Xiang A, Guo J, Tang N, Jin X, et al. Serum exosome-derived piRNAs could be promising biomarkers for HCC diagnosis. Int J Nanomedicine. 2023;18:1989–2001.
255. Sala A, Cameron JM, Jenkins CA, Barr H, Christie L, Conn JJA, et al. Liquid biopsy for pancreatic cancer detection using infrared spectroscopy. Cancers. 2022;14(13):3048.
256. Salfer B, Havo D, Kuppinger S, Wong DTW, Li F, Zhang L. Evaluating pre-analytical variables for saliva cell-free DNA liquid biopsy. Diagnostics. 2023;13(10):1665.
257. Saura C, Ortiz C, Matito J, Arenas EJ, Suñol A, Martín Á, et al. Early-stage breast cancer detection in breast milk. Cancer Discov. 2023;13(10):2180–91.
258. Sayal L, Hamadah O, Almasri A, Idrees M, Thomson P, Kujan O. Saliva-based cell-free DNA and cell-free mitochondrial DNA in head and neck cancers have promising screening and early detection role. J Oral Pathol Med. 2023;52(1):29–36.
259. Scelo G, Muller DC, Riboli E, Johansson M, Cross AJ, Vineis P, et al. KIM-1 as a blood-based marker for early detection of kidney cancer: A prospective nested case-control study. Clin Cancer Res. 2018;24(22):5594–601.
260. Schröck A, Leisse A, de Vos L, Gevensleben H, Dröge F, Franzen A, et al. Free-circulating methylated DNA in blood for diagnosis, staging, prognosis, and monitoring of health and neck squamous cell carcinoma patients: An observational prospective cohort study. Clin Chem. 2017;63(7):1288–96.
261. Schwartzberg L, Broder MS, Ailawadhi S, Beltran H, Blakely LJ, Budd GT, et al. Impact of early detection on cancer curability: A modified Delphi panel study. PLoS One. 2022;17(12):e0279227.
262. Sekacheva M, Boroda A, Fatyanova A, Rozhkov A, Bagmet N. Clinical validation of the novel CLIA-CA-62 assay efficacy for early-stage breast cancer detection. Front Oncol. 2023;13:1009863.
263. Shah R, Jones E, Vidart V, Kuppen PJK, Conti JA, Francis NK. Biomarkers for early detection of colorectal cancer and polyps: systematic review. Cancer Epidemiol Biomarkers Prev. 2014;23(9):1712–28.
264. Shapira S, Kazanov D, Mdah F, Yaakobi H, Herishanu Y, Perry C, et al. Feasibility of CD_24_/CD_11b_ as a screening test for hematological malignancies, J Pers Med. 2021;11(8):724.
265. Sharma N, Yadav M, Tripathi G, Mathew B, Bindal V, Falari S, et al. Bile multi-omics analysis classifies lipid species and microbial peptides predictive of carcinoma of gallbladder. Hepatology. 2022;76(4):920–35.
266. Sheng M, Dong Z, Xie Y. Identification of tumor-educated platelet biomarkers of non-small-cell lung cancer. Onco Targets Ther. 2018;11:8143–51.
267. Shi J, Cheng C, Ma J, Liew C-C, Geng X. Gene expression signature for detection of gastric cancer in peripheral blood. Oncol Lett. 2018;15(6):9802–10.
268. Singer G, Ramakrishnan VM, Rogel U, Schötzau A, Disteldorf D, Maletzki P, et al. The role of new technologies in the diagnosis and surveillance of non-muscle invasive bladder carcinoma: A prospective, double-blinded, monocentric study of the XPERT© bladder cancer monitor and narrow band imaging© cystoscopy. Cancers. 2022;14(3):618.
269. Singh A, Gupta S, Sachan M. Evaluation of the diagnostic potential of candidate hypermethylated genes in epithelial ovarian cancer in North Indian population. Front Mol Biosci. 2021;8:719056.
270. Singh A, Gupta S, Badarukhiya JA, Sachan M. Detection of aberrant methylation of HOXA9 and HIC1 through multiplex MethyLight assay in serum DNA for the early detection of epithelial ovarian cancer. Int J Cancer. 2020;147(6):1740–52.
271. Søiland H, Janssen EAM, Helland T, Eliassen FM, Hagland M, Nordgård O, et al. Liquid biopsies and patient-reported outcome measures for integrative monitoring of patients with early-stage breast cancer: a study protocol for the longitudinal observational Prospective Breast Cancer Biobanking (PBCB) study. BMJ Open. 2022;12(4):e054404.
272. Srivastava P, Mishra S, Agarwal A, Pandey A, Husain N. Circulating microRNAs in gallbladder cancer: Is serum assay of diagnostic value? Pathol Res Pract. 2023;242:154320.
273. Su Z, Zhao J, Ke S, Zhang J, Liu X, Wang Y, et al. Clinical significance of circulating tumor cells via combined whole exome sequencing in early stage cancer screening: A case report. Exp Ther Med. 2018;16(3):2527–33.
274. Suehiro Y, Hashimoto S, Higaki S, Fujii I, Suzuki C, Hoshida T, et al. Blood free-circulating DNA testing by highly sensitive methylation assay to diagnose colorectal neoplasias. Oncotarget. 2018;9(24):16974–87.
275. Sun Y, Liu S, Qiao Z, Shang Z, Xia Z, Niu X, et al. Systematic comparison of exosomal proteomes from human saliva and serum for the detection of lung cancer. Anal Chim Acta. 2017;982:84–95.
276. Symonds EL, Cock C, meng R, Cole SR, Fraser RJL, Young GP. Uptake of a colorectal cancer screening blood test in people with elevated risk for cancer who cannot or will not complete a faecal occult blood test. Eur J Cancer Prev. 2018;27(5):425–32.
277. Tan D, Jiang W, Hu R, Li Z, Ou T. Detection of the ADGRG6 hotspot mutations in urine for bladder cancer early screening by ARMS-qPCR. Cancer Med. 2023;12(10):1503–12.
278. Tanzi E, Bianchi S, Fappani C, Gori M, Colzani D, Passera I, et al. Detection of human papillomavirus in fresh and dried urine through an automated system for cervical cancer screening in low- and middle-income countries. J Med Virol. 2023;95(5):e28802.
279. Tcherkassova J, Prostyakova A, Tsurkan S, Ragoulin V, Boroda A, Sekacheva M. Diagnostic efficacy of the new prospective biomarker's combination CA 15-3 and CA-62 for early-stage breast cancer detection: Results of the blind prospective-retrospective clinical study. Cancer Biomark. 2022;35(1):57–69.
280. Terp SK, Stoico MP, Dybkaer K, Pedersen IS. Early diagnosis of ovarian cancer based on methylation profiles in peripheral blood cell-free DNA: A systematic review. Clin Epigenetics. 2023;15(1):24.
281. Theakstone AG, Brennan PM, Jenkinson MD, Mills SJ, Syed K, Rinaldi C, et al. Rapid spectroscopic liquid biopsy for the universal detection of brain tumors. Cancers. 2021;13(15):3851.
282. Tie J. Triaging suspected cancer with a multi-cancer early detection blood test. Lancet Oncol. 2023;24(7):710–11.
283. Tognela A, Spring KJ, Becker T, Caixeiro NJ, Bray VJ, Yip PY, et al. Predictive and prognostic value of circulating tumor cell detection in lung cancer: a clinician's perspective. Crit Rev Oncol Hematol. 2015;93(2):90–102.
284. Toiyama Y, Okugawa Y, Fleshman J, Boland CR, Goel A. MicroRNAs as potential liquid biopsy biomarkers in colorectal cancer: A systematic review. Biochim Biophys Acta Rev Cancer. 2018;1870(2):274–82.
285. Tsai MH, Wang HC, Lee GW, Lin YC, Chiu SH. A decision tree based classifier to analyze human ovarian cancer cDNA microarray datasets. J Med Syst. 2016;40(1):21.
286. Tshomo U, Franceschi S, Tshokey T, Tobgay T, Baussano I, Tenet V, et al. Evaluation of the performance of Human Papillomavirus testing in paired urine and clinician-collected cervical samples among women aged over 30 years in Bhutan. Virol J. 2017;14(1):74.
287. Tuo L, Sha A, Huayu Z, Du K. P16INK4a gene promoter methylation as a biomarker for the diagnosis of non-small cell lung cancer: An updated meta-analysis. Thorac cancer. 2018;9(8):1032–40.
288. Urabe F, Matsuzaki J, Yamamoto Y, Kimura T, Hara T, Ichikawa M, et al. Large-scale circulating microRNA profiling for the liquid biopsy of prostate cancer. Clin Cancer Res. 2019;25(10):3016–25.
289. Urban SK, Sänger H, Krawczyk M, Julich-Haertel H, Willms A, Ligocka J, et al. Synergistic effects of extracellular vesicle phenotyping and AFP in hepatobiliary cancer differentiation. Liver Int. 2020;40(12):3103–16.
290. Uttley L, Whiteman BL, Woods HB, Harnan S, Philips ST, Cree IA, et al. Building the evidence base of blood-based biomarkers for early detection of cancer: A rapid systematic mapping review. EBioMedicine. 2016;10:164–73.
291. van den Helder R, Wever BMM, van Trommel NE, van Splunter AP, Mom CH, Kasius JC, et al. Non-invasive detection of endometrial cancer by DNA methylation analysis in urine. Clin Epigenetics. 2020;12(1):165.
292. Van Keer S, Tjalma WAA, Pattyn J, Biesmans S, Pieters Z, Van Ostade X, et al. Human papillomavirus genotype and viral load agreement between paired first-void urine and clinician-collected cervical samples. Eur J Clin Microbiol Infect Dis. 2018;37(5):859–69.
293. Van Rees JM, Wullaert L, Grüter AAJ, Derraze Y, Tanis PJ, Verheul HMW, et al. Circulating tumour DNA as biomarker for rectal cancer: A systematic review and meta-analyses. Front Oncol. 2023;13:1083285.
294. Virdee PS, Marian IR, Mansouri A, Elhussein L, Kirtley S, Holt T, et al. The full blood count blood test for colorectal cancer detection: A systematic review, meta-analysis, and critical appraisal. Cancers. 2020;12(9):2348.
295. Virdee PS, Bankhead C, Koshiaris C, Drakesmith CW, Oke J, Withrow D, et al. BLOod Test Trend for cancEr Detection (BLOTTED): protocol for an observational and prediction model development study using English primary care electronic health record data. Diagn Progn Res. 2023; 7(1):1.
296. Virdee PS, Kirtley S, Elhussein L, Watkinson PJ, Holt TA, Birks J. Components of the full blood count as risk factors for colorectal cancer detection: A systematic review protocol. BMJ Open. 2019;9(12):e032759.
297. Virgilio E, Montali F, Annicchiarico A, Salvemini C, Baldinu M, Giarnieri E, et al. Exosomal functional cargoes from liquid biopsy of gastric cancer: A systematic review of studies with potential clinical relevance. Anticancer Res. 2022;42(5):2249–59.
298. Voigt JD, Dong Y, Linder V, Zappala S. Use of the 4Kscore test to predict the risk of aggressive prostate cancer prior to prostate biopsy: Overall cost savings and improved quality of care to the us healthcare system. Rev Urol. 2017;19(1): 1–10.
299. Wallace E, Higuchi R, Satya M, McCann L, Sin MLY, Bridge JA, et al. Development of a 90-minute integrated noninvasive urinary assay for bladder cancer detection. J Urol. 2018;199(3):655–62.
300. Wang F, Li X, Li M, Liu W, Lu L, Li Y, et al. Ultra-short cell-free DNA fragments enhance cancer early detection in a multi-analyte blood test combining mutation, protein and fragmentomics. Clin Chem Lab Med. 2023;62(1):168–77.
301. Wang H, Zhou Z, Li H, Xiang W, Lan Y, Dou X, et al. Blood biomarkers panels for screening of colorectal cancer and adenoma on a machine learning-assisted detection platform. Cancer Control. 2023;30:10732748231222109.
302. Wang H, Tso V, Wong C, Sadowski D, Fedorak RN. Development and validation of a highly sensitive urine-based test to identify patients with colonic adenomatous polyps. Clin Transl Gastroenterol. 2014;5(3):e54.
303. Wang M, Wang Y, Tian X, Wang Q, Huang H, Lu X, et al. Diagnostic and predictive value of liquid biopsy-derived exosome miR-21 for breast cancer: a systematic review and meta-analysis. Expert Rev Mol Diagn. 2023;23(4):315–24.
304. Wang M, Hao C, Ma Q, Song G, Ma S, Zhao D, et al. DNA image cytometry test for primary screening of esophageal cancer: a population-based multi-center study in high-risk areas in China. Chin J Cancer Res. 2016;28(4):404–12.
305. Wang T, Li P, Qi Q, Zhang S, Xie Y, Wang J, ey al. A multiplex blood-based assay targeting DNA methylation in PBMCs enables early detection of breast cancer. Nat Commun. 2023;14(1):4724.
306. Wang Z, Xie K, Zhu G, Ma C, Cheng C, Li Y, et al. Early detection and stratification of lung cancer aided by a cost-effective assay targeting circulating tumor DNA (ctDNA) methylation. Respir Res. 2023;24(1):163.
307. Weber DG, Casjens S, Wichert K, Lehnert M, Taeger D, Rihs HP, et al. Tasks and experiences of the prospective, longitudinal, multicenter MoMar (molecular markers) study for the early detection of mesothelioma in individuals formerly exposed for asbestos using liquid biopsies. Cancers. 2023;15(24):5896.
308. Weber DG, Brik A, Casjens S, Burek K, Lehnert M, Pesch B, et al. Are circulating microRNAs suitable for the early detection of malignant mesothelioma? Results from a nested case-control study. BMC Res Notes. 2019;12(1):77.
309. Wei B, Wu F, Xing W, Sun H, Yan C, Zhao C, et al. A panel of DNA methylation biomarkers for detection and improving diagnostic efficiency of lung cancer. Sci Rep. 2021;11(1):16782.
310. Weight RM, Viator JA. Detection of circulating tumor cells by photoacoustic flowmetry. Methods Mol Biol. 2014;1102:655–63.
311. Wen Y, Han J, Chen J, Dong J, Xia Y, Liu J, et al. Plasma miRNAs as early biomarkers for detecting hepatocellular carcinoma. Int J Cancer. 2015;137(7):1679–90.
312. Widschwendter M, Zikan M, Wahl B, Lempiäinen H, Paprotka T, Evans I, et al. The potential of circulating tumor DNA methylation analysis for the early detection and management of ovarian cancer. Genome Med. 2017;9(1):116.
313. Wielscher M, Vierlinger K, Kegler U, Ziesche R, Gsur A, Weinhäusel A. Diagnostic performance of plasma DNA methylation profiles in lung cancer, pulmonary fibrosis and COPD. EBioMedicine. 2015;2(8):929–36.
314. Wong D, Luo P, Oldfield LE, Gong H, Brunga L, Rabinowicz R, et al. Early cancer detection in Li-Fraumeni syndrome with cell-free DNA. Cancer Discov. 2024;14(1):104–19.
315. Wu CF, Lin L, Mao YP, Deng B, Lv JW, Zheng HW, et al. Liquid biopsy posttreatment surveillance in endemic nasopharyngeal carcinoma: A cost-effective strategy to integrate circulating cell-free Epstein-Barr virus DNA. BMC Med. 2021;19(1):193.
316. Wu H, Guo S, Liu X, Li Y, Su Z, He Q, et al. Noninvasive detection of pancreatic ductal adenocarcinoma using the methylation signature of circulating tumour DNA. BMC Med. 2022;20(1):458.
317. Wu J, Li L, Jiang C. Identification and evaluation of serum micro-RNA-29 family for glioma screening. Mol Neurobiol. 2015;52(3):1540–46.
318. Wu WYY, Haider Z, Feng X, Heath AK, Tjønneland A, Agudo A, et al. Assessment of the EarlyCDT-Lung test as an early biomarker of lung cancer in ever-smokers: A retrospective nested case-control study in two prospective cohorts. Int J Cancer. 2023;153(9):2002–10.
319. Wu X, Tanaka H. Aberrant reduction of telomere repetitive sequences in plasma cell-free DNA for early breast cancer detection. Oncotarget. 2015;6(30):29795–807.
320. Xie L, Du X, Wang S, Shi P, Qian Y, Zhang W, et al. Development and evaluation of cancer differentiation analysis technology: A novel biophysics-based cancer screening method. Expert Rev Mol Diagn. 2022;22(1):111–17.
321. Xing X, Cai L, Ouyang J, Wang F, Li Z, Liu M, et al. Proteomics-driven noninvasive screening of circulating serum protein panels for the early diagnosis of hepatocellular carcinoma. Nat Commun. 2023;14(1):8392.
322. Xu H, Yu Y, George W, Smith JS, Hu S, Dang L, et al. Comparison of the performance of paired urine and cervical samples for cervical cancer screening in screening population. J Med Virol. 2020;92(2):234–40.
323. Xu J, Song J, Wang T, Zhu W, Zuo L, Wu J, et al. A combination of methylation and protein markers is capable of detecting gastric cancer detection by combined marker. Epigenomics. 2021:13(19):1557–70.
324. Xu Y, Kim YH, Jeong P, Piao XM, Byun YJ, Kang HW, et al. Diagnostic value of combined IQGAP3/BMP4 and IQGAP3/FAM107A expression ratios in urinary cell-free DNA for discriminating bladder cancer from hematuria. Urol Oncol. 2019;37(1):86–96.
325. Xue M, Lai SC, Xu ZP, Wang LJ. Noninvasive DNA methylation biomarkers in colorectal cancer: A systematic review. J Dig Dis. 2015;16(12):699–712.
326. Yamada A, Horimatsu T, Okugawa Y, Nishida N, Honjo H, Ida H, et al. Serum miR-21, miR-29a, and miR-125b are promising biomarkers for the early detection of colorectal neoplasia. Clin Cancer Res. 2015;21(18):4234–42.
327. Yamazaki H, Wada T, Asano H, Fujita H, Okamoto K, Warari H. Comparison between urine and cervical high-risk HPV tests for Japanese women with ASC-US. Diagnostics. 2021;11(10):1895.
328. Yan S, Liu Z, Yu S, Bao Y. Diagnostic value of methylated septin9 for colorectal cancer screening: A meta-analysis. Med Sci Monit. 2016;22:3409–18.
329. Yang G, Wei L, Thong BKS, Fu Y, Cheong IH, Kozlakidis Z, et al. A systematic review of oral biopsies, sample types, and detection techniques applies in relation to oral cancer detection. BioTech. 2022;11(1):5.
330. Yang J, Barkley JE, Bhattari B, Firouzi K, Monk BJ, Coonrod DV, et al. Identification of endometrial cancer-specific microRNA biomarkers in endometrial fluid. Int J Mol Sci. 2023;24(10):8683.
331. Yang S, Che SPY, Kurywchak P, Tavormina JL, Gansmo LB, de Sampaio PC, et al. Detection of mutant KRAS and TP53 DNA in circulating exosomes from healthy individuals and patients with pancreatic cancer. Cancer Biol Ther. 2017;18(3):158–165.
332. Yang X, Suo C, Zhang T, Yin X, Man J, Yuan Z, et al. Targeted proteomics-derived biomarker profile develops a multi-protein classifier in liquid biopsies for early detection of esophageal squamous cell carcinoma from a population-based case-control study. Biomark Res. 2021;9(1):12.
333. Yang X, Dai W, Kwong DL, Szeto CYY, Wong EH, NG WT, et al. Epigenetic markers for noninvasive early detection of nasopharyngeal carcinoma by methylation-sensitive high resolution melting. Int J Cancer. 2015;136(4):E127–35.
334. Yin C, Luo C, Hu W, Ding X, Yuan C, Wang F. Quantitative and qualitative analysis of circulating cell-free DNA can be used as an adjuvant tool for prostate cancer screening: A meta-analysis. Dis Markers. 2026;2016:3825819.
335. Ying L, Du L, Zou R, Shi L, Zhang N, Jin J, et al. Development of a serum miRNA panel for detection of early stage non-small cell lung cancer. Proc Natl Acad Sci USA. 2020;117(40):25036–42.
336. Yokoi A, Yoshioka Y, Hirakawa A, Yamamoto Y, Ishikawa M, Ikeda SI, et al. A combination of circulating miRNAs for the early detection of ovarian cancer. Oncotarget. 2017;8(52):89811–23.
337. Yoshioka Y, Shimomura M, Saito K, Ishii H, Doki Y, Eguchi H, et al. Circulating cancer-associated extracellular vesicles as early detection and recurrence biomarkers for pancreatic cancer. Cancer Sci. 2022;113(10):3498–509.
338. Yu B, Zhou S, Liang H, Ye Q, Wang Y. Development and validation of a novel circulating miRNA-based diagnostic score for early detection of hepatocellular carcinoma. Dig Dis Sci. 2022;67(6):2283–92.
339. Yuan F, Li Z, Chen L, Zeng T, Zhang YH, Ding S, et al. Identifying the signatures and rules of circulating extracellular microRNA for distinguishing cancer subtypes. Front Genet. 2021;12:651610.
340. Zekri AR, Youssef ASED, Bakr YM, Gabr RM, El-Rouby MNED, Hammad I, et al. Serum biomarkers for early detection of hepatocellular carcinoma associated with HCV infection in Egyptian patients. Asian Pac J Cancer Prev. 2015;16(3):1281–87.
341. Zeller G, Tap J, Voigt AY, Sunagawa S, Kultima JR, Costea PI, et al. Potential of fecal microbiota for early-stage detection of colorectal cancer. Mol Syst Biol. 2014;10(11):766.
342. Zhang G, He F, Zhao G, Huang Z, Li X, Xia X, et al. Combining serum DNA methylation biomarkers and protein tumor markers improved clinical sensitivity for early detection of colorectal cancer. Int J Genomics. 2021;2021:6613987.
343. Zhang H, Zhao L, Jiang J, Zheng J, Yang L, Li Y, et al. Multiplexed nanomaterial-assisted laser desorption/ionization for pan-cancer diagnosis and classification. Nat Commun. 2022;13(1):617.
344. Zhang J, Tian X, Chen Y, Huang S, Cui Z, Tian R, et al. Feasibility and accuracy of menstrual blood testing for high-risk human papillomavirus detection with capture sequencing. JAMA Netw Open. 2021;4(12):e2140644.
345. Zhang S, Xie Y, Yu X, Ge J, Ye G, Guo J. Absolute quantification of a plasma tRNA-derived fragment for the diagnosis and prognosis of gastric cancer. Front Oncol. 2023;13:1106997.
346. Zhang S, Su M, Sun Z, Lu H, Zhang Y. The signature of pharmaceutical sensitivity based on ctDNA mutation in eleven cancers. Exp Biol Med. 2020;245(8):720–32.
347. Zhao A, Guo L, Xu J, Zheng L, Guo Z, Ling Z, et al. Identification and validation of circulating exosomes-based liquid biopsy for esophageal cancer. Cancer Med. 2019;8(7):3566–74.
348. Zhao Y, O’Keefe CM, Hsieh K, Cope L, Joyce SC, Pisanic TR, et al. Multiplex digital methylation-specific PCR for noninvasive screening for lung cancer. Adv Sci. 2023;10(16):e2206518.
349. Zheng G, Du L, Yang X, Zhang X, Wang L, Yang Y, et al. Serum microRNA panel as biomarkers for early diagnosis of colorectal adenocarcinoma. Br J Cancer. 2014;111(10):1985–92.
350. Zheng M, Hou L, Ma Y, Zhou L, Wang F, Cheng B, et al. Exosomal let-7d-3p and miR-30d-5p as diagnostic biomarkers for non-invasive screening of cervical cancer and its precursors. Mol Cancer. 2019;18(1):76.
351. Zvereva M, Hosen MI, Forey N, Sheikh M, Kannengiesser C, Ba I, et al. Simplex droplet digital PCR assays for the detection of TERT promoter mutations in urine samples for the non-invasive diagnosis of urothelial cancer. Methods Mol Biol. 2023;2684:213–28.
352. Zyla J, Marczyk M, Prazuch W, Sitkiewicz M, Durawa A, Jelitto M, et al. Combining low-dose computer-tomography-based radiomics and serum metabolites for diagnosis of malignant nodules in participants of lung cancer screening studies. Biomolecules. 2023;14(1):44.
353. Lin WH, Xiao J, Ye ZY, Wei DL, Zhai XH, Xu RH, et al. Circulating tumor DNA methylation marker MYO1-G for diagnosis and monitoring of colorectal cancer. Clin Epigenetics. 2021;13(1):232.
354. Peters Y, van Grinsven E, van de Haterd M, van Lankveld D, Verbakel J, Siersema PD. Individuals’ preferences for esophageal cancer screening: A discrete choice experiment. Value Health. 2020;23(8):1087–95.
355. Luque JS, Wallace K, Blankenship BF, Roos LG, Berger FG, LaPelle NR, et al. Formative research on knowledge and preferences for stool-based tests compared to colonoscopy: What patients and providers think. J Community Health. 2018;43(6):1085–92.
356. Bonello B, Ghanouni A, Bowyer HL, MacRae E, Atkin W, Halloran SP, et al. Using a hypothetical scenario to assess public preferences for colorectal surveillance following screening-detected, intermediate-risk adenomas: annual home-based stool test vs. triennial colonoscopy. BMC Gastroenterol. 2016;16(1):113.
357. Yan L, Zhao W, Yu H, Wang Y, Liu Y, Xie C. A comprehensive meta-analysis of microRNAs for predicting colorectal cancer. Medicine. 2016;95(9):e2738.
358. Kistler CE, Hess TM, Howard K, Pignone MP, Crutchfield TM, Hawley ST, et al. Older adults' preferences for colorectal cancer-screening test attributes and test choice. Patient Prefer Adherence. 2015;9:1005–16.
359. Saengow U, Chongsuwiwatvong V, Geater A, Birch S. Preferences and acceptance of colorectal cancer screening in Thailand. Asian Pac J Cancer Prev. 2015;16(6):2269–76.
360. Blanchette PS, Spreafico A, Miller FA, Chan K, Bytautas J, Kang S, et al. Genomic testing in cancer: patient knowledge, attitudes, and expectations. Cancer. 2014;120(19):3066–73.
361. Groothuis-Oudshoorn CGM, Fermont JM, van Til JA, Ijzerman MJ. Public stated preferences and predicted uptake for genome-based colorectal cancer screening. BMC Med Inform Decis Mak. 2014;14:18.
362. Ijzerman M, Broekhuizen H, Groosthuis-Oudshoon C, Vliegenthart R, Groen H. Elicitation of public preferences for lung cancer screening using three screening modalities. Ann Oncol. 2016;27(Suppl 6):VI478.
363. Blum T, Rubio I. Lecture 03: No letting up: The next EU agenda for prevention, early detection and screening. Lecture presented at: 2023; European Cancer Summit 2023.
364. Doroh VR, Delavari A, Yaseri M, Sefiddashti SE, Akbarisari A. Preferences of Iranian average risk population for colorectal cancer screening tests. Int J Health Care Qual Assur. 2019;32(4):677–87.
365. Zhu Y, Zhang H, Chen N, Hao J, Jin H, Ma X. Diagnostic value of various liquid biopsy methods for pancreatic cancer: A systematic review and meta-analysis. Medicine. 2020;99(3):e18581.
366. Garciá VR, Perestelo-Pérez L, Rivero-Santana A, Duare-Díaz A, Álvarez-Pérez Y, Torres-Castaño A, et al. PP148 liquid biopsy for the detection of ovarian or endometrial cancer in samples taken from the pap smear: PapSEEK. IJTAHC. 2021;37(S1):22–3.

**Outcomes Out of Scope (n = 6)**

367. Barnabas GD, Bahar-Shany K, Sapoznik S, Helpman L, Kadan Y, Beiner M, et al. Microvesicle proteomic profiling of uterine liquid biopsy for ovarian cancer early detection. Mol Cell Proteomics. 2019;18(5):865–75.
368. Bu J, Shim J-E, Lee TH, Cho Y-H. Multi-modal liquid biopsy platform for cancer screening: Screening both cancer-associated rare cells and cancer cell-derived vesicles on the fabric filters for a reliable liquid biopsy analysis. Nano Converg. 2019;6(1):39.
369. Carter JV, Roberts HL, Pan J, Dice JD, Burton JF, Galbraith NJ, et al. A highly predictive model for diagnosis of colorectal neoplasms using plasma microRNA: Improving specificity and sensitivity. Ann Surg. 2016;264(4):575–84.
370. Gupta P, Gulzar Z, Hsieh B, Lim A, Watson D, Mei R. Analytical validation of the CellMax platform for early detection of cancer by enumeration of rare circulating tumor cells. J Circ Biomark. 2019;8:1849454419899214.
371. Pittella-Silva F, Chin YM, Chan HT, Nagayama S, Miyauchi E, Low S-K, et al. Plasma or serum: Which is preferable for mutation detection in liquid biopsy. Clin Chem. 2020;66(7):946–57.
372. Werner S, Krause F, Rolny V, Strobl M, Morgenstern D, Datz C, et al. Evaluation of a 5-marker blood test for colorectal cancer early detection in a colorectal cancer screening setting. Clin Cancer Res. 2016;22(7):1725–33.

**Study Design Out of Scope (n = 3)**

373. Cowling T, Loshak H. An overview of liquid biopsy for screening and early detection of cancer. 2019 Nov 1. CADTH Issues in Emerging Technologies. Ottawa (ON): Canadian Agency for Drugs and Technologies in Health.
374. O’Keeffe M, Barratt A, Maher C, Zadro J, Fabbri A, Jones M, et al. Media coverage of the benefits and harms of testing the healthy: A protocol for a descriptive study. BMJ Open. 2019;9(8)e029532.
375. Ueberroth BE, Marks, LA, Borad MJ, Agrwal N. Multicancer early detection panels (MCEDs) in the primary care setting. Am J Med. 2022;135(7):e145–e149.

**Publication Type Out of Scope (n = 530)**

376. Abalde-Cela S, Piairo P, Diéguez L. The significance of circulating tumour cells in the clinic. Acta Cytol. 2019;63(6):466–78.
377. Abdayem P, Planchard D. Update on molecular pathology and role of liquid biopsy in nonsmall cell lung cancer. Eur Respir Rev. 2021;30(161):200294.
378. Abreu CM, Caballero D, Kundu SC, Reis RL. From exosomes to circulating tumor cells: Using microfluidics to detect high predictive cancer biomarkers. Adv Exp Med Biol. 2022;1379:369–87.
379. Adams E, Sepich-Poore GD, Miller-Montgomery S, knight R. using all our genomes: Blood-based liquid biopsies for the early detection of cancer. View. 2022;3(1):20200118.
380. Adashek JJ, Janku F, Kurzrock R. Signed in blood: Circulating tumor DNA in cancer diagnosis, treatment and screening. Cancers. 2021;13(14):3600.
381. Addanki S, Meas S, Sarli VN, Singh B, Lucci A. Applications of circulating tumor cells and circulating tumor DNA in precision oncology for breast cancers. Int J Mol Sci. 2022;23(14):7843.
382. Adhit KK, Wanjari A, Menon S, K S. Liquid biopsy: An evolving paradigm for non-invasive disease diagnosis and monitoring in medicine. Cureus. 2023;15(12):e50176.
383. Adriani RR, Gargari SLM. Comparative application of microfluidic systems in circulating tumor cells and extracellular vesicles isolation; A review. Biomed Microdevices. 2022;25(1):4.
384. Ahlquist DA. Universal cancer screening: revolutionary, rational, and realizable. NPJ Precis Oncol. 2018;2:23.
385. Ahlquist DA. Multi-target stool DNA test: A new high bar for noninvasive screening. Dig Dis Sci. 2015;60(3):623–33.
386. Ahmed FE. miRNA as markers for the diagnostic screening of colon cancer. Expert Rev Anticancer Ther. 2014;14(4):463–85.
387. Ain QU, Muhammad S, Hai Y, Peiling L. The role of urine and serum biomarkers in the early detection of ovarian epithelial tumours. J Obstet Gynaecol. 2022;42(8):3441–49.
388. Alam A, Ansari MA, Badrealam KF, Pathak S. Molecular approaches to lung cancer prevention. Future Oncol. 2021;17(14):1793–1810.
389. Alberca-Del Arco F, Prieto-Cuadra D, de la Blanca, RSP, Sáez-Barranquero F, Matas-Rico E, Herrera-Imbroda B. New perspectives on the role of liquid biopsy in bladder cancer: Applicability to precision medicine. Cancers. 2024;16(4):803.
390. Alexander M, Kim SY, Cheng H. Update 2020: Management of non-small cell lung cancer. Lung. 2020;198(6):897–907.
391. Alexandrou G, Mantikas KT, Allsopp R, Yapeter CA, Jahin M, Melnick T, et al. The volution of affordable technologies in liquid biopsy diagnostics: The key to clinical implementation. Cancers. 2023;15(22):5434.
392. Alimirzaie S, Bagherzadeh M, Akbari MR. Liquid biopsy in breast cancer: A comprehensive review. Clin Genet. 2019;95(6):643–60.
393. Alix-Panabières C, Pantel K. Liquid biopsy: From discovery to clinical application. Cancer Discov. 2021;11(4):858–73.
394. Almeida-Lousada H, Mestre A, Ramalhete S, Price AJ, de Mello RA, Marreiros AD, et al. Screening for colorectal cancer leading into a new decade: The “Roaring ‘20s” for epigenetic biomarkers. Curr Oncol. 2021;28(6):4874–93.
395. Alqualo NO, Campos-Fernandez E, Picolo BU, Ferreira EL, Henriques LM, Lorenti S, et al. Molecular biomarkers in prostate cancer tumorigenesis and clinical relevance. Crit Rev Oncol Hematol. 2024;194:104232.
396. Alves G, Chantre M, Delmonico L. The contribution of the 20th century discoveries on the circulating DNA as biomarkers for cancer screening. An Acad Bras Cienc. 2020;92(4):e20200919.
397. Anderson BW, Ahlquist DA. Molecular detection of gastrointestinal neoplasia: Innovations in early detection and screening. Gastroenterol Clin North Am. 2016;45(3):529–42.
398. Andersson D, Kristiansson H, Kubista M, Ståhlberg A. Ultrasensitive circulating tumor DNA analysis enables precision medicine: experimental workflow considerations. Expert Rev Mol Diagn. 2021;21(3):299–310.
399. Anonymous. MCED testing enters new diagnostic realms. Cancer Discov. 2023;13(8):1756.
400. Anonymous. Simple blood test may allow for early detection of oral cancer. Saudi Med J. 2022;43(7):765.
401. Anonymous. Screening tool for gynecologic cancers assessed. Cancer Discov. 2018;8(5):525–26.
402. Anonymous. Liquid biopsy technique may allow early screening. Cancer Discov. 2017;7(11):1207.
403. Anonymous. Advancing cancer screening with liquid biopsies. Cancer Discov. 2018;8(3):256.
404. Arechederra M, Ávila MA, Berasain C. Liquid biopsy for cancer management: A revolutionary but still limited new tool for precision medicine. Adv Lab Med. 2020;1(3):20200009.
405. Aulakh SS, Silverman DA, Young K, Dennis SK, Birkeland AC. The promise of circulating tumor DNA in head and neck cancer. Cancers. 2022;14(12):2968.
406. Baassiri A, Nassar F, Murkherji D, Shamseddine A, Nasr R, Temraz S. Exosomal non coding RNA in LIQUID biopsies as a promising biomarker for colorectal cancer. Int J Mol Sci. 2020;21(4):1398.
407. Babayan A, Pantel K. Advances in liquid biopsy approaches for early detection and monitoring of cancer. Genome Med. 2018;10(1):21.
408. Bagcchi S. Urine test can detect early stage pancreatic cancer. Lancet Oncol. 2015;16(9):e431.
409. Bagheri R, Ghorbian M, Ghorbian S. Tumor circulating biomarkers in colorectal cancer. Cancer Treat Res Commun. 2024;38:100787.
410. Baig S, Jafarey O. Liquid Biopsy – The non-invasive tool for tracking. J Coll Physicians Surg Pak. 2018;28(6):417–18.
411. Bailey JR, Aggarwal A, Imperiale TF. Colorectal cancer screening: Stool DNA and other noninvasive modalities. Gut Liver. 2016;10(2):204–11.
412. Balla A, Bhak J, Biró O. The application of circulating tumor cell and cell-free DNA liquid biopsies in ovarian cancer. Mol Cell Probes. 2022;66:101871.
413. Bao Y, Zhang D, Guo H, Ma W. Beyond blood: Advancing the frontiers of liquid biopsy in oncology and personalized medicine. Cancer Sci. 2024;115(4):1060–72.
414. Bararia A, Chakraborty P, Roy P, Chattopadhay BK, Das A, Chatterjee A, et al. Emerging role of non-invasive and liquid biopsy biomarkers in pancreatic cancer. World J Gastroenterol. 2023;29(15):2241–60.
415. Barbany G, Arthur C, Liedén A, Nordenskjöld M, Rosenquist R, Tesi B, et al. Cell-free tumour DNA testing for early detection of cancer - a potential future tool. J Intern Med. 2019;286(2):118–36.
416. Beer TM. Examining developments in multicancer early detection: highlights of new clinical data from recent conferences. Am J Manag Care. 2021;27(Suppl 19):S347–S355.
417. Beer TM. Novel blood-based early cancer detection: Diagnostics in development. Am J Manag care. 2020;26(Suppl 14):S292–S299.
418. Berger BM, Levin B, Hilsden RJ. Multitarget stool DNA for colorectal cancer screening: A review and commentary on the United States Preventive Services Draft Guidelines. World J Gastrointest Oncol. 2016;8(5):450–58.
419. Beudeker BJB, Boonstra A. Circulating biomarkers for early detection of hepatocellular carcinoma. Therap Adv Gastroenterol. 2020;13:1756284820931734.
420. Beylerli O, Gareev I, Sufianov A, Ilyasova T, Guang Y. Long noncoding RNAs as promising biomarkers in cancer. Noncoding RNA Res. 2022;7(2):66–70.
421. Bianchi DW. Unusual prenatal genomic results provide proof-of-principle of the liquid biopsy for cancer screening. Clin Chem. 2018;64(2):254–56.
422. Birknerova N, Mancikova V, Paul ED, Matyasovsky J, Cekan P, Palicka V, et al. Circulating cell-free DNA-based methylation pattern in saliva for early diagnosis of head and neck cancer. Cancers. 2022;14(19):4882.
423. Borbiev T, Kohaar I, Petrovics G. Clinical biofluid assays for prostate cancer. Cancers. 2023;16(1):165.
424. Borsetto D, Cheng J, Payne K, Nankivell P, Batis N, Rao K, et al. Surveillance of HPV-positive head and neck squamous cell carcinoma with circulating and salivary DNA biomarkers. Crit Rev Oncog. 2018;23(3–4):235–45.
425. Boulaiz H, Ramos MC, Griñán-Lisón C, García-Rubiñ0, Vicente F, Marchal JA. What's new in the diagnosis of pancreatic cancer: a patent review (2011-present). Expert Opin Ther Pat. 2017;27(12):1319–28.
426. Bradley SH, Barclay ME. "Liquid biopsy" for cancer screening. BMJ. 2021;372:m4933.
427. Bredno J, Venn O, Chen X, Freese P, Ofman JJ. Circulating tumor DNA allele fraction: A candidate biological signal for multicancer early detection tests to assess the clinical significance of cancers. Am J Pathol. 2022;192(10):1368–78.
428. Bresalier RS, Grady WM, Markowitz SD, Nielsen HJ, Batra SK, Lampe PD. Biomarkers for early detection of colorectal cancer: The early detection research network, a framework for clinical translation. Cancer Epidemiol Biomarkers Prev. 2020;29(12):2431–40.
429. Bresalier RS, Kopetz S, Brenner DE. Blood-based tests for colorectal cancer screening: do they threaten the survival of the FIT test? Dig Dis Sci. 2015;60(3):664–71.
430. Brill JV. Screening for cancer: The economic, medical, and psychosocial issues. Am J Manag Care. 2020;26 (Suppl 14):S300–S306.
431. Brito-Rocha T, Constâncio V, Henrique R, Jerónimo C. Shifting the cancer screening paradigm: The rising potential of blood-based multi-cancer early detection tests. Cells. 2023;12(6):935.
432. Broccard SP, Kasbi AA, Bagaria SP, Jones J, Shoudry M, Gabriel EM. Liquid biopsies for colorectal cancer: A narrative review of ongoing clinical trials and the current use of this technology at a comprehensive cancer center. J Gastrointest Oncol. 2022;13(1):438–49.
433. Bronkhorst AJ, Ungerer V, Holdenrieder S. Early detection of cancer using circulating tumor DNA: Biological, physiological and analytical considerations. Crit Rev Clin Lab Sci. 2019;57(4):253–69.
434. Burki TK. CA-125 blood test in early detection of ovarian cancer. Lancet Oncol. 2015;16(6):e269.
435. Bustamante P, Tsering T, Coblentz J, Mastromonaco C, Abdouh M, Fonseca C, et al. Circulating tumor DNA tracking through driver mutations as a liquid biopsy-based biomarker for uveal melanoma. J Exp Clin Cancer Res. 2021;40(1):196.
436. Cafforio P, Palmirotta R, Lovero D, Cicinelli E, Cormio G, Silvestris E, et al. Liquid biopsy in cervical cancer: Hope and pitfalls. Cancers. 2021;13(16):3968.
437. Cai G, Cai M, Feng Z, Liu R, Liang L, Zhou P, et al. A multilocus blood-based assay targeting circulating tumor DNA methylation enables early detection and early relapse prediction of colorectal cancer. Gastroenterology. 2021;161(6):2053–2056.e2.
438. Calabrese F, Lunardi F, Pezzuto F, Fortarezza F, Vuljan SE, Marquette C, et al. Are there new biomarkers in tissue and liquid biopsies for the early detection of non-small cell lung cancer? J Clin Med. 2019;8(3):414.
439. Calandri M, Siravegna G, Yevich SM, Stranieri G, Gazzera C, Kopetz S, et al. Liquid biopsy, a paradigm shift in oncology: What interventional radiologists should know. Eur Radiol. 2020;30(8):4496–503.
440. Callister MEJ, Crosbie EJ, Crosbie PAJ, Robbins HA. Evaluating multi-cancer early detection tests: An argument for the outcome of recurrence-updated stage. Br J Cancer. 2023;129(8):1209–11.
441. Cammarata G, de Miguel-Perez D, Russo A, Peleg A, Dolo V, Rolfo C, et al. Emerging noncoding RNAs contained in extracellular vesicles: Rising stars as biomarkers in lung cancer liquid biopsy. Ther Adv Med Oncol. 2022;14:17588359221131229.
442. Cani AK, Hayes DF. Breast cancer circulating tumor cells: Current clinical applications and future prospects. Clin Chem. 2024;70(1):68–80.
443. Cao M, Zhang C, Zhou L. DNA methylation detection technology and plasma-based methylation biomarkers in screening of gastrointestinal carcinoma. Epigenomics. 2021;13(16):1327–39.
444. Cardinali B, Tasso R, Piccioli P, Ciferri MC, Quarto R, Del Mastro L. Circulating miRNA in breast cancer diagnosis and prognosis. Cancers;14(9):2317.
445. Carr D, Kent DM, Welch HG. All-cause mortality as the primary endpoint for the GRAIL/National Health Service England multi-cancer screening trial. J Med Screen. 2022;29(1):3–6.
446. Carr DJ, Welch HG. Assessing the clinical utility of liquid biopsies across 5 potential indications from therapy selection to population screening: A review. JAMA Intern Med. 2023;183(10):1144–51.
447. Casangrande GMS, de Oliveira Silva M, Reis RM, Leal LF. Liquid biopsy for lung cancer: Up-to-date and perspectives for screening programs. Int J Mol Sci. 2023;24(3):2505.
448. Cavallari I, Urso L, Sharova E, Pasello G, Ciminale V. Liquid biopsy in malignant pleural mesothelioma: State of the art, pitfalls, and perspectives. Front Oncol. 2019;9:740.
449. Cecchini MJ, Yi ES. . Liquid biopsy is a valuable tool in the diagnosis and management of lung cancer. J Thorac Dis. 2020;12(11):7048–56.
450. Čelešnik H, Potočnik. Blood-based mRNA tests as emerging diagnostic tools for personalized medicine in breast cancer. Cancers. 2023;15(4):1087.
451. Chan JCH, Chow JCH, Ho CHM, Tsui TYM, Cho WC. Clinical application of circulating tumor DNA in breast cancer. J Cancer Res Clin Oncol. 2021;147(5):1431–42.
452. Chandrapalan S, Arasaradnam RP. Urine as a biological modality for colorectal cancer detection. Expert Rev Mol Diagn. 2020;20(5):489–96.
453. Chang ET, Hubbell E, Klein EA. Multicancer early detection. Clin Gastroenterol Hepatol. 2023;21(13):3464.
454. Chang L, Li J, Zhang R. Liquid biopsy for early diagnosis of non-small cell lung carcinoma: Recent research and detection technologies. Biochim Biophys Acta Rev Cancer. 2022;1877(3):188729.
455. Chang L, Ni J, Zhu Y, Pang B, Graham P, Zhang H, et al. Liquid biopsy in ovarian cancer: Recent advances in circulating extracellular vesicle detection for early diagnosis and monitoring progression. Theranostics. 2019;9(14):4130–140.
456. Chang LC, Chiu HM, Wu MS, Shen TL. The role of extracellular vesicles in the progression of colorectal cancer and its clinical applications. Int J Mol Sci. 2022;23(3):1379.
457. Chen B, Leung LL, Qu X, Chan JYK. Extracellular vesicles as biomarkers in head and neck squamous cell carcinoma: From diagnosis to disease-free survival. Cancers. 2023;15(6):1826.
458. Chen G, Zhang J, Fu Q, Taly V, Tan F. Integrative analysis of multi-omics data for liquid biopsy. Br J Cancer. 2023;128(4):505–18.
459. Chen H, Xu C, Fang Z, Mao S. Cell-free DNA microRNAs, proteins, and peptides as liquid biopsy biomarkers in prostate cancer and bladder cancer. Methods Mol Biol. 2023;2695:165–79.
460. Chen HM, Fang JY. Epigenic biomarkers for the early detection of gastrointestinal cancer. Gastrointest Tumors. 2014;1(4):201–08.
461. Chen K, Kang G, Zhao H, Zhang K, Zhang J, Yang F, et al. Liquid biopsy in newly diagnosed patients with locoregional (I-IIIA) non-small cell lung cancer. Expert Rev Mol Diagn. 2019;19(5):419–27.
462. Chen M, Hou L, Hu L, Tan C, Wang X, Bao P, et al. Platelet detection as a new liquid biopsy tool for human cancers. Front Oncol. 2022;12:983724.
463. Chen M, Zhao H. Next-generation sequencing in liquid biopsy: cancer screening and early detection. Hum Genomics. 2019;13(1):34.
464. Chen Q, Zhang ZH, Wang S, Lang JH. Circulating cell-free DNA or circulating tumor DNA in the management of ovarian and endometrial cancer. Onco Targets Ther. 2019;12:11517.
465. Chen Y, Zitello E, Guo R, Deng Y. The function of LncRNAs and their role in the prediction, diagnosis, and prognosis of lung cancer. Clin Transl Med. 2021;11(4):e367.
466. Cheng L, Zhang S, Wang M, Lopez-Beltran A. Biological and clinical perspectives of TERT promoter mutation detection on bladder cancer diagnosis and management. Hum Pathol. 2023;133:56–75.
467. Chivu-Economescu M, Necula L, Matei L, Dragu D, Bleotu C, Diaconu CC. Clinical applications of liquid biopsy in gastric cancer. Front Med. 2021;8:749250.
468. Choi WJ, Ivanics T, Gravely A, Gallinger S, Sapisochin G, O’Kane GM. Optimizing circulating tumour DNA use in the perioperative setting for intrahepatic cholangiocarcinoma: Diagnosis, screening, minimal residual disease detection and treatment response monitoring. Ann Surg Oncol. 2023;30(6):3849–63.
469. Chowdhury R, Bhatia S, Singh G, Nasreen S, De D. Circulating tumor cells: Screening and monitoring of oral cancers. J Stomatol Oral Maxillofac Surg. 2018;119(6):498–502.
470. Clarke CA, Lang K, Putcha G, Beer JP, Champagne M, Ferris A, et al. BLOODPAC: Collaborating to chart a path towards blood-based screening for early cancer detection. Clin Transl Sci. 2023;16(1):5–9.
471. Clark CA, Hubbell E, Ofman JJ. Multi-cancer early detection: A new paradigm for reducing cancer-specific and all-cause mortality. Cancer Cell. 2021;39(4):447–48.
472. Clinton T, Lotan Y. Review of the clinical approaches to the use of urine-based tumor markers in bladder cancer. Ramban Maimonides Med J. 2017;8(4):e0040.
473. Colwell J. Illumina still-off to develop early-detection test. Cancer Discov. 2016;6(4):OF3.
474. Connal S, Camceron JM, Sala A, Brennan PM, Palmer DS, Palmer JD, et al. Liquid biopsies: The future of cancer early detection. J Tralsl Med. 2023;21(1):118.
475. Constâncio V, Nunes SP, Henrique R, Jerónimo C. DNA methylation-based testing in liquid biopsies as detection and prognostic biomarkers for the four major cancer types. Cells. 2020;9(3):624.
476. Constantin N, Sina AAI, Korbie D, Trau M. Opportunities for early cancer detection: The rise of ctDNA methylation-based pan-cancer screening technologies. Epigenomes. 2022;6(1):6.
477. Coppedè F, Bhaduri U, Stoccoro A, Nicolì V, Di Venere E, Merla G. DNA methylation in the fields of prenatal diagnosis and early detection of cancers. Int J Mol Sci. 2023;24(14):11715.
478. Court CM, Ankeny JS, Hou S, Tseng HR, Tomlinsin JS. Improving pancreatic cancer diagnosis using circulating tumor cells: Prospects for staging and single-cell analysis. Expert Rev Mol Diagn. 2015;15(11):1491–504.
479. Crocetto F, Russo G, Di Zazzo E, Pisapia P, Mirto BF, Palmieri A, et al. Liquid biopsy in prostate cancer management – current challenges and future perspectives. Cancer. 2022;14(13):3272.
480. Croessmann S, Park BH. Circulating tumor DNA in early-stage breast cancer: New directions and potential clinical applications. Clin Adv Hematol Oncol. 2021;19(3):155–61.
481. Crosby D. Delivering on the promise of early detection with liquid biopsies. Br J Cancer. 2022;126(3):313–15.
482. Cunningham N, Turner NC. Liquid biopsies in breast milk for the early detection of breast cancer. Cancer Discov. 2023;13(10):2125–2127.
483. Dagogo-Jack I, Sequist LV, Piotrowska Z. The role of liquid biopsies in lung cancer screening. Semin Roentgenol. 2017;52(3):185–87.
484. Danese E, Montagnana M, Lippi G. Circulating molecular biomarkers for screening or early diagnosis of colorectal cancer: Which is ready for prime time? Ann Transl Med. 2019;7(21):610.
485. Dang DK, Park BH. Circulating tumor DNA: Current challenges for clinical utility. J Clin Invest. 2022;132(12):e154941.
486. Dao J, Conway PJ, Subramani B, Meyyappan D, Russell S, Mahadevan D. Using cfDNA and ctDNA as oncogenic markers: A path to clinical validation. Int J mol Sci. 2023;24(17):13219.
487. Dar GM, Agarwal S, Kumar A, Ninisha, Apurva, Sharma AK, et al. A non-invasive miRNA-based approach in early diagnosis and therapeutics of oral cancer. Crit Rev Oncol Hematol. 2022;180:103850.
488. David P, Mittelstädt A, Kouhestani D. Anthuber A, Kahlert C, Sohn K, et al. Current applications of liquid biopsy in gastrointestinal cancer disease – from early cancer detection to individualized treatment. Cancers. 2023;15(7):1924.
489. de Nóbrega M, Dos Reis MBD, Pereira ÉR, de Souza MF, de Syllos Cólus IM. The potential of cell-free and exosomal microRNAs as biomarkers in liquid biopsy in patients with prostate cancer. J Cancer Res Clin Oncol. 2022;148(10):2893–910.
490. De Silva S, Tennekoon KH, Karunanayake EH. Overview of the genetic basis toward early detection of breast cancer. Breast Cancer. 2019;11:71–80.
491. Deboever N, Ostrin EJ, Antonoff MB. Liquid biopsy as an adjunct to lung screening imaging. Thorac Surg Clin. 2023;33(4):411–19.
492. DeLouize A, Eick G, Karam SD, Snodgrass JJ. Current and future applications of biomarkers in samples collected through minimally invasive methods for cancer medicine and population-based research. Am J Hum Biol. 2022;34(11):e23665.
493. Deverka PA, Douglas MP, Phillips KA. Multicancer screening tests: Anticipating and addressing considerations for payer coverage and patient access. Health Aff. 2022;41(3):383–89.
494. Dholariya S, Singh RD, Sonagra A, Yadav D, Vajaria BN, Parchwani D. Integrating cutting-edge methods to oral cancer screening, analysis, and prognosis. Crit Rev Oncog. 2023;28(2):11–44.
495. Dhruva SS, Smith-Bindman R, Redberg RF. The need for randomized clinical trials demonstrating reduction in all-cause mortality with blood tests for cancer screening. JAMA Intern Med. 2023;183(10):1051–53.
496. Di Capua D, Bracken-Clark D, Rinan K, Vaird AM, Finn S. The liquid biopsy for lung cancer: State of the art, limitations and future developments. Cancers. 2021;13(16):3923.
497. Díaz-Fernández A, Ferapontova EE. Electrochemical ELASA: Improving early cancer detection and monitoring. Anal Bioanal Chem. 2023;15(18):3831–846.
498. Dickinson BT, Kisiel J, Ahlquist DA, Grady WM. Molecular markers for colorectal cancer screening. Gut. 2015;64(9):1485–494.
499. Ding J, Zhao W. The application of liquid biopsy techniques in high-risk population for hepatocellular carcinoma. Cancer Manag Res. 2022;14:2735–748.
500. Domínguez-Vigil IG, Moreno-Martínez AK, Wang JY, Roehrl MHA, Barrera-Saldaña HA. Oncotarget. 2017;9(2):2912–922.
501. Donaldson J, Park BH. Circulating tumor DNA: Measurement and clinical utility. Ann Rev Med. 2018;69:223–34.
502. Doubeni CA, Castle PE. Multicancer early detection: A promise yet to be proven. Am Fam Physician. 2023;107(3):224–225A.
503. Doubeni CA, Lau YK, Lin JS, Pennello GA, Carlson RW. Development and evaluation of safety and effectiveness of novel cancer screening tests for routine clinical use with applications to multicancer detection technologies. Cancer. 2022;128(Suppl 4):883–91.
504. Duffy MJ, Crown J. Circulating tumor DNA (ctDNA): Can it be used as a pan-cancer early detection test? Crit Rev Clin Lab Sci. 2024;61(4):241–53.
505. Duffy MJ, Lamerz R, Haglund C, Nicolini A, Kalousová M, Holubec L, et al. Tumor markers in colorectal cancer, gastric cancer and gastrointestinal stromal cancers: European group on tumor markers 2014 guidelines update. Int J Cancer. 2014;143(11):2513–522.
506. Duffy SW. All-cause mortality in multi-cancer screening trials. J Med Screen. 2022;29(1):1–2.
507. Duréndez-Sáez E, Azkárate A, Meri M, Calabuig-Fariñas S, Aguilar-Gallardo C, Blasco A, et al. New insights in non-small-cell lung cancer: Circulating tumor cells and cell-free DNA. J Thorac Dis. 2017;9(Suppl 13):S1332–S1345.
508. Durin L, Pradines A, Basset C, Ulrich B, Keller L, Dongay V, et al. Liquid biopsy of non-plasma body fluids in non-small cell lung cancer: Look closer to the tumor! Cells. 2020;9(11):2486.
509. Egatz-Gomez A, Wang C, Klacsmann F, Pan Z, Marczak S, Wang Y, et al. Future microfluidic and nanofluidic modular platforms for nucleic acid liquid biopsy in precision medicine. Biomicrofluidics. 2016;10(3):032902.
510. Eigeliene N, Saarenheimo J, Jekunen A. Potential of liquid biopsies for breast cancer screening, diagnosis, and response to treatment. Oncology. 2019;96(3):115–24.
511. Einoch Amor R, Zinger A, Broza YY, Schroeder A, Haick H. Artificially intelligent nanoarray detects various cancers by liquid biopsy of volatile markers. Adv Healthc Mater. 2022;11(17):e2200356.
512. El Achi H, Khoury JD, Loghavi S. Liquid biopsy by next-generation sequencing: A multimodality test for management of cancer. Curr Hematol Malig Rep. 2019;14(5):358–67.
513. Eslami-S Z, Cortés-Hernández LE, Alix-Panabières C. The metastatic cascade as the basis for liquid biopsy development. Front Oncol. 2020;10:1055.
514. Etzioni R, Gulati R, Weiss NS. Multicancer early detection: Learning from the past to meet the future. 2022;114(3):349–52.
515. Farooq M, Leevan E, Ahmed J, Ko B, Shin S, De Souza A, et al. Blood-based multi-cancer detection: A state-of-the-art update. Curr Probl Cancer. 2024;48:101059.
516. Febbo PG, Allo M, Alme EB, Carter GC, Dumonois R, Essig A, et al. Recommendations for equitable and widespread implementation of liquid biopsy for cancer care. JCO Precis Oncol. 2024;8:e2300382.
517. Feeney L, Harley IJ, McCluggage WG, Mullan PB, Beirne JP. Liquid biopsy in ovarian cancer: Catching the silent killer before it strikes. World J Clin Oncol. 2020;11(11):868–89.
518. Feng Z, Wu J, Lu Y, Chan YT, Zhang C, Wang D, et al. Circulating tumor cells in the early detection of human cancers. Int J Biol Sci. 2022;18(8):3251–265.
519. Ferlizza E, Solmi R, Sgarzi M, Ricciardiello L, Lauriola M. The roadmap of colorectal cancer screening. Cancers. 2021;13(5):1101.
520. Fernández-Lázaro D, Hernández JLG, Garciá AC, Del Castillo AC, Gueso MV, Cruz-Hernández JJ. Clinical perspective and translational oncology of liquid biopsy. Diagnostics. 2020;10(7):443.
521. Fernández-Lázaro D, Hernández JLG, Garciá AC, Martínez AC, Mielgo-Ayuso J, Cruz-Hernández JJ. Liquid biopsy as novel tool in precision medicine: Origins, properties, identification and clinical perspective of cancer’s biomarkers. Diagnostics. 2020;10(4):215.
522. Ferrara F, Zoupanou S, Primiceri E, Ali Z, Chiriacò MS. Beyond liquid biopsy: Toward non-invasive assays for distanced cancer diagnostics in pandemics. Biosens Bioelectron. 2022;196:113698.
523. Ferrari A, Neefs I, Hoeck S, Peeters M, Van Hal G. Towards novel non-invasive colorectal cancer screening methods: A comprehensive review. Cancers. 2021;13(8):1820.
524. Fiala C, Diamandis EP. Can a broad molecular screen based on circulating tumor DNA aid in early cancer detection. J Appl Lab Med. 2020;6(6):1372–377.
525. Fiala C, Diamandis EP. Circulating tumor DNA (ctDNA) is not a good proxy for liquid biopsies of tumor tissues for early detection. Clin Chem Lab Med. 2020;58(10):1651–653.
526. Fiala C, Diamandis EP. Utility of circulating tumor DNA in cancer diagnostics with emphasis on early detection. BMC Med. 2018;16(1):166.
527. Fiala C, Kulasingham V, Diamandis EP. Circulating tumor DNA for early cancer detection. J Appl Lab Med. 2018;3(2):300–13.
528. Foser S, Maiese K, Digumarthy SR, Puig-Butille JA, Rebhan C. Looking to the future of early detection in cancer: Liquid biopsies, imaging, and artificial intelligence. Clin Chem. 2024;70(1):27–32.
529. Franczak C, Filhine-Tresarrieu P, Gilson P, Merlin JL, Au L, Harlé A. Technical considerations for circulating tumor DNA detection in oncology. Expert Rev Mol Diagn. 2019;19(2):121–135.
530. De Freitas AJA, Causin RL, Varuzza MB, Calfa S, Filho CMTH, Komoto TT, et al. Liquid biopsy as a tool for the diagnosis, treatment, and monitoring of breast cancer. Int J Mol Sci. 2022;23(17):9952.
531. Freitas C, Sousa C, Machado F, Serino M, Santos V, Cruz-Martins N, et al. The role of liquid biopsy in early diagnosis of lung cancer. Front Oncol. 2021;11:634316.
532. Gadducci A, Cosio S. Screening for ovarian cancer in the general population: State of art and perspectives of clinical research. Anticancer Res. 2022;42(9):4207–216.
533. Gai W, Sun K. Epigenetic biomarkers in cell-free DNA and applications in liquid biopsy. Genes. 2019;10(1):32.
534. Galanopoulos M, Tsoukalas N, Pananikolaou IS, Tolia M, Gazouli M, Mantzaris GJ. Abnormal DNA methylation as a cell-free circulating DNA biomarker for colorectal cancer detection: A review of literature. World J Gastrointest Oncol. 2017;9(4):142–52.
535. Galindo Torres BP, Girón CG, Ortega RA, López PS, Leza MIA, Uche MVG. Knowledge and expectations about miRNAs as biomarkers in head and neck squamous cell cancers. Am J Otolaryngol. 2023;44(2):103771.
536. Galot R, Machiels JPH. Current applications and challenges of circulating tumor DNA (ctDNA) in squamous cell carcinoma of the head and neck (SCCHN). Cancer Treat Rev. 2020;85:101992.
537. Ganepola GA, Nizin J, Rutledge JR, Chang DH. Use of blood-based biomarkers for early diagnosis and surveillance of colorectal cancer. World J Gastrointest Oncol. 2014;6(4):83–97.
538. Gao Q, Zeng Q, Wang Z, Li C, Xu Y, Cui P, et al. Circulating cell-free DNA for cancer early detection. Innovation. 2022;3(4):100259.
539. García-Foncillas J, Alba E, Aranda E, Díaz-Rubio E, López-López R, Tabernero J, et al. Incorporating BEAMing technology as a liquid biopsy into clinical practice for the management of colorectal cancer patients: an expert taskforce review. Ann Oncol. 2017;28(12):2943–949.
540. García-Ortiz MV, Cano-Ramírez P, Toledano-Fonseca M, Aranda E, Rodríguez-Ariza A. Diagnosing and monitoring pancreatic cancer through cell-free DNA methylation: progress and prospects. Biomark Res. 2023;11(1):88.
541. Grdner L, Kostarelos K, Mallick P, Dive C, Hadjidemetriou M. Nano-omics: Nanotechnology-based multidimensional harvesting of the blood-circulating cancerome. Nat Rev Oncol. 2022;19(8):551–61.
542. Gareev I, Ahmad A, Wang J, Beilerli A, Ilyasova T, Sufianov A, et al. Gastric juice non-coding RNAs as potential biomarkers for gastric cancer. Front Physiol. 2023;14:1179582.
543. Gevilaitė, Schveigert D, Cicėnas S. Cell-free DNA in non-small cell lung cancer. Acta Med Litu. 2017;24(2):138–44.
544. Gezer U, Brinkhorst AJ, Holdenrieder S. The utility of repetitive cell-free DNA in cancer liquid biopsies. Diagnostics. 2022;12(6):1363.
545. Ghosh RK, Pandey T, Dey P. Liquid biopsy: A new avenue in pathology. Cytopathology. 2019;30(2):138–43.
546. Giannopoulou L, Lianidou ES. Liquid biopsy in ovarian cancer. Adv Clin Chem. 2020;97:13–71.
547. Glyn T, Purcell R. Circulating bacterial DNA: A new paradigm for cancer diagnostics. Front Med. 2022;9:831096.
548. Gobbini E, Swalduz A, Levra MG, Ortiz-Cuaran S, Toffart AC, Pérol M, et al. Cancers. 2020;12(11):3112.
549. Gong XY, Chen HB, Lu ZY, Zhu C, Chen DS, Chen X. Prospective role of liquid biopsy for early screening in laryngeal cancer. Invest New Drugs. 2023;41(3):376–79.
550. Grayson K, Gregory E, Khan G, Guinn BA. Urine biomarkers for the early detection of ovarian cancer – Are we there yet? Biomark Cancer. 2019;11:1179299X19830977.
551. Grinspan LT, Villanueva A. Biomarker development using liquid biopsy in hepatocellular carcinoma. Semin Liver Dis. 2022;42(2):188–201.
552. Grunvald MW, Jacobson RA, Kuzel TM, Pappas SG, Masood A. Current status of circulating tumor DNA liquid biopsy in pancreatic cancer. Int J Mol Sci. 2020;21(20):7651.
553. Guerra CE, Sharma PV, Castillo BS. Multi-cancer early detection: The new frontier in cancer early detection. Annu Rev Med. 2024;75:67–81.
554. Guibert N, Pradines A, Favre G, Mazieres J. Current and future applications of liquid biopsy in nonsmall cell lung cancer from early to advanced stages. Eur Respir Rev. 2020;29(155):190052.
555. Hackshaw A, Clarke CA, Hartman AR. New genomic technologies for multi-cancer early detection: Rethinking the scope of cancer screening. Cancer Cell. 2022;40(2):109–13.
556. Hackshaw A, Berg CD. An efficient randomised trial design for multi-cancer screening blood tests: nested enhanced mortality outcomes of screening trial. Lancet Oncol. 2021;22(10):1360–362.
557. Haferlach C, Haferlach T. Dreams can come true: The first steps toward a peripheral blood screening test for the early detection of tumors have been taken. Ann Oncol. 2019;30(1):12–13.
558. Halawa T, Baeesa S, Fadul MM, Badahdah AA, Enani M, Fathaddin AA, et al. The role of liquid biopsy in the diagnosis and prognosis of WHO Grade 4 astrocytoma. Cureus. 2023;15(6):e41221.
559. Halvaei S, Daryani S, Eslami-S Z, Samadi T, Jafarbeik-Iravani N, Bakhsayesh TO, et al. Exosomes in cancer liquid biopsy: A focus on breast cancer. Mol Ther Nucleic Acids. 2018;10:131–41.
560. Hanash SM, Ostrin EJ, Fahrmann JF. Blood based biomarkers beyond genomics for lung cancer screening. Transl Lung Cancer Res. 2018;7(3):327–35.
561. Hasenleithner SO, Speicher MR. How to detect cancer early using cell-free DNA. Cancer Cell. 2022;40(120:1464–466.
562. Hasenleithner SO, Speicher MR. A clinician's handbook for using ctDNA throughout the patient journey. Mol Cancer. 2022;21(1):81.
563. Hauptman N, Glavač D. Colorectal cancer blood-based biomarkers. Gastroenterol Res Pract. 2017;2017:2195361.
564. Heichman KA. Blood-based testing for colorectal cancer screening. Mol Diagn Ther. 2014;18(2):127–35.
565. Heidrich I, Ačkar L, Mohammadi PM, Pantel K. . Liquid biopsies: Potential and challenges. Int J Cancer. 2021;148(3):528–45.
566. Heitzer E, Perakis S, Geigl JB, Speicher MR. The potential of liquid biopsies for the early detection of cancer. NPJ Precis Oncol. 2017;1(1):36.
567. Hench IB, Hench J, Tolnay M. Liquid biopsy in clinical management of breast, lung, and colorectal cancer. Front Med. 2018;5:9.
568. Henning GM, Andriole GL, Kim EH. Liquid biomarkers for early detection of prostate cancer and summary of available data for their use in African-American men. Prostate Cancer Prostatic Dis. 2022;25(2):180–86.
569. Herrera-Pariente C, Montori S, Llach J, Bofill A, Albeniz E, Moreira L. Biomarkers for gastric cancer screening and early diagnosis. Biomedicines. 2021;9(1):1448.
570. Casillas CEH, Fernández JMF, Camceros EP, López EJH, Pacheco GL, Velázquez MM. Current status of circulating protein biomarkers to aid the early detection of lung cancer. Future Ontol. 2014;10(8):1501–515.
571. Ho J, Li X, Zhang L, Liang Y, Hu W, Yau JCW, et al. Translational genomics in pancreatic ductal adenocarcinoma: A review with re-analysis of TCGA dataset. Semin Cancer Biol. 2019;55:70–77.
572. Hofman P. Liquid biopsy for lung cancer screening: Usefulness of circulating tumor cells and other circulating blood biomarkers. Cancer Cytopathol. 2021;129(5):341–46.
573. Hofman P. Liquid biopsy for early detection of lung cancer. Curr Opin Oncol. 2017;29(1):73–8.
574. Horgan D, Čufer T, Gatto F, Lugowska I, Verbanac D, Carvalho A, et al. Accelerating the development and validation of liquid biopsy for early cancer screening and treatment tailoring. Healthcare. 2022;10(9):1714.
575. Hou J, Li XT, Xie KP. Coupled liquid biopsy and bioinformatics for pancreatic cancer early detection and precision prognostication. Mol Cancer. 2021;20(1):34.
576. Hsu CC, Wu Y. Recent advances in nanotechnology-enabled biosensors for detection of exosomes as new cancer liquid biopsy. Exp Biol Med. 2022;247(23):2152–172.
577. Hu S, Liu Y, Yang Q, Chen L, Chai H, Xiao M, et al. Liquid biopsy using cell-free DNA in the early diagnosis of hepatocellular carcinoma. Invest New Drugs. 2023;41(3):532–38.
578. Hu T, Wolfram J, Srivastava S. Extracellular vesicles in cancer detection: Hopes and hypes. Trends Cancer. 2021;7(2):122–33.
579. Huang ZB, Zhang HT, Yu B, Yu DH. Cell-free DNA as a liquid biopsy for early detection of gastric cancer. Oncol Lett. 2021;21(1):3.
580. Hyun KA, Kim J, Gwak H, Jung HI. Isolation and enrichment of circulating biomarkers for cancer screening, detection, and diagnostics. Analyst. 2016;141(2):382–92.
581. Iacob R, Mandea M, Iacob S, Pietrosanu C, Paul D, Hainaroise R, et al. Liquid biopsy in squamous cell carcinoma of the esophagus and of the head and neck. Front Med. 2022;9:827297.
582. Iannone A, Losurdo G, Pricci M, Girardi B, Massaro A, Principi M, et al. Stool investigations for colorectal cancer screening: From occult blood test to DNA analysis. J Gastrointest Cancer. 2016;47(2):143–51.
583. Ibáñez R, Mareque M, Granados R, Andía D, García-Rojo M, Quílez JC, et al. Comparative cost analysis of cervical cancer screening programme based on molecular detection of HPV in Spain. BMC Womens Health. 2021;21(1):178.
584. Ibrahim J, Peeters M, Van Camp G, de Beeck KO. Methylation biomarkers for early cancer detection and diagnosis: Current and future perspectives. Eur J Cancer. 2023;178:91–113.
585. Ijzerman MJ, de Boer J, Azad A, Degeling K, Geoghegan J, Hewitt C, et al. Towards routine implementation of liquid biopsies in cancer management: It is always too early, until suddenly it us too late. Diagnostics. 2021;11(1):103.
586. Imamura T, Komatsu S, Ichikawa D, Kawaguchi T, Miyamae M, Okajima W, et al. Liquid biopsy in patients with pancreatic cancer: Circulating tumor cells and cell-free nucleic acids. World J Gastroenterol. 2016;22(25):5627–641.
587. Ionescu F, Zhang J. Wang L. Clinical applications of liquid biopsy in prostate cancer: From screening to predictive biomarker. Cancers. 2022;14(7):1728.
588. Jacobs JW, Guarente J, Chooljian DM, Booth GS, Adkins BD. Am J Hematol. 2022;97(12):E454-E456.
589. Jacobson RA, Munding E, Hayden DM, Levy M, Kuzel TM, Pappas SG, et al. Evolving clinical utility of liquid biopsy in gastrointestinal cancers. Cancers. 2019;11(8):1164.
590. Jafari A, Karimabadi K, Rahimi A, Rostaminasab G, Khazaei M, Rezakhano L, et al. The emerging role of exosomal miRNAs as biomarkers for early cancer detection: A comprehensive literature review. Technol Cancer Res Treat. 2023;22:15330338231205999.
591. Jain G, Das P, Ranjan P, Neha, Valderrama F, Cieza-Borrella C. Urinary extracellular vesicles miRNA-A new era of prostate cancer biomarkers. Front Genet. 2023;14:1065757.
592. Jain S, Lin SY, Song W, Su YH. Urine-based liquid biopsy for nonurological cancers. Genet Test Mol Biomarkers. 2019;23(4):277–83.
593. Jiwa N, Ezzat A, Holt J, Wijayatilake DS, Takats Z, Leff DR. Nipple aspirate fluid and its use for the early detection of breast cancer. Ann Med Surg. 2022;77:103625.
594. Johnson SE, Zurn SJ, Anderson BO, Vetter BN, Katz ZB, Milner Jr DA. International perspectives on the development, application, and evaluation of a multicancer early detection strategy. Cancer. 2022;128(Suppl 4):875–82.
595. Jones J, Nguyen H, Drummond K, Morokoff A. Circulating biomarkers for glioma: A review. Neurosurgery. 2021;88(3):E221–E230.
596. Jou HJ, Ling PY, Hsu HT. Circulating tumor cells as a "real-time liquid biopsy": Recent advances and the application in ovarian cancer. Taiwan J Obstet Gynecol. 2022;61(1):34–9.
597. Kaczor-Urbanowicz KE, Cheng J, King JC, Sedarat A, Pandol SJ, Farrell JJ, et al. Reviews on current liquid biopsy for detection and management of pancreatic cancers. Pancreas. 2020;49(9):1141–152.
598. Kaczor-Urbanowicz KE, Wei F, Rao SL, Kim J, Shin H, Cheng J, et al. Clinical validity of saliva and novel technology for cancer detection. Biochim Biophys Acta Rev Cancer. 2019;1872(1):49–59.
599. Kaczor-Urbanowicz KE, Carreras-Presas CM, Kaczor T, Tu M, Wei F, Garcia-Godoy F, et al. Emerging technologies for salivaomics in cancer detection. J Cell Mol Med. 2017;21(4):640–47.
600. Kahlert C. Liquid biopsy: Is there an advantage to analyzing circulating exosomal DNA compared to cfDNA or are they the same? Cancer Res. 2019;79(10):2462–465.
601. Kaiser J. 'Liquid biopsy' for cancer promises early detection. Science. 2018;359(6373):259.
602. Kang M, Ku JH. Liquid biopsy? A recent breakthrough in noninvasive bladder cancer surveillance. Investig Clin Urol. 2016;57(5):307–08.
603. Kapeleris J, Warkiani ME, Kulasinghe A, Vela I, Kenny L, Ladwa R, et al. Clinical applications of circulating tumour cells and circulating tumour DNA in non-small cell lung cancer – An update. Front Oncol. 2022;12:859152.
604. Kashyap D, Pal D, Sharma R, Garg VK, Goel N, Koundal D, et al. Global increase in breast cancer incidence: Risk factors and preventive measures. Biomed Res Int. 2022;2022:9605439.
605. Kattner AA. The best protection is early detection: Fostering timely and accurate screening. Biomed J. 2021;44(6 Suppl 2):S155–S161.­
606. Kessler L, Le Beau MM, Smith RA, Walter FM, Wender R. The modeling of multicancer early detection (MCED) tests' residual risk and the challenges of MCED evaluation and implementation. Cancer. 2023;129(13):1966–968.
607. Kessler L, Pawar NR, Martin SS, Antalis TM, O’Connor TD. Improving cancer detection and treatment with liquid biopsies and ptDNA. Trends Cancer. 2018;4(9):643–54.
608. Keup C, Kimmig R, Kasimir-Bauer S. The diversity of liquid biopsies and their potential in breast cancer management. Cancers. 2023;15(22):5463.
609. Khalili N, Shoolo H, Hosseini N, Kazerooni AF, Familiar A, Bagheri S, et al. Adding value to liquid biopsy for brain tumors: The role of imaging. Cancers. 2023;15(21):5198.
610. Khurshid Z, Zafar MS, Khan RS, Najeeb S, Slowey PD, Rehman IU. Role of salivary biomarkers in oral cancer detection. Adv Clin Chem. 2018;86:23–70.
611. Kisiel JB, Papadopoulos N, Liu MC, Crosby D, Srivastava S, Hawk ET. Multicancer early detection test: Preclinical, translational, and clinical evidence-generation plan and provocative questions. Cancers. 2022;128(Suppl 4):861–74.
612. Klein EA, Madhavan S, Beer TM, Bettegowda C, Liu MC, Hartman AR, et al. Dying to find out: The cost of time at the dawn of the multicancer early detection era. Cancer Epidemiol Biomarkers Prev. 2023;32(8):1003–010.
613. Klein EA, Beer TM, Seiden M. The promise of multicancer early detection. Am J Med. 2023;136(3):e46–e47.
614. Klein EA, Beer TM, Seiden M. Multicancer early detection. Clin Chem Lab Med. 2022;60(5):e119–e120.
615. Koessler T, Addeo A, Nouspikel T. Implementing circulating tumor DNA analysis in a clinical laboratory: A user manual. Adv Clin Chem. 2019;89:131–88.
616. Köhn L, Johansson M, Grankvist K, Nilsson J. Liquid biopsies in lung cancer-time to implement research technologies in routine care. Ann Transl Med. 2017;5(13):278.
617. Konczalla L, Wöstemeier A, Kemper M, Karstens KF, Izbicki J, Reeh M. Clinical significance of circulating tumor cells in gastrointestinal carcinomas. Diagnostics. 2020;10(4):192.
618. Kong L, Birkeland AC. Liquid biopsies in head and neck cancer; Current state and future challenges. Cancers. 2021;13(8):1874.
619. Krebs MG, Malapelle U, André F, Paz-Ares, Schuler M, Thomas DM, et al. Practical considerations for the use of circulating tumor DNA in the treatment of patients with cancer: A narrative review. JAMA Oncol. 2022;8(12):1830–839.
620. Krishnamurthy N, Spencer E, Torkamani A, Nischolson L. Liquid biopsies for cancer: Coming to a patient near you. J Clin Med. 2017;6(1):3.
621. Kumar P, Gupta S, Das BC. Saliva as a potential non-invasive liquid biopsy for early and easy diagnosis/prognosis of head and neck cancer. Transl Oncol. 2024;40:101827.
622. Kumarasamy G, Salim NHM, Afandi NSM, Habib MAH, Amin NDM, Ismail MN, et al. Glycoproteomics-based liquid biopsy: Translational outlook for colorectal cancer clinical management in Southeast Asia. Future Oncol. 2023;19(34):2313–332.
623. Kurniali PC, Storandt MH, Jin Z. Utilization of circulating tumor cells in the management of solid tumors. J Pers Med. 2023;13(4):694.
624. Kuwatani M, Sakamoto N. Pathological and molecular diagnoses of early cancer with bile and pancreatic juice. Dig Endosc. 2022;34(7):1340–355.
625. Lam WKJ, King AD, Miller JA, Liu Z, Yu KJ, Chua MLK, et al. Recommendations for Epstein-Barr virus-based screening for nasopharyngeal cancer in high- and intermediate-risk regions. J Natl Cancer Inst. 2023;115(4):355–64.
626. Lam WKJ, Chan KCA, Lo YMD. Plasma Epstein-Barr virus DNA as an archetypal circulating tumour DNA marker. J Pathol. 2019;247(5):641–49.
627. Lamb YN, Dhillon S. Epi proColon R 2.0 CE: A blood-based screening test for colorectal cancer. Mol Diagn Ther. 2017;21(2):225–232.
628. Lee B, Lin K, Liang PS. Effectiveness and harms of colorectal cancer screening strategies. Gastrointest Endosc Clin N Am. 2022;32(2):215–26.
629. Lee MW, Kim GH, Jeon HK, Park SJ. Clinical application of circulating tumor cells in gastric cancer. Gut Liver. 2019;13(4):394–401.
630. Lee R, Robbins HA. PATHFINDER: another step on the uncharted path to multicancer screening. Lancet. 2023;402(10409):1213–215.
631. Leers MPG. Circulating tumor DNA and their added value in molecular oncology. Clin Chem Lab Med. 2020;58(2):152–61.
632. Lerner L, Winn R, Hulbert A. Lung cancer early detection and health disparities: the intersection of epigenetics and ethnicity. J Thorac Dis. 2018;10(4):2498–507.
633. Levkova M, Chervenkov T, Angelova L, Dzenkov D. Oxford nanopore technology and its application in liquid biopsies. Curr Genomics. 2023;24(6):337–44.
634. Levy B, Hu ZI, Cordova KN, Close S, Lee K, Becker D. Clinical utility of liquid diagnostic platforms in non-small cell lung cancer. Oncologist. 2016;21(9):1121–130.
635. Lewis AR, Valle JW, McNamara MG. Pancreatic cancer: Are "liquid biopsies" ready for prime-time? World J Gastroenterol. 2016;22(32):7175–785.
636. Li G, Lamsisi M, Chenafi S, Moniod L, Benlghazi A, Ennaji MM, et al. Urine-based detection of HPV for cervical cancer screening: Time for standardized tests. J Med Virol. 2023;95(4):e28737.
637. Li JH, Zhang DY, Zhu JM, Dong L. Clinical applications and perspectives of circulating tumor DNA in gastric cancer. Cancer Cell Int. 2024;24(1):13.
638. Li L, Fu K, Zhou W, Snyder M. Applying circulating tumor DNA methylation in the diagnosis of lung cancer. Precis Clin Med. 2019;2(1):45–56.
639. Li P, Liu S, Du L, Mohseni G, Zhang Y, Wang C. Liquid biopsies based on DNA methylation as biomarkers for the detection and prognosis of lung cancer. Clin Epigenetics. 2022;14(1):118.
640. Li S, Xin K, Pan S, Wang Y, Zheng J, Li Z, et al. Blood-based liquid biopsy: Insights into early detection, prediction, and treatment monitoring of bladder cancer. Cel Mol Biol Lett. 2023;28(1):28.
641. Li S, Yi M, Dong B, Tan X, Luo S, Wu K. The role of exosomes in liquid biopsy for cancer diagnosis and prognosis prediction. Int J Cancer. 2021;148(11):2640–651.
642. Liang R, Li X, Li W, Zhu X, Li C. DNA methylation in lung cancer patients: Opening a "window of life" under precision medicine. Biomed Pharmacother. 2021;144:112202.
643. Pérez-Ramírez C, Cañadas-Garre, Robles AI, Molina MÁ, Faus-Dáder MJ, Calleja-Hernández MÁ. Liquid biopsy in early stage lung cancer. Transl Lung Cancer Res. 2016;5(5):517–24.
644. Lianidou E. Detection and relevance of epigenetic markers on ctDNA: Recent advances and future outlook. Mol Oncol. 2021;15(6):1683–1700.
645. Limburg PJ, Ahlquist DA, Johnson S, Zurn SJ, Kisiel JB, Smith RA. Multicancer early detection: International summit to Clarify the Roadmap. Cancer. 2022;128(Suppl 4):859–60.
646. Lin GA, Phillips KA, Fendrick AM. Reading the crystal ball: Primary care implications while awaiting outcomes for multi-cancer early detection tests. Healthc. 2023;11(3):100705.
647. Lin KW. mSEPT9 (Epi proColon) blood test for colorectal cancer screening. Am Fam Physician. 2019;100(1):10–11.
648. Lin SY, Lineham JA, Wilson TG, Hoon DSB. Emerging utility of urinary cell0free nucleic acid biomarkers for prostate, bladder, and renal cancers. Eur Urol Focus. 2017;3(2-3):265–72.
649. Ling L, Aldoghachi AF, Chong ZX, Ho WY, Yeap SK, Chin RJ, et al. Addressing the clinical feasibility of adopting circulating miRNA for breast cancer detection, monitoring and management with artificial intelligence and machine learning platforms. Int J Mol Sci. 2022;23(23):15382.
650. Lissa D, Robles AI. Methylation analyses in liquid biopsy. Transl Lung Cancer Res. 2016;5(5):492–504.
651. Liu C, Xiang X, Han S, Lim HY, Li L, Zhang X, et al. Blood-based liquid biopsy: Insights into early detection and clinical management of lung cancer. Cancer Lett. 2022;524:91–102.
652. Liu M, Zhang Z, Zhang W, Liu SM. Advances in biomarker discovery using circulating cell-free DNA for early detection of hepatocellular carcinoma. WIREs Mech Dis. 2023;15(3):e1598.
653. Liu X, Papukashvili D, Wang Z, Liu Y, Chen X, Li J, et al. Potential utility of miRNAs for liquid biopsy in breast cancer. Front Oncol. 2022;12:940314.
654. Liu Z, Han Y, Dang Q, Xu H, Zhang Y, Duo M, et al. Roles of circulating tumor DNA in PD-1/PD-L1 immune checkpoint Inhibitors: Current evidence and future directions. Int Immunopharmacol. 2022;111:109173.
655. Lo YMD, Lam WKJ. Towards multi-cancer screening using liquid biopsies. Nat Rev Clin Oncol. 2020;17(9):525–26.
656. Locke WJ, Guanzon D, Ma C, Liew YJ, Duesing KR, Fung KYC, et al. DNA methylation cancer biomarkers: Translation to the clinic. Front Genet. 2019;10:1150.
657. Loeb S, Lilja H, Vickers A. Beyond prostate-specific antigen: Utilizing novel strategies to screen men for prostate cancer. Curr Opin Urol. 2016;26(5):459–65.
658. Loeb S, Catalona WJ. The Prostate Health Index: A new test for the detection of prostate cancer. Ther Adv Urol. 2014;6(2):74–7.
659. Loke SY, Lee ASG. The future of blood-based biomarkers for the early detection of breast cancer. Eur J Cancer. 2018;92:54–68.
660. Loktionov A. Biomarkers for detecting colorectal cancer non-invasively: DNA, RNA or proteins?. World J Gastrointest Oncol. 2020;12(2):124–48.
661. Looman-Kropp HA. Multicancer early detection tests: Where are we? JNCI Cancer Spectr. 2023;7(1):pkac084.
662. Loomans-Kropp HA, Umar A, Minasian LM, Pinsky PF. Multi-cancer early detection tests: Current progress and future perspectives. Cancer Epidemiol Biomarkers Prev. 2022;31(3):512–14.
663. Low SK, Nakamura Y. The road map of cancer precision medicine with the innovation of advanced cancer detection technology and personalized immunotherapy. Jpn J Clin Oncol. 2019;49(7):596–603.
664. Lozar T, Gersak K, Cemazar M, Kuhar CG, Jesenko T. The biology and clinical potential of circulating tumor cells. Radiol Oncol. 2019;53(2):131–47.
665. Lu T, Li J. Clinical applications of urinary cell-free DNA in cancer: Current insights and promising future. A, J Cancer Res. 2017;7(11):2318–332.
666. Lu Y, Li S, Zhu S, Gong Y, Shi J, Xu L. Methylated DNA/RNS in body fluids as biomarkers for lung cancer. Biol Proced Online. 2017;19:2.
667. Luo H, Wei W, Ye Z, Zheng J, Xu RH. Liquid biopsy of methylation biomarkers in cell-free DNA. Trends Mol Med. 2021;27(5):482–500.
668. Luo W, Rao M, Qu J, Luo D. Applications of liquid biopsy in lung cancer-diagnosis, prognosis prediction, and disease monitoring. An J Transl Res. 2018;10(12):3911–923.
669. Ma S, Zhou M, Xu Y, Gu X, Zou M, Abudushalamu G, et al. Clinical application and detection techniques of liquid biopsy in gastric cancer. Mol Cancer. 2023;22(1):7.
670. Ma X, Chen Y, Tang W, Bao H, Mo S, Liu R, et al. Multi-dimensional fragmentomic assay for ultrasensitive early detection of colorectal advanced adenoma and adenocarcinoma. J Hematol Oncol. 2021;14(1):175.
671. Macías M, Alegre E, Díaz-Lagares A, Patiño A, Pérez-Gracia JL, Sanmamed M, et al. Liquid biopsy: From basic research to clinical practice. Adv Clin Chem. 2018;83:73–119.
672. Masthan KMK, Babu NA, Dash KC, Elumalai M. Advanced diagnostic aids for oral cancer. Asian Pac J Cancer Prev. 2012;13(8):3573–576.
673. Maia MC, Salgia M, Pal SK. Harnessing cell-free DNA: Plasma circulating tumour DNA for liquid biopsy in genitourinary cancers. Nat Rev Urol. 2020;17(5):271–91.
674. Malapelle U, Pisapia P, Pepe F, Russo G, Buono M, Russo A, et al. The evolving role of liquid biopsy in lung cancer. Lung Cancer. 2022;172:53–64.
675. Malara N, Colussio ML, Grillo F, Ferrazzo T, Garo NC, Donato G, et al. Multicancer screening test based on the detection of circulating non haematological proliferating atypical cells. Mol Cancer. 2024;23(1):32.
676. Mali SB. Molecular screening of head neck cancer. Oral Oncol. 2023;144:106481.
677. Mali SB, Dahivelkar S. Liquid biopsy = Individualized cancer management: Diagnosis, monitoring treatment and checking recurrence and metastasis. Oral Oncol. 2021;123:105588.
678. Mlla M, loree JM, Kasi PM, Parikj AR. Using circulating tumor DNA in colorectal cancer: Current and evolving practices. J Clin Oncol. 2022;40(24):2846–857.
679. Maly V, Maly O, Kolostova K, Bobek V. Circulating tumor cells in diagnosis and treatment of lung cancer. In Vivo. 2019;33(4):1027–037.
680. Manea I, Iacob R, Iacob S, Cerban R, Dima S, Oniscu G, et al. Liquid biopsy for early detection of hepatocellular carcinoma. Front Med. 2023;10:18705.
681. Mann RM, Hooley R, Barr RG, Moy L. Novel approaches to screening for breast cancer. Radiology. 2020;297(2):266–85.
682. Marcuello M, Vymetalkova V, Neves RPL, Duran-Sanchon S, Vedeld HM, Tham E, et al. Circulating biomarkers for early detection and clinical management of colorectal cancer. Mol Aspects Med. 2019;69:107–22.
683. Mari R, Mamessier E, Lanbaudie E, Provansal M, Birnbaum D, Bertucci F, et al. Liquid biopsies for ovarian carcinoma: How blood tests may improve the clinical management of a deadly disease. Cancers. 2019;11(6):774.
684. Marrugo-Ramírez J, Mir M, Samitier J. Blood-based cancer biomarkers in liquid biopsy: A promising non-invasive alternative to tissue biopsy. Int J Mol Sci. 2018;19(10):2877.
685. Martini V, Timme-Bronsert S, Fichtner-Feigl S, Hoeppner J, Kulemann B. Circulating tumor cells in pancreatic cancer: Current perspectives. Cancers. 2019;11(11):1659.
686. Mastrolia I, Catani V, Oltrecolli M, Pipitone S, Vitale MG, Masciale V, et al. Chasing the role of miRNAs in RCC: From free-circulating to extracellular-vesicle-derived biomarkers. Biology. 2023;12(6):877.
687. Mathios D, Phallen J. Circulating biomarkers in glioblastoma: Ready for the prime time? Cancer J. 2021;27(5):404–09.
688. Matsuoka T, Yashiro M. Current status and perspectives of genetic testing in gastrointestinal cancer (Review). Oncol Lett. 2023;27(1):21.
689. Matsutani A, Udagawa C, Matsunaga Y, Nakamura S, Zembutsu H. Liquid biopsy for the detection of clinical biomarkers in early breast cancer: New insights and challenges. Pharmacogenomics. 2020;21(5):359–67.
690. Matuszczak M, Schalken JA, Salagierski M. Prostate cancer liquid biopsy biomarkers’ clinical utility in diagnosis and prognosis. Cancers. 2021;13(13):3373.
691. Mazouji O, Ouhajjou A, Incitti R, Mansour H. Updates on clinical use of liquid biopsy in colorectal cancer screening, diagnosis, follow-up, and treatment guidance. Front Cell Dev Biol. 2021;9:660924.
692. Meagher T. liquid biopsies and critical illness insurance: Uncomfortable bedfellows? J Insur Med. 2019;48(1):48–51.
693. Mensah M, Borzi C, Verri C, Suatoni P, Conte D, Pastorino U, et al. MicroRNA based liquid biopsy: The experience of the plasma miRNA signature classifier (MSC) for lung cancer screening. J Vis Exp. 2017;128:56236.
694. Micalizzi DS, Sequist LV, Haber DA. Deploying blood-based cancer screening. Science. 2024. 383(6681):368–70.
695. Minami S, Chikazu D, Ochiya T, Yoshioka Y. Extracellular vesicle-based liquid biopsies in cancer: Future biomarkers for oral cancer. Transl Oncol. 2023;38:101786.
696. Minasian LM, Pinsky P, Katki HA, Dickherber T, Han PKJ, Harris L, et al. Study design considerations for trials to evaluate multicancer early detection assays for clinical utility. J Natl Cancer Inst. 2023;115(3):250–57.
697. Mishra V, Singh A, Chen X, Rosenberg AJ, Pearson AT, Zhavoronkov A, et al. Application of liquid biopsy as multi-functional biomarkers in head and neck cancer. Br J Cancer. 2022;126(3):361–70.
698. Molnár B, Tóth K, Barták BK, Tulassay Z. Plasma methylated septin 9: A colorectal cancer screening marker. Expert Rev Mol Diagn. 2015;15(2):171–84.
699. Mondal SK, Whiteside TL. Proteomic profiles of melanoma cell-derived exosomes in plasma: Discovery of potential biomarkers of melanoma progression. Melanoma Res. 2021;31(5):472–75.
700. Montani F, Bianchi F. Circulating cancer biomarkers: The macro-revolution of the micro-RNA. EBioMedicine. 2016;5:4–6.
701. Moon DH, Lindsay DP, Hong S, Wang AZ. Clinical indications for, and the future of, circulating tumor cells. Adv Drug Deliv Rev. 2018;125:143–50.
702. Morrison C. Search for liquid biopsy grail points the way to drug discovery and development gems. Nat Rev Drug Discov. 2017;16(6):373–74.
703. Moser T, Kühberger S, lazzeri I, Vlachos G, Heitzer E. Bridging biological cfDNA features and machine learning approaches. Trands Genet. 2023;39(4):285–307.
704. Moul JW, Sant GR. How I Use It: The Exosome Diagnostics (EPI) prostate cancer biomarker utility in urology and primary care. Can J Urol. 2022;29(4):11224–230.
705. Nabavizadeh N. The clinical and economic benefits associated with novel multi-cancer early detection tests: Conference highlights from the 2022 ISPOR Annual Meeting. Am J Manag Care. 2022;28 (Suppl 7):S123–S132.
706. Nadal C, Winder T, Gerger A, Tougeron D. Future perspectives of circulating tumor DNA in colorectal cancer. Tumour Biol. 2017;39(5):10104283`7705749.
707. Nadauld L, Goldman DP. Considerations in the implementation of multicancer early detection tests. Future Oncol. 2022;18(28):3119–124.
708. Nadauld LD, McDonnell 3^rd^ CH, Beer TM, Liu MC, Klein EA, Hudnut A, et al. The PATHFINDER Study: Assessment of the implementation of an investigational multi-cancer early detection test into clinical practice. Cancers. 2021;12(14):3501.
709. Nagayama S, Low SK, Kiyotani K, Nakamura Y. Precision medicine for colorectal cancer with liquid biopsy and immunotherapy. Cancers. 2021;13(19):4803.
710. Najafi S, Majidpoor J, Mortezaee K. Liquid biopsy in colorectal cancer. Clin Chim Acta. 2024;553:117674.
711. Nassar FJ, Chamandi G, Tfaily MA, Zgheib NK, Nasr R. Peripheral blood-based biopsy for breast cancer risk prediction and early detection. Front Med. 2020;7:28.
712. Nebgen DR, Lu KH, Bast Jr RC. Novel approached to ovarian cancer screening. Curr Oncol Rep. 2019;21(8):75.
713. Neuhaus J, Yang B. Liquid biopsy potential biomarkers in prostate cancer. Diagnostics. 2018;8(4):68.
714. Newsome RC, Jobin C. Microbiome-derived liquid biopsy: New hope for cancer screening? Clin Chem. 2021;67(3):463–65.
715. Ng CKY, Di Costanzo GG, Terracciano LM, Piscuoglio S. Circulating cell-free DNA in hepatocellular carcinoma: Current insights and outlook. Front Med. 2018;5:78.
716. Nigro MC, Marchese PV, Deiana C, Casadio C, Galvani L, Di Federico A, et al. Clinical utility and application of liquid biopsy in lung cancer: A comprehensive review. Lung Cancer. 2023;14:11–25.
717. Nonaka T, Wong DTW. Saliva diagnostics: Salivaomics, saliva exosomics, and saliva liquid biopsy. J Am Dent Assoc. 2023;154(8):696–704.
718. Nonaka T, Wong DTW. Liquid biopsy in head and neck cancer: Promises and challenges. J Dent Res. 2018;97(6):701–08.
719. Nordgård O, Tjensvoll K, Gilje B, Søreide K. Circulating tumour cells and DNA as liquid biopsies in gastrointestinal cancer. Br J Surg. 2018;105(2):e110–e120.
720. Novellis P, Cominesi SR, Rossetti F, Mondoni M, Gregorc V, Veronesi G. Lung cancer screening: who pays? Who receives? The European perspectives. Transl Lung Cancer Res. 2021;10(5):2395–406.
721. Ofman JJ, Fendrick AM, Raza A. Novel multicancer early detection technology-potential value to employers and the workforce. Am J Manag Care. 2020;26(10 Spec No.):SP363.
722. Old R, Pharoah R, Wald N. NHS announces a pilot of a blood test for early detection of many cancers. J Med Screen. 2021;28(1):1–2.
723. Olivier T, Gill J, Prasad V. Multi-cancer screening tests: Communicating about risks should be prioritized. Am J Med. 2022;135(4):413–15.
724. ÓrdenesP P, Pardo CC, Elizondo-Vega R, Oyarce K. Current research on molecular biomarkers for colorectal cancer in stool samples. Biology. 2023;13(1):15.
725. Ossandon MR, Agrawal L, Bernhard EJ, Conley BA, Dey SM, Divi RL, et al. Circulating tumor DNA assays in clinical cancer research. J Natl Cancer Inst. 2018;110(9):929–34.
726. Otandault A, Anker P, Dache ZAA, Guillaumon V, Meddeb R, Pastor B, et al. Recent advances in circulating nucleic acids in oncology. Ann Oncol. 2019;30(3):374–84.
727. Owens L, Gogebakan KC, Menon U, Gulati R, Weiss NS, Etzioni R. Short-term endpoints for cancer screening trials: Does tumor subtype matter? Cancer Epidemiol Biomarkers Prev. 2023;32(6):741–43.
728. Ozawa PMM, Jucoski TS, Vieira E, Carvalho TM, Malgeiros D, Ribeiro EMSF. Liquid biopsy for breast cancer using extracellular vesicles and cell-free microRNAs as biomarkers. Transl Res. 2020;223:40–60.
729. Palaz F, Kalkan AK, Can Ö, Demir AN, Tozluyurt A, Özacn A, et al. CRISPR-Cas13 system as a promising and versatile tool for cancer diagnosis, therapy, and research. ACS Synth Biol. 2021;10(6):1245–267.
730. Palmirotta R, Lovero D, Cafforio P, Felici C, Mannavola F, Pellè E, et al. Liquid biopsy of cancer: A multimodal diagnostic tool in clinical oncology. Ther Adv Med Oncol. 2018;10:1758835918794630.
731. Panta P, Venna VR. Salivary RNA signatures in oral cancer detection. Anal Cell Pathol. 2014;2014:450629.
732. Pantel K. Blood tests for early detection of lung cancer: Challenges and promises. Lancet Respir Med. 2020;8(7):654–56.
733. Pantel K, Hille C, Scher HI. Circulating tumor cells in prostate cancer: From discovery to clinical utility. Clin Chem. 2019;65(1):87–99.
734. Paramathas S, Guha T, Pugh TJ, Malkin D, Villani A. Considerations for the use of circulating tumor DNA sequencing as a screening tool in cancer predisposition syndromes. Pediatr Blood Cancer. 2020;67(12):e28758.
735. Parida P, Baburaj G, Rao M, Lewis S, Damerla RR. Circulating cell-free DNA as a diagnostic and prognostic marker for cervical cancer. Int J Gynecol Cancer. 2023:ijgc-2023-004873.
736. Parikh RB, Prasad V. Blood-based screening for colon cancer: A disruptive innovation or simply a disruption? JAMA. 2016;314(23):2519–20.
737. Pathak N, Dodds J, Zamora J. Could urine testing be the future of cervical cancer screening? Womens Health. 2015;11(3):265–67.
738. Peek LJ, Jett JR. A blood test for early detection of lung cancer. MLO Med Lab Obs. 2016;48(12):18–21.
739. Pei H, Li L, Han Z, Wang Y, Tang B. Recent advances in microfluidic technologies for circulating tumor cells: enrichment, single-cell analysis, and liquid biopsy for clinical applications. Lab Chip. 2020;20(21):3854–875.
740. Peng M, Chan C, Hulbert A, Brock MV, Yu F. Non-blood circulating tumor DNA detection in cancer. Oncotarget. 2017;8(40):69162–173.
741. Peng S, Zhang X, Wu Y. Potential applications of DNA methylation testing technology in female tumors and screening methods. Biochim Biophys Acta Rev Cancer. 2023;1878(5):188941.
742. Pankova A, Kuziakova O, Gulaia V, Tiasto V, Goncharov NV, Lanskikh D, et al. Comprehensive clinical assays for molecular diagnostics of gliomas: The current state and future prospects. Front Mol Biosci. 2023;10:1216102.
743. Pérez-Ruiz E, Gutiérrez V, Muñoz M, Oliver J, Sánchez M, Gálvez-Carvajal L, et al. Liquid biopsy as a tool for the characterisation and early detection of the field cancerization effect in patients with oral cavity carcinoma. Biomedicines. 2021;9(19):1478.
744. Petranovic M, Raoof S, Digumarthy SR, Sharma A, Shepard JAO, Gainor JF, et al. Liquid biopsy, diagnostic imaging, and future synergies. J Am Coll Radiol. 2022;2Pt B):336–43.
745. Phalguni A, Seaman H, Routh K, Halloran S, Simpson S. Tests detecting biomarkers for screening of colorectal cancer: What is on the horizon. GMS Health Technol Assess. 2015;11:Doc01.
746. Pietrasz D, Sereni E, Lancelotti F, Pea A, Luchini C, Innamorati G, et al. Circulating tumour DNA: A challenging innovation to develop "precision onco-surgery" in pancreatic adenocarcinoma. Br J Cancer. 2022;126(12):1676–683.
747. Pisapia P, Costa JL, Pepe F, Russo G, Gragnano G, Russo A, et al. Next generation sequencing for liquid biopsy based testing in non-small cell lung cancer in 2021. Crit Rev Oncol Hematol. 2021;161:103311.
748. Poggiana C, Rossi E, Zamarchi R. Possible role of circulating tumor cells in early detection of lung cancer. J Thorac Dis. 2020;12(7):3821–835.
749. Pogliaghi G. Liquid biopsy in thyroid cancer: From circulating biomarkers to a new prospective of tumor monitoring and therapy. Minerva Endocrinol. 2021;46(1):45–61.
750. Pös O, Biró O, Szemes T, Nagy B. Circulating cell-free nucleic acids: Characteristics and applications. Eur J Hum Genet. 2018;26(7):937–45.
751. Post C, Braun TP, Etzioni R, Nabavizadeh N. Multicancer early detection tests: An overview of early results from prospective clinical studies and opportunities for oncologists. JCO Oncol Pract. 2023;19(12):1111–115.
752. Pourali G, Khalili-Tanha G, Nazari E, Maftooh M, Nassiri M, Hassanian SM, et al. Circulating tumor cells and cell-free nucleic acids as biomarkers in colorectal cancer, Curr Pharm Des. 2023;29(10):748–65.
753. Powter B, Jefferys SA, Sareen H, Cooper A, Brungs D, Po J, et al. Human TERT promoter mutations as a prognostic biomarker in glioma. J Cancer Res Clin Oncol. 2021;147(4):1007–017.
754. Pox CP. Controversies in colorectal cancer screening. Digestion. 2014;89(4):274–81.
755. Pratt VM. Are we ready for a blood-based test to detect colon cancer? Clin Chem. 2014;60(9):1141–142.
756. Punnen S, Pavan N, Parekh DJ. Finding the wolf in sheep’s clothing: The 4Kscore is a novel blood test that can accurately identify the risk of aggressive prostate cancer. Rev Urol. 2015;17(1):3–13.
757. Putcha G, Gutierrez A, Skates S. Multicancer screening: One size does not fit all. JCO Precis Oncol. 2021;5:574–76.
758. Qu C, Jiao Y, Wang Y. A liquid biopsy assay for identifying early-stage hepatocellular carcinoma in asymptomatic HBsAg-seropositive individuals. Mel Cell Oncol. 2019;6(5):e1614419.
759. Quircio L, Orso F. The power of microRNAs as diagnostic and prognostic biomarkers in liquid biopsies. Cancer Drug Resist. 2020;3(2):117–39.
760. Rahbari M, Rahbari N, Reissfelder C, Weitz J, Kahlert C. Exosomes: novel implications in diagnosis and treatment of gastrointestinal cancer. Langenbecks Arch Surg. 2016;401(8):1097–110.
761. Rai V, Abdo J, Agrawal DK. Biomarkers for early detection, prognosis, and therapeutics of esophageal cancers. Int J Mol Sci. 2023;24(4):3316.
762. Rank KM, Shaukat A. Stool based testing for colorectal cancer: An overview of available evidence. Curr Gastroenterol Rep. 2017;19(8):39.
763. Ransohoff DF. Evaluating a new cancer screening blood test: Unintended consequences and the need for clarity in policy making. J Natl Cancer Inst. 2021;113(2):109–11.
764. Raoof S, Klein EA. Further discussion regarding multicancer early detection tests. Am Fam Physician. 2024;109(2):104.
765. Raufi AG, May MS, Hadfield MJ, Seyhan AA, El-Deiry WS. Advances in liquid biopsy technology and implications for pancreatic cancer. Int J Mol Sci. 2023;24(4):4238.
766. Rayamajhi S, Sipes J, Tetlow AL, Saha S, Bansal A, Godwin AK. Extracellular vesicles as liquid biopsy biomarkers across the cancer journey: From early detection to recurrence. Clin Chem. 2024;70(1):206–19.
767. Razzaghi H, Khabbazpour M, Heidary Z, Heiat M, Moghaddam ZS, Derogar P, et al. Emerging role of tumor-educated platelets as a new liquid biopsy tool for colorectal cancer. Arch Iran Med. 2023;26(8):447–54.
768. Ren J, Liu R. The implication of liquid biopsy in the non-small cell lung cancer: Potential and expectation. Methods Mol Biol. 2023;2695:145–63.
769. Repetto O, Vettori R, Steffan A, Cannizzaro R, De Re V. Circulating proteins as diagnostic markers in gastric cancer. Int J Mol Sci. 2023;24(23):16931.
770. Revelo AE, Martin A, Velasquez R, Kulandaisamy PC, Bustamante J, Keshishyan S, et al. Liquid biopsy for lung cancers: An update on recent developments. Ann Transl Med. 2019;7(15):349.
771. Ribeiro IP, de melo JB, Carreira IM. Head and neck cancer: Searching for genomic and epigenetic biomarkers in body fluids - the state of art. Mol Cytogenet. 2019;12:33.
772. Rijavec E, Coco S, Genova C, Rossi G, Longo L, Grossi F. Liquid biopsy in non-small cell lung cancer: Highlights and challenges. Cancers. 2019;12(10:17.
773. Riva F, Dronov OI, Khomenko DI, Huguet F, Louvet C, Mariani P, et al. Clinical applications of circulating tumor DNA and circulating tumor cells in pancreatic cancer. Mol Oncol. 2016;10(3):481–93.
774. Rodriguez-Casanova A, Costa-Fraga N, Bao-Caamano A, López-López R, Muinelo-Romay L, Diaz-Lagares. Epigenetic landscape of liquid biopsy in colorectal cancer. Front Cell Dev Biol. 2021;9:622459.
775. Rogucki M, Buczyńska A, Krętowski AJ, Popławska-Kita A. The importance of miRNA in the diagnosis and prognosis of papillary thyroid cancer. J Clin Med. 2021;10(20):4738.
776. Rolfo C, Russo A. Moving forward liquid biopsy in early liver cancer detection. Cancer Discov. 2023;13(3):532–34.
777. Root A, Allen P, Tempst P, Yu K. Protein biomarkers for early detection of pancreatic ductal adenocarcinoma: Progress and challenges. Cancers. 2018;10(3):67.
778. Rossi SH, Stewart GD. Re: Clinical validation of a targeted methylation-based multi-cancer early detection test using an independent validation set. Eur Urol. 2022;82(4):442–43.
779. Roy D, Pascher A, Juratli MA, Sporn JC. The potential of aptamer-medicated liquid biopsy for early detection of cancer. Int J Mol Sci. 2021;22(11):5601.
780. Saini A, Pershad Y, Albadawi H, Kuo M, Alzubaidi S, Naidu S, et al. Liquid biopsy in gastrointestinal cancers. Diagnostics. 2018;8(4):75.
781. Sankar K, Zeinali M, Nagrath S, Ramnath N. Molecular biomarkers and liquid biopsies in lung cancer. Semin Oncol. 2022:S0093-7754(22)0047-1.
782. Sant M, Bernat-Peguera A, Felip E, Margelí M. Role of ctDNA in breast cancer. Cancers. 2022;14(2):310.
783. Santarpia M, Liguori A, D’Aveni A, Karachialiou N, Gonzalez-Cao M, Daffinà MG, et al. Liquid biopsy for lung cancer early detection. J Thorac Dis. 2018;10(Suppl 7):S882–897.
784. Santini D, Botticelli A, Galvano A, Iuliani M, Incorvaia L, Gristina V, et al. Network approach in liquidomics landscape. J Exp Clin Cancer Res. 2023;42(1):193.
785. Saplacan RMM, Mircea PA, Balacescu L, Balacescu O. MicroRNAs as non-invasive screening biomarkers of colorectal cancer. Clujul Med. 2015;88(4):453–56.
786. Sarangi S, Mosulpuria K, Higgins MJ, Bardia A. The evolving role of circulating tumor cells in the personalized management of breast cancer: From enumeration to molecular characterization. Curr Breast Cancer Rep. 2014;6(3):146–53.
787. Sauter ER. Reliable biomarkers to identify new and recurrent cancer. Eur J Breast Health. 2017;13(4):162–67.
788. Sawada K, Kotani D, Bando H. The clinical landscape of circulating tumor DNA in gastrointestinal malignancies. Front Oncol. 2018;8:263.
789. Scarlotta M, Simsek C, Kim AK. Liquid biopsy in solid malignancy. Genet Test Mol Biomarkers. 2019;23(4):284–96.
790. Schear RM, Hoyos JM, Davis AQ, Woods PL, Poblete S, Richardson RN, et al. Patient engagement and advocacy considerations in development and implementation of a multicancer early detection program. Cancer. 2022;128 (Suppl 4):909–17.
791. Schmitz-Dräger BJ, Droller M, Lokeshwar VB, Lotan Y, Hudson MA, van Rhijn, et al. Molecular markers for bladder cancer screening, early diagnosis, and surveillance: The WHO/ICUD consensus. Urol Int. 2015;94(1):1–24.
792. Schreuders EH, Grobbee EJ, Spaander MCW, Kuipers EJ. Advances in fecal tests for colorectal cancer screening. Curr Treat Options Gastroenterol. 2016;14(1):152–62.
793. Schwich E, Rebmann V. The inner and outer qualities of extracellular vesicles for translational purposes in breast cancer. Front Immunol. 2018;9:584.
794. Selby K, Elwyn G, Volk RJ. Multi-cancer early detection tests, primary care, and shared decision making. Ann Intern Med. 2023;176(5):718–20.
795. Serrano MJ, Garrido-Navas MC, Mochon JJD, Cristofanilli M, Gil-Bazo I, Pauwels P, et al. Precision prevention and cancer interception: The new challenges of liquid biopsy. Cancer Discov. 2020;10(11):1635–644.
796. Shaukat A, Levin TR. Current and future colorectal cancer screening strategies. Nat Rev Gastroenterol Hepatol. 2022;19(8):521–31.
797. Shbeer AM, Robadi IA. Liquid biopsy holds a promising approach for the early detection of cancer: Current information and future perspectives. Pathol Res Pract. 2024;254:155082.
798. Shen Y, Shi R, Zhao R, Wang H. Clinical application of liquid biopsy in endometrial carcinoma. Med Oncol. 2023;40(3):92.
799. Shiao MS, Chang JM, Lertkhachonsuk AA, Rermluk N, Jinawath N. Circulating exosomal miRNAs as biomarkers in epithelial ovarian cancer. Biomedicines. 2021;9(10):1433.
800. Shinozuka T, Kanda M, Kodera Y. Site-specific protein biomarkers in gastric cancer: A comprehensive review of novel biomarkers and clinical applications. Expert Rev Mol Diagn. 2023;23(8):701–12.
801. Shirley M. Epi proColon® for colorectal cancer screening: A profile of its use in the USA. Mol Diagn Ther. 2020;24(4):497–503.
802. Shoda K, Saito R, Maruyama S, Furuya S, Akaike H, Kawaguchi Y, et al. Liquid biopsy as a perioperative biomarker of digestive tract cancers: Review of the literature. Surg Today. 2021;51(6):849–61.
803. Singh A, Gupta S, Sachan M. Epigenetic biomarkers in the management of ovarian cancer: Current prospectives. Front Cell Dev Biol. 2019;7:182.
804. Singh AP, Cheng H, Guo X, Levy B, Halmos B. Circulating tumor DNA in non-small-cell lung cancer: A primer for the clinician. JCO Precis Oncol. 2017;1:1–13.
805. Słomka A, Wang B, Mocan T, Horhat A, Willms AG, Schmidt-Wolf IGH, et al. Extracellular vesicles and circulating tumour cells – Complementary liquid biopsies or standalone concepts. Theranostics. 2022;12(13):5836–855.
806. Smania MA. Liquid biopsy for cancer screening, diagnosis, and treatment. J Am Assoc Nurse Pract. 2020;32(1):5–7.
807. Smith RA, Lam AK. Liquid biopsy for investigation of cancer DNA in esophageal squamous cell carcinoma. Methods Mol Biol. 2020;2129:203–15.
808. Smolle E, Taucher V, Lindenmann J, Pichler M, Smolle-Juettner FM. Liquid biopsy in non-small cell lung cancer-current status and future outlook-a narrative review. Transl Lung Cancer Res. 2021;10(5):2237–251.
809. Snow A, Chen D, Lang JE. The current status of the clinical utility of liquid biopsies in cancer. Expert Rev Mol Diagn. 2019;19(11):1031–041.
810. Song LL, Li YM. Current noninvasive tests for colorectal cancer screening: An overview of colorectal cancer screening tests. World J Gastrointest Oncol. 2016;8(11):793–800.
811. Spector ME, Farlow JL, Haring CT, Brenner JC, Birkeland AC. The potential for liquid biopsies in head and neck cancer. Discov Med. 2018;25(139):251–57.
812. Špilak A, Brachner A, Kegler U, Neuhaus W, Noehammer C. Implications and pitfalls for cancer diagnostics exploiting extracellular vesicles. Adv Drug Deliv Rev. 2021;175:113819.
813. Stejskal P, Goodarzi H, Srovnal J, Hajdúch M, van’t Veer L, Magbanua MJM. Circulating tumor nucleic acids: Biology, release mechanisms, and clinical relevance. Mol Cancer. 2023;22(1):15.
814. Takai E, Yachida S. Circulating tumor DNA as a liquid biopsy target for detection of pancreatic cancer. World J Gastroenterol. 2016;22(38):8480–488.
815. Tan R, Phua SKA, Soong YL, Oon LLE, Chan KS, Lucky SS, et al. Clinical utility of Epstein-Barr virus DNA and other liquid biopsy markers in nasopharyngeal carcinoma. Cancer Commun. 2020;40(11):564–85.
816. Tan WY, Sharma A, Das P, Ahuja N. Early detection of cancers in the era of precision oncology. Curr Opin Oncol. 2023;35(2):115–24.
817. Tang CM, Zhu P, Li S, Makarova OV, Amstutz PT, Adams DL. Blood-based biopsies-clinical utility beyond circulating tumor cells. Cytometry A. 2018;93(12):1246–250.
818. Tatischeff I. Current search through liquid biopsy of effective biomarkers for early cancer diagnosis into the rich cargoes of extracellular vesicles. Int J Mol Sci. 2021;22(11):5674.
819. Tavares NT, Lobo J, Bagrodia A. MicroRNAs for detecting occult genitourinary cancer. Curr Opin Urol. 2024;34(1):20–6.
820. The Lancet Oncology. Cancer detection: The quest for a single liquid biopsy for all. Lancet Oncol. 2020;21(6):733.
821. Theil G, Fornara P, Bialek J. Position of circulating tumor cells in the clinical routine in prostate cancer and breast cancer patients. Cancers. 2020;12(12):3782.
822. Thierry AR. A step closer to cancer screening by blood test. Clin Chem. 2018;64(10):1420–422.
823. Thorat MA. Liquid biopsy for cancer diagnosis and screening - The promise and challenges. Ann Clin Biochem. 2019;56(4):420–23.
824. To YH, Lee B, Wong HL, Gibbs P, Tie J. Circulating tumour DNA to guide treatment of gastrointestinal malignancies. Visc Med. 36(5):388–96.
825. Tomar U, Grover N, Tomar S, Bhalla K, Singh S. Liquid biopsy and its significance in tumour - Detection in the field of pathology. J Oral Maxillofac Pathol. 2023;27(1):195–200.
826. Tomasik B, Skrzypski M, Bieńkowski M, Dziadziuszko R, Jassem J. Current and future applications of liquid biopsy in non-small-cell lung cancer-a narrative review. Transl Lung Cancer Res. 2023;12(3):594–614.
827. Torresan S, de Scordilli M, Bortolot M, Di Nardo P, Foltran L, Fumagallu A, et al. . Liquid biopsy in colorectal cancer: Onward and upward. Crit Rev Oncol Hematol. 2024;194:104242.
828. Toth 3^rd^ JF, Trivedi M, Gupta S. Screening for colorectal cancer: The role of clinical laboratories. Clin Chem. 2024;70(1):150–64.
829. Tóth K, Barták BK, Tulassay Z, Molnár B. Circulating cell-free nucleic acids as biomarkers in colorectal cancer screening and diagnosis. Expert Rev Mol Diagn. 2016;16(2):239–52.
830. Trinidad CV, Tetlow AL, Bantis LE, Godwin AK. Reducing ovarian cancer mortality through early detection: Approaches using circulating biomarkers. Cancer Prev Res. 2020;13(3):241–52.
831. Turano M, Delrio P, Rega D, Cammarota F, Polverino A, Duraturo F, et al. Promising colorectal cancer biomarkers for precision prevention and therapy. Cancers. 2019;11(12):1932.
832. Tzanikou E, Lianidou E. The potential of ctDNA analysis in breast cancer. Crit Rev Clin Lab Sci. 2020;57(1):54–72.
833. Guimarães CTU, Martina NNF, da Silva Oliveira KC, Almeida CM, Pinheira TM, Gigek CO, et al. Liquid biopsy provides new insights into gastric cancer. Oncotarget. 2018;9(19):15144–56.
834. Umapathy VR, Natarajan PM, Swamikannu B. Review insights on salivary proteomics biomarkers in oral cancer detection and diagnosis. Molecules. 2023;28(13):5283.
835. Underwood JJ, Quadri RS, Kalva SP, Shah H, Sanjeevaiah AR, Beg MS, et al. Liquid biopsy for cancer: Review and implications for the radiologist. Radiology. 2020;294(1):5–17.
836. Vacante M, Ciuni R, Basile F, Biondi A. The liquid biopsy in the management of colorectal cancer: An overview. Biomedicines. 2020;8(9):308.
837. van der Pol Y, Mouliere F. Toward the early detection of cancer by decoding the epigenetic and environmental fingerprints of cell-free DNA. Cancer Cell. 2019;36(4):350–68.
838. van Ginkel JH, Huibers MMH, Noorlag R, de Bree R, van Es RJJ, Willems SM. Liquid biopsy: A future tool for posttreatment surveillance in head and neck cancer? Pathobiology. 2017;84(3):115–20.
839. Van Keer S, Pattyn J, Tjalma WAA, Van Ostade X, Ieven M, Van Damme P, et al. First-void urine: A potential biomarker source for triage of high-risk human papillomavirus infected women. Eur J Obstet Gynecol Reprod Biol. 2017;216:1–11.
840. Vatandoost N, Ghanbari J, Mojaver M, Avan Am Ghayour-Mobarhan M, Nedaeinia R, et al. Early detection of colorectal cancer: From conventional methods to novel biomarkers. J Cancer Res Clin Oncol. 2016;142(2):341–51.
841. Wadowska K, Bil-Lula I, Trembecki Ł, Śliwińska-Mossoń M. genetic markers in lung cancer diagnosis: A review. Int J Mol Sci. 2020;21(13):4569.
842. Wang J, Han X, Sun Y. DNA methylation signatures in circulating cell-free DNA as biomarkers for the early detection of cancer. Sci China Life Sci. 2017;60(4):356–62.
843. Wang W, Zhen S, Ping Y, Wang L, Zhang Y. Metabolomic biomarkers in liquid biopsy: accurate cancer diagnosis and prognosis monitoring. Front Oncol. 2024;14:1331215.
844. Wang Y, Wang Z, Gang X, Wang G. Liquid biopsy in prostate cancer: current status and future challenges of clinical application. Aging Male. 2021;24(1):58–71.
845. Wang Y, Chen PM, Liu RB. Advance in plasma SEPT9 gene methylation assay for colorectal cancer early detection. World J Gastrointest Oncol. 2018;10(1):15–22.
846. Warton K, Samimi G. Methylation of cell-free circulating DNA in the diagnosis of cancer. Front Mol Biosci. 2015;2:13.
847. Wantanabe K, Nakamura Y, Low SK. Clinical implementation and current advancement of blood liquid biopsy in cancer. J Hum Genet. 2021;66(9):909–26.
848. Watts G. Liquid biopsy: Still early days for early detection. Lancet. 2018;391(10140):2493–593.
849. Wei J, Liu X, Li T, Xing P, Zhang C, Yang J. The new horizon of liquid biopsy in sarcoma: The potential utility of circulating tumor nucleic acids. J Cancer. 2020;11(18):5293–308.
850. Wei Y, Yang W, Huang Q, Chen Y, Zeng K, Chen J, et al. Clinical significance of circulating tumor cell (CTC)-specific microRNA (miRNA) in breast cancer. Prog Biophys Mol Biol. 2023;177:229–34.
851. Welch HG, Dey T. Testing whether cancer screening saves lives: Implications for randomized clinical trials of multicancer screening. JAMA Intern Med. 2023;83(11):1255–258.
852. Weng J, Xiang X, Ding L, Wong ALA, Zeng Q, Sethi G, et al. Extracellular vesicles, the cornerstone of next-generation cancer diagnosis? Semin Cancer Biol. 2021;74:105–120.
853. Wentsensen N, Clarke MA. Liquid biopsy for cancer detection: Clinical and epidemiologic considerations. Clin Cancer Res. 2021;27(21):5733–5735.
854. Worm Ørntoft MB. Review of blood-based colorectal cancer screening: How far are circulating cell-free DNA methylation markers from clinical implementation? Clin Colorectal Cancer. 2018;17(2):e415–e433.
855. Wu C, Zhang J, Li H, Xu W, Zhang X. The potential of liquid biopsies in gastrointestinal cancer. Clin Biochem. 2020;84:1–12.
856. Wurdinger T, Veld SGJGI, Best MG. Platelet RNA as pan-tumor biomarker for cancer detection. Cancer Res. 2020;80(7):1371–373.
857. Wurst T. Liquid biopsies. Genet Test Mol Biomarkers. 2018;22(4):207–208.
858. Xiao S, Yao Y, Liao S, Xu B, Li X, Zhang Y, et al. Accurate and convenient lung cancer diagnosis through detection of extracellular vesicle membrane proteins via Förster Resonance energy transfer. Nano Lett. 2023;23(17):8115–125.
859. Xie H, Kim RD. The application of circulating tumor DNA in the screening, surveillance, and treatment monitoring of colorectal cancer. Ann Surg Oncol. 2021;28(3):1845–858.
860. Xin L, Yue Y, Zihan R, Youbin C, Tianyu L, Rui W. Clinical application of liquid biopsy based on circulating tumor DNA in non-small cell lung cancer. Front Physiol. 2023;14:1200124.
861. Xue VW, Wong CSC, Cho WCS. Early detection and monitoring of cancer in liquid biopsy: Advances and challenges. Expert Rev Mol Diagn. 2019;19(4):273–76.
862. Yadav A, Kumar A, Siddiqui MH. Detection of circulating tumour cells in colorectal cancer: Emerging techniques and clinical implications. World J Clin Oncol. 2021;12(12):1169–181.
863. Yamada NO, Senda T. Circulating microRNA-92a-3p in colorectal cancer: A review. Med Mol Morphol. 2021;54(3):193–202.
864. Yamamoto H, Watanabe Y, Sato Y, Maehata T, Itoh F. Non-invasive early molecular detection of gastric cancers. Cancers. 2020;12(10):2880.
865. Yan YY, Guo QR, Wang FH, Adhikari R, Zhu ZY, Zhang HY, et al. Cell-free DNA: Hope and potential application in cancer. Front Cell Dev Biol. 2021;9:639233.
866. Yang H, Li W, Ren L, Yang Y, Zhang Y, Ge B, et al. Progress on diagnostic and prognostic markers of pancreatic cancer. Oncol Res. 2023;31(2):83–99.
867. Yang WY, Feng LF, Meng X, Chen R, Xu WH, Hou J, et al. Liquid biopsy in head and neck squamous cell carcinoma: circulating tumor cells, circulating tumor DNA, and exosomes. Expert Rev Mol Diagn. 2020;20(120:1213–227.
868. Yee NS, Zhang S, He HZ, Zheng SY. Extracellular vesicles as potential biomarkers for early detection and diagnosis of pancreatic cancer. Biomedicines. 2020;8(12):581.
869. Yeo D, Bastian A, Strauss H, Saxena P, Grimison P, Rasko JEJ. Exploring the clinical utility of pancreatic cancer circulating tumor cells. Int J Mol Sci. 2022;23(3):1671.
870. Yi X, Ma J, Guan Y, Chen R, Yang L, Xia X. The feasibility of using mutation detection in ctDNA to assess tumor dynamics. Int J Cancer. 2017;140(120:2642–647.
871. Yörüker EE, Holdenrieder S, Gezer U. Blood-based biomarkers for diagnosis, prognosis and treatment of colorectal cancer. Clin Chim Acta. 2016;455:26–32.
872. Yu N, Zhou J, Cui F, Tang X. Circulating tumor cells in lung cancer: Detection methods and clinical applications. Lung. 2015;193(20:157–71.
873. Yu W, Hurley J, Roberts D, Chakrabortty SK, Enderle D, Noerholm N, et al. Exosome-based liquid biopsies in cancer: Opportunities and challenges. Ann Oncol. 2021;32(4):466–77.
874. Zhang B, Chen Z, Tao B, Yi C, Lin Z, Li Y, et al. m^6^A target microRNAs in serum for cancer detection. Mol Cancer. 2021;20(1):170.
875. Zhang K, Fu R, Liu R, Su Z. Circulating cell-free DNA-based multi-cancer early detection. Trends Cancer. 2024;10(2):161–74.
876. Zhang X, Hou H, Jiang M, Zhang X. Aberrant circulating tumor DNA methylation and exosomal microRNA biomarkers for early detection of colorectal cancer. Mol Biol Rep. 2023;50(3):2743–750.
877. Zhang X, Li B. Updates of liquid biopsy in oral cancer and multiomics analysis. Oral Dis. 2023;29(1):51–61.
878. Zhang Y, Wang Y, Su X, Wang P, Lin W. The value of circulating RNA in cancer diagnosis, monitoring, prognosis, and guiding treatment. Front Oncol. 2021;11:736546.
879. Zhao Y, Tang J, Jiang K, Liu SY, Aicher A, Heeschen C. Liquid biopsy in pancreatic cancer - Current perspective and future outlook. Biochim Biophys Acta Rev Cancer. 2023;1878(3):188868.
880. Zheng H, Wu X, Yin J, Wang S, Li Z, You C. Clinical applications of liquid biopsies for early lung cancer detection. Am J Cancer Res. 2019;9(12):2567–579.
881. Zheng Y, Vioix H, Liu FX, Singh B, Sharma S, Sharda D. Diagnostic and economic value of biomarker testing for targetable mutations in non-small-cell lung cancer: A literature review. Future Oncol. 2022;18(4):505–18.
882. Zhou B, Xu JW, Cheng YG, Gao JY, Hu SY, Wang L, et al. Early detection of pancreatic cancer: Where are we now and where are we going? Int J Cancer. 2017;141(2):231–41.
883. Zieren RC, Zondervan PJ, Pienta KJ, Bex A, de Reijke TM, Bins AD. Diagnostic liquid biopsy biomarkers in renal cell cancer. Nat Rev Urol. 2024;21(3):133–57.
884. Žilovič D, Čiurlienė R, Sabaliauskaitė R, Jarmalaitė S. Future screening prospects for ovarian cancer. Cancers. 2021;13(15):3840.
885. Zou J, Xiao Z, Wu Y, Yang J, Cui N. Noninvasive fecal testing for colorectal cancer. Clin Chim Acta. 2022;524:123–31.
886. Zubor P, Kubatka P, Kajo K, Dankova Z, Polacek H, Bielik T, et al. Why the gold standard approach by mammography demands extension by multiomics? Application of liquid biopsy miRNA profiles to breast cancer disease management. Int J Mol Sci. 2019;20(12):2878.
887. Crosby D, Lyons N, Greenwood E, Harrison S, Him S, Moffat J, et al. A roadmap for the early detection and diagnosis of cancer. Lancet Oncol. 2020;21(11):1397–399.
888. Dhanasekaren R, Adeniji N. Current and emerging tools for hepatocellular carcinoma surveillance. Hepatol Commun. 2021;5(12):1972–86.
889. Alix-Panabières C, Pantel K. Clinical applications of circulating tumor cells and circulating tumor DNA as liquid biopsy. Cancer Discov. 2016;6(5):479–91.
890. Amaral MJ, Oliveria RC, Donato P, Tralhão JG. Pancreatic cancer biomarkers: Oncogenic mutations, tissue and liquid biopsies, and radiomics – A review. Dig Dis Sci. 2023;68(7):2811–823.
891. Cisneros-Villanueva M, Hidalgo-Pérez L, Rios-Romero M, Cedro-Tanda A, Ruiz-Villavicencio CA, Page K, et al. Cell-free DNA analysis in current cancer clinical trails: A review. Br J Cancer. 2022;126(3):391–400.
892. Dama E, Colangelo T, Fina E, Cremonesi M, Kallikourdis M, Veronesi G, et al. Biomarkers and lung cancer early detection: State of the art. Cancers. 2021;13(15):3919.
893. Farncombe KM, Wong D, Norman ML, Oldfield LE, Sobotka JA, Basik M, et al. Current and new frontiers in hereditary cancer surveillance: Opportunities for liquid biopsy. Am J Hum Genet. 2023;110(10):1616–27.
894. Filipska M, Rosell R. Mutilated circulating tumor DNA as a liquid biopsy in lung cancer detection and treatment. Mol Oncol. 2021;15(6):1667–82.
895. Galoş D, Gorzo A, Balacescu O, Sur D. Clinical applications of liquid biopsy in colorectal cancer screening: Current challenges and future perspectives. Cells. 2022;11(21):3493.
896. Herbst J, Pantel K, Effenberger K, Wikman H. Clinical applications and utility of cell-free DNA-based liquid biopsy analyses in cervical cancer and its precursor lesions. Br J Cancer. 2022;127(8):1403–410.
897. Huang L, Rong Y, Tang X, Yi K, Wu J, Wang F. Circular RNAs are promising biomarkers in liquid biopsy for the diagnosis of non-small cell lung cancer. Front Mol Biosci. 2021;8:625722.
898. Igari F, Tanaka H, Giuliano AE. The applications of plasma cell-free DNA in cancer detection: Implications in the management of breast cancer patients. Crit Rev Oncol Hematol. 2022;175:103725.
899. Mannelli C. Tissue vs liquid biopsies for cancer detection: Ethical issues. J Bioeth Inq. 2019;16(4):551–57.
900. Tonini V, Zanni M. Early diagnosis of pancreatic cancer: What strategies to avoid a foretold catastrophe. World J Gastroenterol. 2022;28(31):4235–48.
901. Trigg RM, Shaw JA, Turner SD. Opportunities and challenges of circulating biomarkers in neuroblastoma. Open Biol. 2019;9(5):1900056.
902. Trujillo B, Wu A, Wetterskog D, Attard G. Blood-based liquid biopsies for prostate cancer: Clinical opportunities and challenges. Br J Cancer. 2022;127(8):1394–402.
903. Vandekerckhove O, Cuppens K, Pat K, Du Pont B, Froyen G, Maes B. Liquid biopsy in early-stage lung cancer: Current and future clinical applications. Cancers. 2023;15(10):2702.
904. Wang S, Yang M, Li R, Bai J. Current advances in noninvasive methods for the diagnosis of oral squamous cell carcinoma: A review. Eur J Med Res. 2023;28(1):53.
905. Wang W, Zheng Z, Lei J. CTC, ctDNA, and exosome in thyroid cancers: A review. Int J Mol Sci. 2023;24(18):13767.
906. Yang J, Xu R, Wang C, Qui J, Ren B, You L. Early screening and diagnosis strategies of pancreatic cancer: A comprehensive review. Cancer Commun. 2021;41(12):1257–74.

**Context Out of Scope (n = 4)**

907. Kayamba V, Zyambo K, Kelly P. Presence of blood in gastric juice: A sensitive marker for gastric cancer screening in a poor resource setting. PLoS One. 2018;13(10):e0205185.
908. Nagarkar R, Gopichand M, Pal SK, Gupta A, Saquib N. Sagar G, et al. The high sensitivity of the multi-cancer detection test ONCOVERYX-F offers a promising platform for ovarian cancer screening. Int J Womens Health. 2024;16:1–7.
909. Nawaz I, Moumad K, Martorelli D, Ennaji MM, Zhou X, Zhang Z, et al. Detection of nasopharyngeal carcinoma in Morocco (North Africa) using a multiplex methylation-specific PCR biomarker assay. Clin Epigenetics. 2015;7(1):89.
910. Shrestha B, Vaidya KM, Joshi R. Evaluation of visual inspection of cervix with acetic acid and liquid based in cervical cancer screening with cervical biopsy. J Nepal Health Res Counc. 2020;18(3):426–30.

**Publication Language Out of Scope (n = 1)**

911. Zhang S-Y, Qin S, Li G-H, Yi Y-Q, Fu H-J, Gao Y-J, et al. Detection of peripheral blood circulating tumor cells in oral squamous cell carcinoma and its clinical significance. Hua Xi Kou Qiang Yi Xue Za Zhi. 2021;39(5):591–97.

**Missed Duplicates (n = 2)**

912. Su WC, Kao WY, Chang TK, Tsai HL, Huang CW, Chen YC, et al. Stool DNA test targeting methylated syndecan-2 (SDC2) as a noninvasive screening method for colorectal cancer. Biosci Rep. 2021;41(1):BSR20201930.
913. Symonds EL, Hughes D, Flight I, Woodman R, Chen G, Ratcliffe J, et al. A randomized controlled trial testing provision of fecal blood test options on participation for colorectal cancer screening. Cancer Prev Res. 2019;12(9):631–40.

**Captured by Included Systematic Review (n = 35)**

914. Adler A, Geiger S, Keil A, Bias H, Schatz P, deVos T, et al. Improving compliance to colorectal cancer screening using blood and stool based tests in patients refusing screening colonoscopy in Germany. BMC gastroenterol. 2014;14:183.
915. Alborelli I, Generali D, Jermann P, Cappelletti MR, Ferrero G, Scaggiante B, et al. Cell-free DNA analysis in healthy individuals by next-generation sequencing: A proof of concept and technical validation study. Cell Death Dis. 2019;10(7):534.
916. Brenner H, Chen H. Fecal occult blood versus DNA testing: Indirect comparison in a colorectal cancer screening population. Clin Epidemiol. 2017;9:377–84.
917. Chabon JJ, Hamilton EG, Kurtz DM, Esfahani MS, Moding EJ, Stehr H, et al. Integrating genomic features for non-invasive early lung cancer detection. Nature. 2020;580(7802):245–51.
918. Chen X, Dong Z, Hubbell E, Kurtzman KN, Oxnard GR, Venn O, et al. Prognostic significance of blood-based multi-cancer detection in plasma cell-free DNA. Clin Cancer Res. 2021;27(15):4221–29.
919. Chen X, Gole J, Gore A, He Q, Lu M, Min J, et al. Non-invasive early detection of cancer four years before conventional diagnosis using a blood test. Nat Commun. 2020;11(1):3475.
920. Church TR, Wandell M, Lofton-Day C, Mongin SJ, Burger M, Payne SR, et al. Prospective evaluation of methylated SEPT9 in plasma for detection of asymptomatic colorectal cancer. Gut. 2014;63(2):317–25.
921. Cohen JD, Lu L, Wang Y, Thoburn C, Afsari B, Danilova L, et al. Detection and localization of surgically resectable cancers with a multi-analyte blood test. Science. 2018;359(6378):926–30.
922. Constâncio V, Nunes SP, Moreira-Barbosa C, Freitas R, Oliveira J, Pousa I, et al. Early detection of the major male cancer types in blood-based liquid biopsies using a DNA methylation panel. Clin Epigenetics. 2019;11(1):175.
923. Gao Q, Lin YP, Li BS, Wang GQ, Dong LQ, Shen BY, et al. Unintrusive multi-cancer detection by circulating cell-free DNA methylation sequencing (THUNDER): Development and independent validation studies. Ann Oncol. 2023;34(5):486–95.
924. Hacksaw A, Cohen SS, Reichert H, Kansal AR, Chung KC, Ofman JJ. Estimating the population health impact of a multi-cancer early detection genomic blood test to complement existing screening in the US and UK. Br J Cancer. 2021;125(10):1432–42.
925. Izumi D, Zhu Z, Chen Y, Toden S, Huo X, Kanda M, et al. Assessment of the diagnostic efficiency of a liquid biopsy assay for early detection of gastric cancer. JAMA Netw Open. 2021;4(8):e2121129.
926. Johnson DA, Barclay RL, Mergener K, Weiss G, König T, Beck J, et al. Plasma Septin9 versus fecal immunochemical testing for colorectal cancer screening: A prospective multicenter study. PLoS One. 2014;9(6):e98238.
927. Kapoor R, So JBY, Zhu F, Too HP, Yeoh KG, Yoong JSY. Evaluating the use of microRNA blood tests for gastric cancer screening in a stratified population-level screening program: An early model-based cost-effectiveness analysis. Value Health. 2020;23(9):1171–79.
928. Klein EA, Richards D, Cohn A, Tummala M, Lapham R, Cosgrove D, et al. Clinical validation of a targeted methylation-based multi-cancer early detection test using an independent validation set. Ann Oncol. 2021;32(9):1167–77.
929. Lennon AM, Buchanan AH, Kinde I, Warren A, Honushefsky A, Cohain AT, et al. Feasibility of blood testing combined with PET-CT to screen for cancer and guide intervention. Science. 369(6499):eabb9601.
930. Lipscomb J, Horton S, Kuo A, Tomasetti C. Evaluating the impact of multicancer early detection testing on health and economic outcomes: Toward a decision modeling strategy. Cancer. 2022;128 (Suppl 4):892–908.
931. Liu MC, Oxnard GR, Klein EA, Swanton C, Seiden MV, CCGA Consortium. Sensitive and specific multi-cancer detection and localization using methylation signatures in cell-free DNA. Ann Oncol. 2020;31(6):745–59.
932. Nunes SP, Moreira-Barbosa C, Salta S, de Sousa SP, Pousa I, Olivereira J, et al. Cell-free DNA methylation of selected genes allows for early detection of the major cancers in women. Cancers. 2018;10(10):357.
933. Peterse EFP, Meester RGS, de Jonge L, Omidvari AH, Alarid-Escudero F, Knudsen AB, et al. Comparing the cost-effectiveness of innovative colorectal cancer screening tests. J Natl Cancer Inst. 2021;113(2):154–61.
934. Qu C, Wang Y, Wang P, Chen K, Wang M, Zeng H, et al. Detection of early-stage hepatocellular carcinoma in asymptomatic HBsAg-seropositive individuals by liquid biopsy. Proc Natl Acad Sci USA. 2019;116(13):6308–12.
935. Ris F, Hellan M, Douissard J, Nieva JJ, Triponez F, Woo Y, et al. Blood-based multi-cancer detection using a novel variant calling assay (DEEPGEN^TM^): Early clinical results. Cancers. 2021;13(16):4104.
936. Symonds EL, Highes D, Flight I, Woodman R, Chen G, Ratcliffe J, et al. A randomized controlled trial testing provision of fecal and blood test options on participation for colorectal cancer screening. Cancer Prev Res. 2019;12(9):631–40.
937. Tafazzoli A, Ramsey SD, Shaul A, Chaven A, Ye W, Kansel AR, et al. The potential value-based price of a multi-cancer early detection genomic blood test to complement current single cancer screening in the USA. Pharmacoeconomics. 2022;40(11):1107–17.
938. Tang WHW, Yimer H, Tummala M, Shao S, Chung G, Clement J, et al. Performance of a targeted methylation-based multi-cancer early detection test by race and ethnicity. Prev Med. 2023;167:107384.
939. van der Poort EKJ, van Ravesteyn NT, van den Broek JJ, de Koning HJ. The early detection of breast cancer using liquid biopsies: Model estimated of the benefits, harms and costs. Cancers. 2022;14(12):2951.
940. Yang D, Hillman SL, Harris AM, Sinicrope PS, Devens ME, Ahlquist DA. Patient perceptions of stool DNA testing for pan-digestive cancer screening: a survey questionnaire. World J Gastroenterol. 2014;20(17):4972–79.
941. Young GP, Chen G, Wilson CJ, McGrane E, Hughes-Barton DLA, Flight IHK, et al. “Rescue” of nonparticipants in colorectal cancer screening: A randomized controlled trial of three noninvasive test options. Cancer Prev Res. 2021;14(8):803–10.
942. Zhao Z, Wang Y, Wu W, Yang Y, Du L, Dong H. Cost-effectiveness of low-dose computed tomography with a plasma-based biomarker for lung cancer screening in China. JAMA Netw Open. 2022;5(5):e2213634.
943. So JBY, Kapoor R, Zhu F, Koh C, Zhou L, Zou R, et al. Development and validation of a serum microRNA biomarker panel for detecting gastric cancer in a high-risk population. Gut. 2021;70(5):829–37.
944. Imperiale TF, Ransohoff DF, Itzkowitz SH, Levin TR, Lavin P, Lidgard GP, et al. Multitarget stool DNA testing for colorectal-cancer screening. N Eng J Med. 2014;370(14):1287–97.
945. Abola MV, Fennimore TF, Chen MM, Chen Z, Sheth AK, Cooper G, et al. Stool DNA-based versus colonoscopy-based colorectal cancer screening: patient perceptions and preferences. Fam Med Community Health. 2015;3(3):2–8.
946. Liles EG, Coronado GD, Perrin N, Harte AH, Nungesser R, Quigley N, et al. Uptake of a colorectal cancer screening blood test is higher than of a fecal test offered in clinic: A randomized trial. Cancer Treat Res Commun. 2017;10:27–31.
947. Deibel A, Deng L, Cheng CY, Schlander M, Ran T, Lang B, et al. Evaluating key characteristics of ideal colorectal cancer screening modalities: The microsimulation approach. Gastrointest Endosc. 2021;94(2):379–390.e7.

**Full-text Not Available (n = 9)**

948. Aalami AH, Aalami F, Aliabadi EK, Amirabadi A, Sahebkar A. Detection of circulating cell-free DNA to diagnose hepatocellular carcinoma in Chinese population: A systematic review and meta-analysis. Curr Med Chem. 2023.
949. Bhargava A, Bunkar N, Aglawe A, Pandey KC, Tiwari R, Chaudhury K, et al. Epigenetic biomarkers for risk assessment of particulate matter associated lung cancer. Cur Drug Targets. 2018;19(10):1127–47.
950. El Kadmiri N. Advances in early detection of colorectal cancer: A focus on non-invasive biomarkers. Curr Drug Targets. 2021;22(9):1043–53.
951. Hoare M, Fitzgerald RC. What is the current status of multicancer early detection tests and upper gastrointestinal cancer? Lancet Gastroenterol Hepatol. 2023;8(12):1065–66.
952. Loft M, To YH, Gibbs P, Tie J. Clinical application of circulating tumour DNA in colorectal cancer. Lancet Gastroenterol Hepatol. 2023;8(9):837–52.
953. Memarpour S, Khalili-Tanha G, Ghannad AA, Razavi MS, Joudi M, Joodi M, et al. The clinical application of circulating tumor cells and DNAs as prognostic and predictive biomarkers in gastrointestinal cancer. Curr Cancer Drug Targets. 2021;21(8):676–88.
954. Perdas E, Stawski R, Nowak D, Zubrzycka M. Potential of liquid biopsy in papillary thyroid carcinoma in context of miRNA, BRAF and p53 mutation. Curr Drug Targets. 2018;19(14):1721–29.
955. Rodon N, Trias I, Verdú M, Calvo M, Banus JM, Puig X. Correlation of mRNA-PCA3 urine levels with the new grading system in prostate cancer. Rev Esp Patol. 2019;52(1):20–6.
956. Soda N, Rehm BHA, Sonar P, Nguyen NT, Shiddiky MJA. Advanced liquid biopsy technologies for circulating biomarker detection. J Mater Chem B. 2019;7(43):6670–704.

**Supplementary Figure S1: Types of biological fluid sampled in included studies**

Number of Studies

| Biological Fluid Sampled | Number of Studies |
| --- | --- |
| Blood | 77 |
| Stool | 28 |
| Urine | 9 |
| Saliva | 2 |
| Sputum | 1 |
| Uterine lavage | 1 |
| Nipple fluid | 1 |

Note that studies could have investigated more than one biological fluid. The blood category included
studies which specified the sample as blood, plasma or serum.

## Supplementary Table S6: Ongoing clinical trials examining the use of liquid biopsy and MCED tests for the purposes of cancer screening in asymptomatic populations

| **Trial Name**  **(Registration)** | **Study Design** | **Aim** | **Sample Size** | **Population** | **Primary Outcome(s)** | **Status**  **(Estimated End date)** |
| --- | --- | --- | --- | --- | --- | --- |
| **Experimental** | | | | | | |
| A prospective interventional study on early screening for lung cancer using liquid biopsy  (NCT06422637) | RCT | To compare the performance of the MERCURY model (including liquid biopsy) with LDCT for the early detection of lung cancer. | 5,944 | Aged 40–75 and capable of signing the study consent form. | - PPV | Not yet recruiting  (December 2027) |
| Implementing fragmentomics into real world screening interventions to evaluate clinical utility among individuals with elevated risk for lung cancer: FIRSTLungL301  (NCT06145750) | RCT | To determine whether the availability of a DNA-based fragmentomics test to physicians affects overall lung cancer screening rates. | 90 | Primary care physicians that care for a population of at least 50 individuals eligible for lung cancer screening. | - Number of CT scans received | Recruiting  (June 2026) |
| Evaluation of multi-cancer early detection testing in a high-risk population: The INFORM Study  (NCT06450171) | Single group assignment | To evaluate the potential benefits and harms of cancer screening with a blood-based MCED test (Galleri). | 1,000 | Aged ≥22 at high-risk for cancer (family history suggestive of elevated cancer risk or diagnosed with a cancer predisposition syndrome). | - Cancer detection rate | Not yet recruiting  (January 2028) |
| Evaluation of multi-cancer early detection testing in a high-risk military population The Sentinel Study  (NCT06523868) | Single group assignment | To assess the performance of the Galleri MCED test for cancer screening in a military population. | 1,500 | Active-duty military service members aged ≥45 years with at least 8 years of military service with no cancer diagnosis. | - Specificity - PPV - NPV - NNS | Not yet recruiting  (September 2028) |
| Feasibility of cell-free DNA liquid biopsy in screening high-risk patients for lung cancer  (NCT05384769) | Parallel assignment | To determine the feasibility of using liquid biopsy for lung cancer screening in a community setting. | 108 | Asymptomatic individuals aged 50–80 years with a smoking history of ≥20 pack years and if quit, quit within the last 15 years. | - Screening preference (liquid biopsy or LDCT) | Recruiting  (January 2026) |
| **Observational** | | | | | | |
| Assessment of early-detection based on liquid biopsy in biliary tract cancer: ASCEND-BTC  (NCT05667350) | Case-control | To detect early biliary tract cancer by using assays combining serum protein and cfDNA methylation, | 492 | Individuals age 40–75 years with pathologically confirmed biliary tract cancers and controls with a confirmed diagnosis of benign biliary tract diseases. | - Sensitivity - Specificity | Recruiting  (June 2024) |
| Assessment of early-detection based on liquid biopsy in intracranial tumors: ASCEND-BRAIN  (NCT05679089) | Case-control | To assess performance of a cfDNA methylation-based early detection model for intracranial tumours. | 358 | Aged 40–75 with pathologically confirmed malignant intracranial tumour, benign disorders of the nervous system, and healthy controls. | - Sensitivity - Specificity | Recruiting  (June 2024) |
| Early-stage detection of liver, biliary tract and pancreatic cancers: LIBRARY  (NCT06139042) | Case-control | To detect early liver, biliary tract, and pancreatic cancers through a combination of cfDNA methylation, miRNA, and serum protein. | 1,608 | Treatment naïve individuals aged 40–75 years with pathologically confirmed liver, biliary tract, and pancreatic cancers and healthy controls. | - Sensitivity - Specificity | Recruiting  (December 2025) |
| Exploring a breast cancer early screening model based on cfDNA  (NCT06016790) | Case-control | To use cfDNA multi-omics to explore a new breast cancer early detection model. | 480 | Individuals ages 18–70 with diagnosed breast cancer and controls with breast fibroma or breast adenosis. | - Sensitivity - Specificity | Recruiting  (May 2024) |
| Performance of multi-cancer early-detection based on various biomarkers in female cancers: PERCEIVE-II  (NCT06001099) | Case-control | To validate a multi-omitcs assay for the early detection of gynecologic cancers. | 2,935 | Aged ≥18 years with a confirmed diagnosis or highly suspicious case of gynecologic malignancies and healthy controls. | - Sensitivity - Specificity - CSO accuracy | Recruiting  (December 2024) |
| Clinical validation of freenome multiomics blood test for lung cancer screening: PROACT-LUNG  (NCT06122077) | Prospective cohort | To evaluate the performance of the Freenome Multiomics Blood Test in individuals eligible for lung cancer screening. | 20,000 | Aged ≥50 years current or former smokers with a ≥20 pack-year smoking history. | - Sensitivity - Specificity | Recruiting  (June 2027) |
| Early detection of five common cancers using the ctDNA analysing test: K-DETEK  (NCT05227261) | Prospective cohort | To evaluate the performance of a blood ctDNA test for the early detection of cancer. | 10,000 | Aged ≥40 at enrollment with no clinical suspicion of cancer or history of cancer. | - Sensitivity - Specificity - PPV - NPV | Recruiting  (December 2023) |
| LEVANTIS-0093A: GAGomes for multi-cancer early detection in high-risk adults  (NCT05295017) | Prospective cohort | The validate the performance of free GAGome-based test in a high-risk population. | 1,256 | Adults aged 55–80 with a significant smoking history (current or past). | - Sensitivity - Specificity | Recruiting  (March 2025) |
| MCED in Firefighters  (NCT05780957) | Prospective cohort | To explore the performance of urine and blood tests for MCED in firefighters. | 2,000 | Actively working adult firefighters. | - Sensitivity - Specificity - PPV - NPV | Recruiting  (December 2023) |
| Oncoliq: Early breast cancer detection based on liquid biopsies and microRNAs  (NCT06439940) | Prospective cohort | To compare the results of the Oncoliq liquid biopsy test mammography for the detection of breast cancer. | 364 | Individuals >35 years with no history of cancer visiting the study gynecological clinic for annual mammographic check-up. | - Levels of miRNAs in blood samples | Recruiting  (March 2025) |
| Oncoliq: Test for early breast cancer detection  (NCT04906330) | Prospective cohort | To determine the accuracy of the Oncoliq test for the early detection of breast cancer. | 500 | Women between 50 – 70 years without a personal history of oncological disease. | - miRNA levels in plasma | Recruiting  (August 2025) |
| Prospective screening and differentiating common cancers using peripheral blood cfDNA sequencing  (NCT06036563) | Prospective cohort | To screen for and differentiate common cancers in those with and without suspicious lesions. | 3,200 | Aged 20–80 with a pathologically confirmed cancer diagnosis and individuals who have not been diagnosed with cancer. | - Accuracy - Sensitivity - Specificity | Recruiting  (December 2025) |
| REFLECTION: A clinical practice learning program for Galleri  (NCT05205967) | Prospective cohort | To describe the cancer detection and signal detection within and across sites in individuals who receive the Galleri MCED test. | 17,000 | Aged ≥22 years whose healthcare provider has ordered a Galleri test. | - Cancer detection rate - Sensitivity - Specificity - PPV - Cancer stage - CSO accuracy | Recruiting  (August 2028) |
| The Jingling Cohort  (NCT06011694) | Prospective cohort | To assess the performance and clinical utility of an MCED test (MERCURY) in an average-risk Chinese population. | 150,000 | Individuals aged 45–75 at average-risk for cancer. | - Sensitivity - Specificity - PPV - NPV | Recruiting  (May 2027) |
| Urothelial cancer screening in individuals with Lynch syndrome using a urine tumor DNA panel: LS-URO Study  (NCT06218433) | Prospective cohort | To employ a urine DNA marker to identify asymptomatic cancers in individuals with Lynch syndrome. | 200 | Aged 50–75 with a diagnosis of Lynch syndrome. | - Sensitivity - Specificity | Recruiting  (December 2034) |
| LEVANTIS-0087A: GAGomes for multi-cancer early detection in asymptomatic adults  (NCT05235009) | Retrospective cohort | To validate the performance of a free GAGome-based MCED test. | 9,170 | Asymptomatic adults aged 35–80 years with no recent history of cancer. | - Sensitivity - Specificity | Recruiting  (March 2025) |

CSO = cancer signal origin; CT = computed tomography; ctDNA = circulating tumour DNA; cfDNA = cell-free DNA; DNA = deoxyribonucleic acid; LDCT = low-dose computed tomography; MCED = multi-cancer early detection; miRNA = micro ribonucleic acid; NPV = negative predictive value; NNS = number needed to screen; PPV = positive predictive value; RCT = randomized controlled trial
